# Analyses of caspase-1-regulated transcriptomes in various tissues lead to identification of novel IL-1β-, IL-18- and sirtuin-1-independent pathways

**DOI:** 10.1186/s13045-017-0406-2

**Published:** 2017-02-02

**Authors:** Ya-feng Li, Gayani Nanayakkara, Yu Sun, Xinyuan Li, Luqiao Wang, Ramon Cueto, Ying Shao, Hangfei Fu, Candice Johnson, Jiali Cheng, Xiongwen Chen, Wenhui Hu, Jun Yu, Eric T. Choi, Hong Wang, Xiao-feng Yang

**Affiliations:** 1 Centers for Metabolic Disease Research and Cardiovascular Research, Lewis Katz School of Medicine at Temple University, 3500 North Broad Street, MERB-1059, Philadelphia, PA 19140 USA; 2 Cardiovascular Research, & Thrombosis Research, Departments of Pharmacology, Lewis Katz School of Medicine at Temple University, Philadelphia, PA 19140 USA; 3 Department of Physiology, Lewis Katz School of Medicine at Temple University, Philadelphia, PA 19140 USA; 4 Department of Immunology, Lewis Katz School of Medicine at Temple University, Philadelphia, PA 19140 USA; 5 Department of Surgery, Lewis Katz School of Medicine at Temple University, Philadelphia, PA 19140 USA; 6 The Shanxi Provincial People’s Hospital, an Affiliate Hospital of Shanxi Medical University, Taiyuan, Shanxi 030001 China

**Keywords:** Caspase-1, Microarray datasets, Meta-analysis, Inflammation and transcriptome

## Abstract

**Background:**

It is well established that caspase-1 exerts its biological activities through its downstream targets such as IL-1β, IL-18, and Sirt-1. The microarray datasets derived from various caspase-1 knockout tissues indicated that caspase-1 can significantly impact the transcriptome. However, it is not known whether all the effects exerted by caspase-1 on transcriptome are mediated only by its well-known substrates. Therefore, we hypothesized that the effects of caspase-1 on transcriptome may be partially independent from IL-1β, IL-18, and Sirt-1.

**Methods:**

To determine new global and tissue-specific gene regulatory effects of caspase-1, we took novel microarray data analysis approaches including Venn analysis, cooperation analysis, and meta-analysis methods. We used these statistical methods to integrate different microarray datasets conducted on different caspase-1 knockout tissues and datasets where caspase-1 downstream targets were manipulated.

**Results:**

We made the following important findings: (1) Caspase-1 exerts its regulatory effects on the majority of genes in a tissue-specific manner; (2) Caspase-1 regulatory genes partially cooperates with genes regulated by sirtuin-1 during organ injury and inflammation in adipose tissue but not in the liver; (3) Caspase-1 cooperates with IL-1β in regulating less than half of the genes involved in cardiovascular disease, organismal injury, and cancer in mouse liver; (4) The meta-analysis identifies 40 caspase-1 globally regulated genes across tissues, suggesting that caspase-1 globally regulates many novel pathways; and (5) The meta-analysis identified new cooperatively and non-cooperatively regulated genes in caspase-1, IL-1β, IL-18, and Sirt-1 pathways.

**Conclusions:**

Our findings suggest that caspase-1 regulates many new signaling pathways potentially via its known substrates and also via transcription factors and other proteins that are yet to be identified.

**Electronic supplementary material:**

The online version of this article (doi:10.1186/s13045-017-0406-2) contains supplementary material, which is available to authorized users.

## Background

Caspase-1, a member of the cysteinyl aspartate-specific protease caspase family, is present in the cytosol as pro-caspase-1, an inactive zymogen, and requires the assembly of cytosolic multi-protein complexes known as “inflammasome” for proteolytic activation [1]. These complexes are assembled intracellularly in response to damage/danger signal-associated molecular patterns (DAMPs) and pathogen-associated molecular patterns (PAMPs), therefore, plays a similar role to that of Toll-like receptors (TLRs) at the cell surface [2]. Activated caspase-1 is required for cleaving/processing of pro-interleukin-1β (pro-IL-1β) and pro-IL-18 into mature pro-inflammatory cytokines IL-1β and IL-18, respectively. Moreover, activated caspase-1 can induce other inflammatory pathways by degrading anti-inflammatory sirtuin-1 (Sirt-1), which is a nicotinamide adenine dinucleotide (NAD)-dependent protein/class III histone deacetylase [3]. Caspase-1 has been shown to induce cell necrosis, pyroptosis, or pyrop-apoptosis [4] and exerts functions in various developmental stages. All these biological activities of caspase-1 occur at post-translational level (proteolytic processing). However, it remains unclear whether caspase-1 regulates gene transcription independent of the three well-characterized caspase-1 substrates IL-1β, IL-18, and Sirt-1.

The regulatory effects of caspase-1 on gene expression have been reported in the intestine [5], liver [5], and adipose tissue [6]. TLRs were shown to work in synergy with cytosolic-sensing receptor families including NLRs (NOD (nucleotide binding and oligomerization domain)-like receptors) in recognizing endogenous DAMPs and in mediating upregulation and activation of a range of inflammatory genes [7]. We recently reported a series of significant findings on the expression and roles of caspase-1 in vascular inflammation: (1) NLR, inflammasome components, and caspases are differentially expressed in human and mouse tissues [8]; (2) caspase-1 recognizes extended cleavage sites on its natural substrates [9]; (3) early hyperlipidemia promotes endothelial activation/dysfunction [10] via a caspase-1-sirtuin 1 pathway [11]; (4) inhibition of caspase-1 activation improves angiogenesis [12]; (5) caspase-1 mediates hyperlipidemia-weakened progenitor cell [13] vessel repair [14]; (6) caspase-1 mediates chronic kidney disease-promoted neointima hyperplasia of the carotid artery [15]; and (7) hyperhomocysteinemia induces caspase-1-mediated pyrop-apoptosis [4]. However, it remains unknown whether caspase-1 regulates gene expression in the aorta, which creates a knowledge gap for characterizing the role played by caspase-1 in promoting atherogenesis and arterial inflammation.

As we reported previously [8], TLRs and NLRs in tissues are differentially expressed and based on this observation, we classified the analyzed tissues in to three tiers. We categorized tissues such as the brain, lymph nodes, and thymus in to tier 1 because they constitutively express inflammasome components indicating their ability to assemble active inflammasomes rapidly in response to PAMPs/ DAMPs. Vascular tissue and heart was categorized in to tier 2 and 3, respectively. Tissues in tier 2 require upregulation of one inflammasome component, and tier 3 tissues require upregulation of two inflammasome components to assemble active inflammasomes. This suggests that unlike tissues in tier 1, tissues categorized in to tiers 2 and 3 show a delayed caspase-1 activation in response to PAMPs/ DAMPs. Therefore, we coined the term “inflammation privilege” for tissues that require “priming” to assemble active inflammasomes in response to stress. We emphasize that this delayed caspase-1 activation is a protective mechanism that increases the threshold for response to mitigate over-sensitivity and excessive tissue destruction by inflammasome/ IL-1β based innate immunity response.

One of our previous publications implicated that [9], in addition to regulatory effects executed by well-characterized caspase-1 substrates IL-1β, IL-18, and Sirt-1, caspase-1 (enzyme ID: EC 3.4.22.36) also cleaves many other protein substrates including those listed in the Brenda enzyme [16], and deneddylase involved in Epstein-Barr virus replication [17], transcription factors GATA4 [18], and PPARγ [19]. Since most proteins among 25 identified caspase-1 substrates are differentially expressed in tissues [9, 17], it remains unknown whether regulatory effects exerted by caspase-1 on gene expression via cleaving the gene regulatory substrates, including transcription factors [9], are tissue-specific. A recent report showed that NLRP3, a well-characterized NLR, is a transcription regulator of type 2T helper cell (Th2) differentiation [20] indicating that inflammasome components also can regulate the gene expression. Moreover, it was reported that caspase-1 can be activated in the nucleus [21, 22], suggesting the possibility that caspase-1 may directly regulate gene expression via an unknown mechanism. However, direct gene regulatory effects of caspase-1 remain unidentified.

Traditionally, meta-analysis refers to a statistical method that focuses upon contrasting and integrating the findings from the reports by different teams in order to identify shared patterns whereas these patterns could not be easily identified with each individual dataset. Meta-analyses have been widely used in clinical and epidemiological studies [23]. Recently, meta-analysis approach had also been applied to analyses of gene expression microarray data [24]. However, this approach has never been used to integrate different caspase-1 microarray datasets and also to identify novel targets which caspase-1 may regulate independent of IL-1β-, IL-18-, and Sirt-1.

In this report, we hypothesized that caspase-1 regulates the transcriptomes in a tissue-specific manner, and that these transcriptional effects are at least partially independent from the effects of IL-1β-, IL-18-, and Sirt-1. To test these hypotheses, we utilized Venn analysis and meta-analysis to compare our hyperlipidemic apolipoprotein E (ApoE−/− or ApoE KO) and caspase-1-deficient mouse aorta microarray data with caspase-1−/− intestine [5], caspase-1−/− liver [5], and caspase-1−/− adipose tissue [6] datasets deposited in the NIH-National Center for Biotechnology Information (NCBI)-Gene Expression Omnibus (GEO) database. This approach enabled us to determine the global and tissue-specific effects of caspase-1 on transcriptomes. Furthermore, we meta-analyzed the global transcriptional effects of IL-1β, IL-18, and Sirt-1 and used scatter plots to detect genes that are modulated independently or dependently (which we termed as cooperation analysis) to each other. Our results have demonstrated for the first time that caspase-1 regulates transcriptomes in both tissue-specific and global manner. Most strikingly, caspase-1 can regulate gene expression independently from IL-1β-, IL-18-, or Sirt-1-regulated pathways globally. In contrast, the caspase-1 function in regulating gene expression is well cooperated with Sirt-1 pathway in the adipose tissue. The findings will eventually lead to future development of novel therapeutics for inflammatory diseases.

## Methods

### Search for the microarray datasets

A thorough search was conducted to identify all the candidate datasets in the Gene Expression Omnibus databases from the NIH-National Center for Biotechnology Information and in the ArrayExpress databases from the European Molecular Biology Laboratory (EMBL)—European Bioinformatics Institute (EBI). Search terms included caspase1 (or Casp1), interleukin-1 beta (or IL-1β, IL-1βeta, IL-1β), interleukin-18 (or IL-18), and sirtuin-1 (or Sirt-1). Our meta-analysis were performed according to the “Preferred Reporting Items for Systematic Reviews and Meta-Analyses” guidelines (PRISMA) [25]. The flow diagram is shown in Additional file 1: Figure S1b. The details of the datasets that were analyzed are shown in Additional file 1: Table S1. We applied the following inclusion criteria for our study as shown in Additional file 1: Figure S1b: (1) the organism was *Mus musculus*, (2) the datasets were the results from RNA microarray assay experiments, (3) the datasets were derived from the experiments using transgenic (Tg, gene overexpression) or gene-deficient mouse samples (gene downregulation and deficiency) of Casp1, IL-1β, IL-18, or Sirt-1, and (4) the datasets were derived from in vitro studies with activation or inhibition of Casp1, IL-1β, IL-18, or Sirt-1. In addition, we applied the following exclusion criteria for our study: (1) the datasets were derived from microRNA array; (2) the datasets were derived from chromatin immunoprecipitation (ChIP) followed by microarray (ChIP-on-chip), and ChIP followed by DNA sequencing (ChIP-seq); (3) the datasets were derived from the experiments with sample size less than 2 (statistical parameter *p* value cannot be generated); and (4) specific reasons: (a) for example, the GEO dataset GSE26766 was derived from the experiments, where IL-1β was injected in intracerebroventricular (ICV) whereas the RNAs of the skeletal muscle were isolated to run the expression array and (b) the GEO dataset GSE16104 was derived from an experiment where IL-1 was treated on IL-1 receptor knockout mice. The gene expression regulatory effect of IL-1 was complexed by IL-1 receptor knockout effects in regulating gene expressions.

### Analysis of microarray data

Our gene expression data from microarray experiments with the aorta from five male ApoE^−/−^ and five male Caspase-1^−/−^ApoE^−/−^ mice were analyzed in the R statistical environment [26] using “oligo” and “limma” packages [27, 28] and were deposited in the NIH-GEO database (GSE72248). Briefly, total RNA was extracted from the aortas of mice using the RNeasy Kit (Qiagen, Valencia, CA). Samples were then labeled and hybridized to Affymetrix Genechip Mouse Gene 2.0.ST Arrays (Santa Clara, CA) according to the manufacturer’s instructions. Scanned microarray images were analyzed using the Affymetrix Gene Expression Console with Robust Multi-array Average normalization algorithm.

### Meta-analysis of gene expression across tissues

To make data extracted from different microarray datasets comparable, the analysis was conducted on raw data in R statistical environment (Additional file 1: Figure S1C-1, Table S1). Differential expression analysis of the experimental group versus the control group in each study was conducted using scripts modified from GEO2R [29]. Briefly, a design matrix was set up containing the samples that were to be compared. Differential expression was tested by lmFit and eBayes function from “limma” package [28]. *P* value was adjusted by the Benjamini-Hochberg method [30]. For each gene in each study, we then calculated the effect size using the effectsize function from “metaMA” package [31]. The unbiased effect size and its variance were combined across multiple studies using the directEScombi function. *P* value was calculated from the combined statistics using normal distribution and adjusted by Benjamini-Hochberg method [30]. To calculate the combined fold change for each gene, we meta-analyzed the fold change across studies by inverse variance weighting method using metagen function from “meta” package [32].

Venn analysis was performed in R statistical environment using the package “Venn Diagram” to analyze Casp1-regulated gene expression among different tissues (Additional file 1: Figure S1C-2). Heat maps and scatter plots were made using statistical tools provided by the R and Bioconductor projects. Statistical significance in this study was set at *p* < 0.05. Meta-analysis was used to directly compare datasets derived from different microarray datasets. FC/FC ((FC) fold change) scatter plots were used to determine whether caspase−/−-regulated genes could cooperate with genes regulated by IL-1β, IL-18, or Sirt1 as was previously described (from herein referred as cooperation analysis) [33] (Additional file 1: Figure S1C-3). For microarray datasets extracted from caspase-1−/− liver and adipose tissue, direct cooperation analyses without the precedent meta-analyses were performed to determine whether the caspase-1 regulatory genes cooperate with IL-1β, IL-18, and Sirt-1 regulatory genes (Additional file 1: Figure S1C-4).

## Results

### Caspase-1 regulates the majority of genes in a tissue-specific manner

We and others have previously shown that caspase-1 mediated activation of TLR and NLR genes and caspase-1 enzymatic substrate genes are differentially expressed in tissues [8]. Thus, we hypothesized that caspase-1 regulates gene expression in a tissue-specific manner. To test this hypothesis, we compared our mouse caspase−/− aorta microarray dataset with other reported three caspase-1−/− microarray datasets (both caspase-1−/− mice versus wild-type (WT) mice) extracted from the adipose tissues, liver, and intestine (aorta/GSE72248, adipose/GSE25205, liver/GSE32515, and intestinal/GSE32515). For analyzing the roles of caspase-1 in different parts of the mouse intestine, we collected microarray data from different sections of the intestine including the duodenum, jejunum, and ileum. Although these microarray datasets were obtained using different microarray platforms from different tissues or cells, since we used the raw data and conducted the same analysis as we did with our microarray data, all the data were statistically comparable. The rationale to focus on the caspase-1 function in the aorta was because our recent reports showed that caspase-1 promotes vascular inflammation and atherosclerosis in the aorta [8, 9, 11, 12, 14]. After summarizing all four normalized datasets, we found that expression of 11,039 genes were significantly changed among 29,753 mapped genes (37.1%) (Additional file 1: Table S1). Our results showed that the percentages of changes in gene expression in tissues ranged from 10.7 to 29.3%, with intestinal 11.1%, liver 13.5%, adipose 29.3%, and aorta 10.7%.

**Fig. 1:**
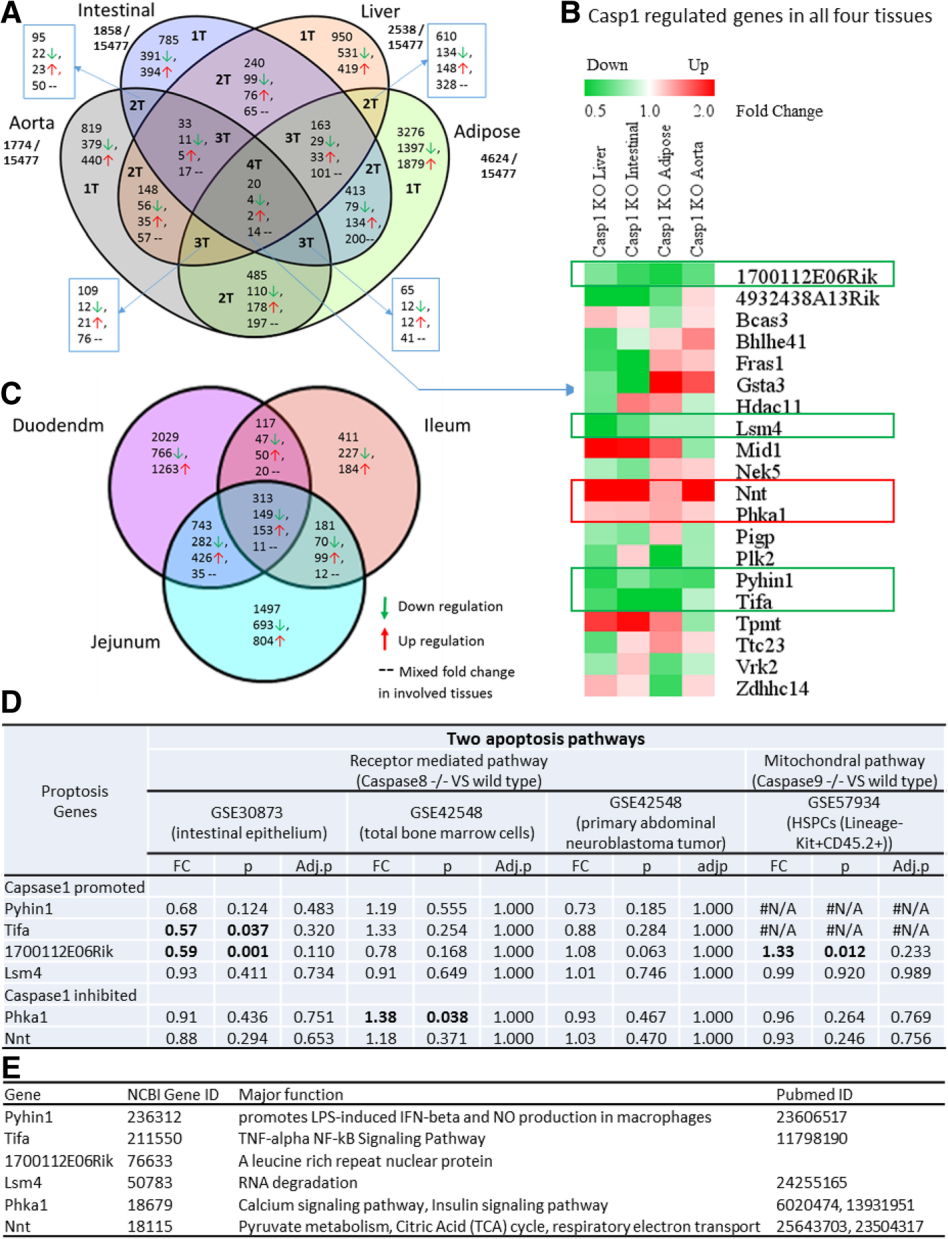
The Venn analysis show that caspase-1 gene regulatory effects have strong tissue specificities (direct comparison, non-meta-analysis). **a** Venn Diagram analysis of significantly changed genes among four different tissues comparing caspase-1 deletion to wild type. The logical relationships of caspase-1-regulated gene among four different tissues are shown in this diagram. 1Ts represent specific regulated genes in a single tissue (*green arrow* represent downregulated and *red arrow* represent upregulated), and 2Ts/3Ts/4Ts represent regulated genes in/among two/three/four involved tissues (−− represent mixed fold changes in involved tissues). **b** Heat map for fold changes of overlapped genes among four tissues. 1700112E06Rik, Lsm4, Pyhin1, and Tifa were all significantly downregulated by deletion of caspase-1 among four tissues. Nnt and Phka1 were significantly upregulated among the four tissues. And the other 14 genes were either upregulated or downregulated in analyzed tissues. **c** Venn diagram of significantly changed genes among three different intestinal tissues comparing caspase-1 deletion to WT. **d** Caspase-1 globally regulated gene expression are not ubiquitously regulated by two apoptosis pathways. **e** The major function of the six genes that are regulated by caspase-1 across all the four tissue types

The results generated primarily from microarray data analysis are lists of genes that are differentially expressed between different samples utilized for the study. Once such list is created, identifying the underlying biological themes and relationships of the altered genes are difficult without graphical representation. This task becomes more tedious specifically in a situation where multiple datasets generated from different microarray datasets have to be compared. Therefore, in such situations, Venn analysis/diagram comes as a very useful analytical tool as it helps to clearly visualize the genes that are shared between the different treatment groups analyzed. Therefore, we conducted Venn analysis on 15,477 genes that were shared among these 4 datasets to identify the common genes that are altered among different tissues (Fig. 1a).

Interestingly, we found that caspase-1 regulates gene expression differentially in tissues examined. In the aorta, caspase-1 regulates the expression of 1774 genes, including (i) 819 genes with the expression changed in the aorta alone, (ii) 95 genes shared with the intestine, (iii) 148 genes shared with the liver, (iv) 485 genes shared with the adipose tissue, (v) 109 genes shared with the liver and adipose tissue, (vi) 33 genes shared with the liver and intestine, (vii) 65 genes shared with the intestine and adipose tissue, and finally, (viii) 20 genes shared with the intestine, liver, and adipose tissues. The 20 genes that were overlapped/shared among all these 4 tissues suggest that these genes were regulated by caspase-1 independent of tissue-specific signaling and different transcriptomic environments. Figure 1b shows the heat map of the fold changes of these 20 overlapped genes. Of note, 14 genes regulated by caspase-1 were either upregulated or downregulated depending on the tissues, suggesting that the caspase-1 regulatory signals on those 14 genes are overpowered by the tissue differentiation signals.

Moreover, we conducted Venn analysis on all the caspase-1-regulated genes in three different types of intestinal sections (the duodenum, jejunum, and ileum) (Fig. 1c). As these three types of intestinal tissues are similar in histology, we found mostly similar effects of caspase-1 on gene regulation in these tissues. Among 5291, a total of 313 common genes were modulated by caspase-1 in all the three tissue types. Specifically, caspase-1 downregulated 149 genes and upregulated 153 genes in all the three intestinal sections. These results further confirm that caspase-1 play a tissue-specific role in gene regulation in the three types of intestinal tissues. Only 5.9% of genes were regulated by caspase-1 in a non-tissue-specific manner.

### Caspase-1 promotes global expression of four inflammatory genes and inhibits two genes involved in mitochondrial energy metabolism

In addition to serving as the essential regulator of inflammatory cell death (pyroptosis), the deficiencies of either caspase-1 or inflammasome components such as NLRP3 and apoptosis-associated speck-like protein containing a caspase-recruitment domain (ASC) protected from high fat diet-induced insulin resistance, glucose intolerance, obesity [34], triglyceride metabolism [35], mitochondrial autophagy [36], and metabolic inflammation [37]. However, the mechanisms underlying how caspase-1 regulates these pathways related to metabolism remain unclear.

Our Venn analysis revealed that caspase-1 regulate 20 genes globally, and out of these 20 genes, only 6 genes showed the same direction of change: 4 genes including Lsm family of RNA-binding protein 4 (Lsm4) [38], cell differentiation regulator 1700112E06Rik (C10orf11, a nuclear protein, containing a leucine-rich repeat-containing domain and 4 leucine-rich repeats), tumor necrosis factor (TNF) receptor-associated factor (TRAF)-interacting protein with forkhead-associated domain (Tifa—an essential innate immunity regulator [39] and IL-1 signaling regulator [40]), and pyrin and HIN domain family member 1 (pyhin1—a newly identified asthma susceptibility locus [41] and a HIN-200 family of interferon-inducible proteins member) were significantly decreased in all the four tissues of caspase-1 KO mice, suggesting that caspase-1 activation promotes the activation of these four genes. In addition, caspase-1 deficiency in four tissues upregulated the two following genes: (1) nicotinamide nucleotide transhydrogenase (Nnt) [42], which acts as a regulator of nicotinate/nicotinamide metabolism, NADPH-generating enzyme, and mitochondrial membrane potential keeper [43] and (2) phosphorylase kinase α1 (Phka1) [44], which regulates calcium signaling, insulin signaling, and also acts as a myopathy inhibitor [45]. Therefore, this result particularly indicates that caspase-1 activation inhibits the expression of these two genes in the four tissues examined (Fig. 1b).

While caspase-1 is an essential regulator for inflammatory cell death (pyroptosis), caspase-8 and caspase-9 are necessary components for cell death receptor-mediated apoptosis pathway and mitochondria-mediated apoptosis pathway, respectively [46, 47]. However, the downstream genes and pathways regulated by various caspases remain poorly characterized. We hypothesized that the aforementioned six caspase-1 globally regulated genes may be the hallmark genes of caspase-1-specific pathways, independent of other apoptosis regulators such as caspase-8 and caspase-9. To test this hypothesis, we cross-examined the expression of the six caspase-1 globally regulated genes in caspase-8 KO caspase-9 KO tissues. As shown in Fig. 1d, among four caspase-1-promoted genes (downregulated in caspase-1 KO mice), caspase-8 also promoted the expression of two genes including Tifa and 1700112E06Rik in one of three tissues (intestinal epithelium) (GSE30873). In contrast, caspase-9 KO upregulated 1700112E06Rik in the lineage-Kit+CD45.2+hematopoietic cells (GSE57934). Among the two caspase-1-inhibited genes (upregulated in caspase-1 KO mice), caspase-8 inhibited phka1 only in the bone marrow cells. Taken together, our Venn analysis results suggested that the six caspase-1 globally regulated genes are not generally modulated by caspase-8-dependent death receptor apoptosis pathway or by caspase-9-dependent mitochondria-mediated apoptosis pathway. Therefore, it is justified that the six genes regulated globally by caspase-1 can be considered as specific hallmark genes that indicate caspase-1 activation.

By analyzing the major functions of the six caspase-1 globally regulated genes (Fig. 1e), we suggest several new pathways where caspase-1 may (1) promote interferon-β (IFN-β)- and NF-kB-mediated inflammatory pathways, (2) regulate new RNA degradation pathway, and (3) inhibit insulin signaling and mitochondrial energy metabolism pathway, which are well correlated with the reported caspase-1 role in mitochondrial disassembly, mitochondrial reactive oxygen species generation, dissipation of mitochondrial membrane potential, and inhibition of mitophagy etc. [48].

### Genes regulated by caspase-1 partially cooperate with genes regulated by Sirt-1 during organ injury and inflammation in the adipose tissue but not in the liver

Caspase-1 regulates non-inflammatory lipid/energy metabolism including increasing serum triglyceride levels [35, 37], decreasing mitochondrial respiration and protecting hepatocyte death [36], promoting adipocyte differentiation, and enhancing insulin resistance [49], which may not all be dependent on the effects of caspase-1 on histone deacetylase Sirt-1. Therefore, we hypothesized that the non-inflammatory effects of caspase-1 are partially independent from that of Sirt-1 in mouse adipose tissue. Since we found that caspase-1 had tissue-specific regulatory effects on gene expression, we examined caspase-1-mediated gene expression in the adipose tissue in detail.

**Fig. 2:**
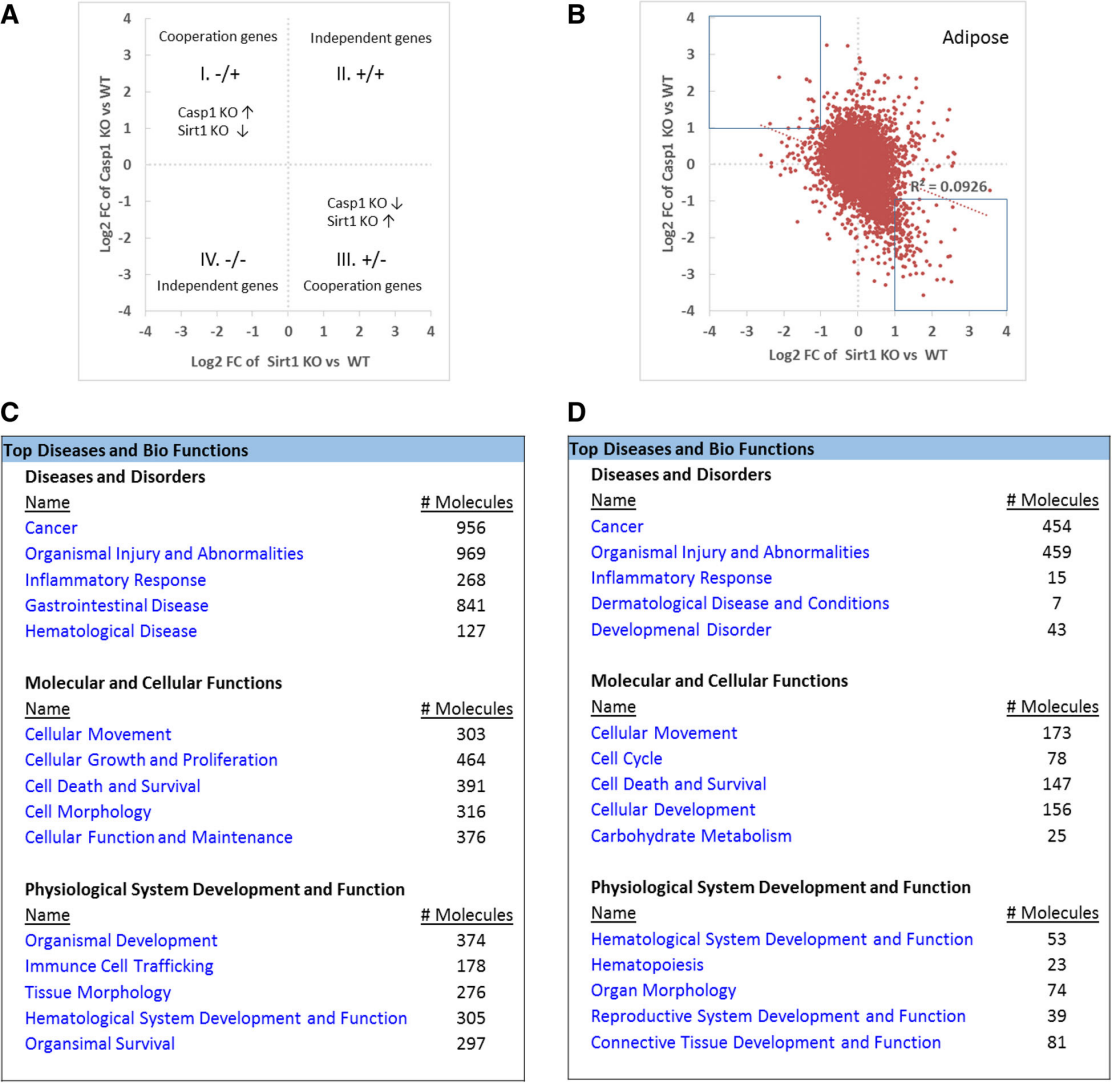
Cooperation analysis shows that among 163 genes that are highly regulated by caspase-1 and Sirt-1 in mouse adipose tissue, caspase-1 and Sirt-1 cooperatively regulates 142 genes (87.1%). **a** Explanation for the FC/FC plot. Plots distributed in II and IV quadrants represent caspase-1 independently regulated genes from Sirt-1. **b** Cooperation of Sirt-1 and caspase-1 in the adipose tissue. FC/FC plot comparing Log2 FC of genes for Sirt-1 KO vs. WT (*x-axis*) and parallel caspase-1 KO vs. WT (*y-axis*). Genes with significant expression changes (with fold changes no less than 2) were gated. Detailed information about these genes was shown in Additional file 1: Table S5. **c** The top five signatures of cooperation genes (*p* < 0.05) reversely regulated by caspase-1 and Sirt-1 in the adipose tissue. **d** The top five signatures of non-cooperation genes (*p* < 0.05) in the adipose tissue

In order to validate this hypothesis, we extracted original data from two microarray datasets conducted on caspase-1 and Sirt-1-deficient mouse adipose tissues (GSE25205 and GSE30247) (Additional file 1: Table S2). Then we applied cooperation analysis (explained in the Methods section) to detect the correlation between caspase-1 regulatory genes on the same set of genes regulated by Sirt-1 in the adipose tissue. The fold-change differences in gene expression between caspase-1 KO and Sirt-1 KO were shown by the Log2 (fold-change, FC)/Log2 (fold-change, FC) plots using the method previously reported [50]. Cooperation analysis enables us to graphically visualize the genes that might be altered due to direct effect of caspase-1 on Sirt-1 (Fig. 2a). Based on the fact that active caspase-1 degrades Sirt-1, we reasoned that if caspase-1 fully uses inhibition of Sirt-1 to regulate gene expression, the genes regulated by caspase-1 should cooperate with the genes regulated by Sirt-1, therefore, caspase-1 should be concentrated in the first quadrant [(I) with the genes increased in caspase-1 KO (caspase-1-inhibited genes) and the genes decreased in Sirt-1 KO (Sirt-1-promoted genes)] and in the third (III) quadrant [genes decreased in Casp1 KO (caspase-1-promoted genes) and the genes increased Sirt-1-deficient adipose tissues (Sirt-1-inhibited genes)] (Fig. 2a). Therefore, the expression of genes that fall in to quadrant I and III can be altered due to the cooperation between caspase-1 and Sirt-1; thus, we termed these genes as “cooperation genes”. The genes that do not show a direct link between caspase-1 and Sirt-1 fall in to quadrant II and IV and named as “independent genes”. This method had been used in a previous publication to visualize the cooperation of two different proteins on gene regulation [33].

**Fig. 3:**
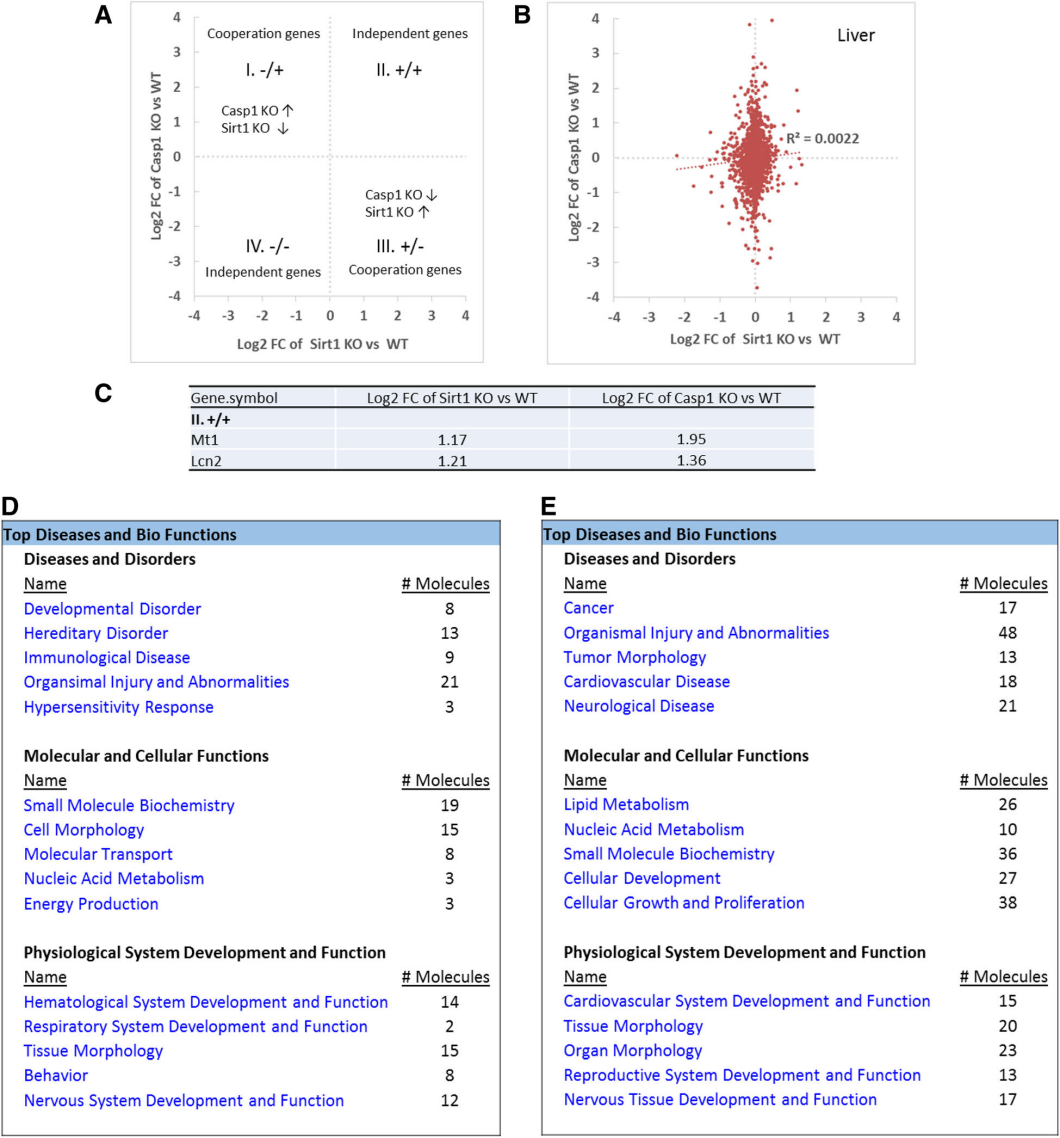
Caspase-1 does not cooperate with Sirt-1 in regulating genes in mouse liver. **a** Explanation for the FC/FC plot. Plots distributed in quadrants II and IV represent caspase-1 independently regulated genes from Sirt-1. **b** Cooperation of Sirt-1 and caspase-1 in the liver. FC/FC plot comparing Log2 FC of genes for Sirt-1 KO vs. WT (*x-axis*) and parallel caspase-1 KO vs. WT (*y-axis*). **c** Significantly changed genes (fold change > 2) regulated by caspase-1 and Sirt-1 deficiency in mouse liver. **d** The top five signatures of genes regulated by caspase-1 in cooperation with Sirt-1 in the liver. **e** The top five signatures of genes that are regulated by caspase-1 non-cooperatively with Sirt-1 (*p* < 0.05) in liver

To our surprise, the expressions of most transcripts regulated in caspase-1 KO and Sirt-1 KO were non-evenly distributed in the quadrants I and III (Fig. 3b, *R* ^2^ = 0.0926). The results suggest that the gene transcription regulatory effects of caspase-1 and Sirt-1 pathways are collaborated very well, emphasizing that most of the non-inflammatory effects regulated by caspase-1 is mediated via Sirt-1 in the mouse adipose tissue.

Caspase-1 and Sirt-1 cooperatively changed the expression of 142 genes significantly (≥2 folds) (Fig. 2b). Further, caspase-1 deficiency upregulated only 15 genes (quadrant I) and downregulated 127 genes (quadrant III), which were all inversely regulated by Sirt-1 deficiency in the adipose tissue (Additional file 1: Table S5). In addition, 17 genes uncooperatively changed (both Sirt-1 KO-promoted and caspase-1 KO-promoted) which is around 10% of all the 163 significantly changed genes in the cooperative study of caspase-1 KO and Sirt-1 KO in the adipose tissue. Moreover, in the quadrant IV, caspase-1 KO decreased the expression of four genes including Vnn1 (vanin 1, glycosylphosphatidylinositol (GPI) anchor binding, and pantetheine hydrolase activity), Plau (plasminogen activator, serine-type endopeptidase activity), Mal (T cell differentiation protein, lipid binding, and structural constituent of myelin sheath), and Sprr1a (small proline-rich protein 1A, structural molecule activity *and* protein binding, bridging), which were also inhibited by Sirt-1 KO, suggesting that caspase-1 independently promotes the expression of these four genes.

Our further studies using Ingenuity Pathway Analysis software revealed that caspase-1 has a profound effect on cellular growth, proliferation, death, and survival via Sirt-1-dependent and Sirt-1-independent pathways in the mouse adipose tissues (Fig. 2c, d). Additionally, our analysis implicated that caspase-1-mediated regulation of cellular functions and maintenance is significantly cooperated with Sirt-1 pathway in the adipose tissue. Furthermore, our pathway analysis indicates that caspase-1 can regulate carbohydrate metabolism in the adipose tissue most likely through an independent pathway not related to Sirt-1.

Our Venn analysis clearly demonstrated that the expression of the majority of genes regulated by caspase-1 are tissue-specific. Therefore, we hypothesized that caspase-1 and Sirt-1 cooperation in gene regulation is tissue-specific. To test the hypothesis, we applied the cooperation analysis of the transcriptional regulatory effects of caspase-1 with the effects of Sirt-1 on the same set of genes in the liver (Fig. 3a, b). We extracted the original data from two microarray datasets conducted on caspase-1- and Sirt-1-deficient (GSE32515 and GSE46895, respectively) mouse liver (Additional file 1: Table S1 and S2). The fold-change differences in genes differentially expressed in caspase-1 KO and Sirt-1 KO are shown by the Log2 (fold-change, FC)/Log2 (fold-change, FC) plots using the method reported [50].

In contrast to what we found in the adipose tissue, the expression of all the genes regulated in caspase-1 KO liver and Sirt-1 KO liver were evenly distributed in the four quadrants (*R* ^2^ = 0.0022) (Fig. 3b). As shown in Fig. 3c, we did not find any genes with high fold changes in the cooperative first quadrant (I) and third quadrant (III). We found only two genes significantly expressed in the second quadrant (II) including Mt1 (metallothionein 1A, related to copper ion binding and cadmium ion binding according to the report in the GeneCards database [51] and Lcn2 (lipocalin 2, related to protein homodimerization activity and iron ion binding according to the report in the GeneCards database). The expression of these two genes were increased in both Sirt-1 KO arrays and caspase-1 KO arrays, indicating that the two genes are inhibited by Sirt-1 and caspase-1 pathways. Since caspase-1 degrades Sirt-1, these results suggested that caspase-1 pathway and Sirt-1 pathway do not significantly cooperate in regulating gene expression in the liver. As there are no significant differences in the expression of caspase-1 and Sirt-1 in the liver and adipose tissue (the GeneCards database), thus the underlying mechanism that is involved in the tissue-specific cooperation between caspase-1 and Sirt-1 pathways remains unknown.

Further analyses with the Ingenuity Pathway Analysis for all the genes that changed significantly (*p* < 0.05) showed that caspase-1 and Sirt-1 cooperated in energy production and molecular transport in the liver (Fig. 3d). Also, our results indicated that caspase-1 and Sirt-1 can regulate the genes related to lipid and nucleic acid metabolism non-cooperatively (Fig. 3e).

### Caspase-1 cooperates with IL-1β in regulating less than half of the gene expression in mouse liver

**Fig. 4:**
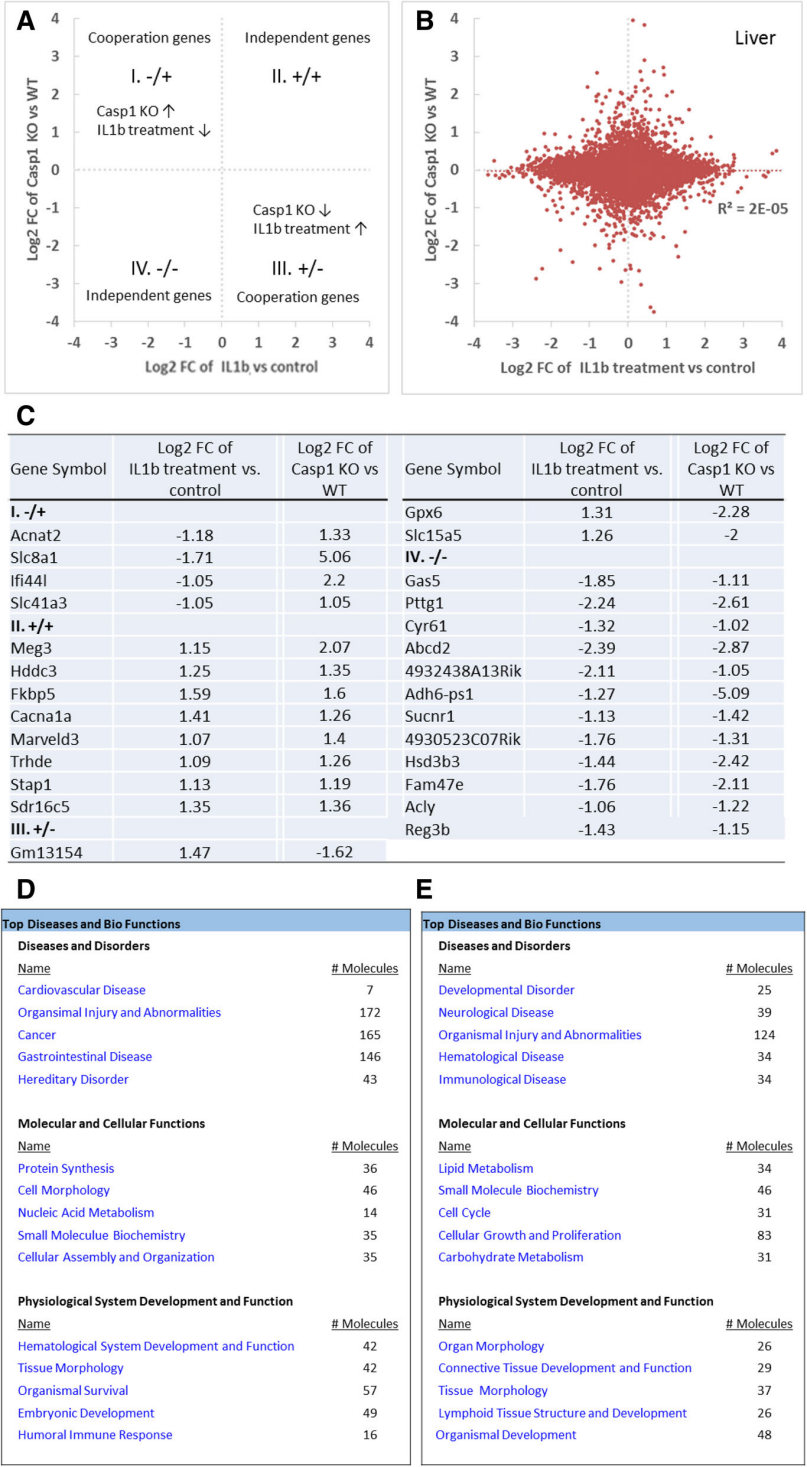
Cooperation analysis shows that among 27 highly regulated genes by caspase-1 and IL-1β in mouse liver, Caspase-1 and IL-1β cooperatively regulated 7 genes (25.9%). **a** Explanation for the FC/FC plot. Plots distributed in quadrants II and IV represent genes that are regulated by caspase-1 independently of IL-1β. **b** Cooperation of IL-1β and caspase-1 in the liver. FC/FC plot comparing Log2 FC of genes for IL-1β treatment vs. control (*x-axis*) and parallel caspase-1 KO vs. WT (*y-axis*). **c** Represents significantly changed genes (fold change > 2) regulated by caspase-1 and IL-1β in Fig. 5b. **d** and **e**. The top five signatures of genes that are regulated by caspase-1 in cooperation with IL-1β in mouse liver (**d)**. The top five signatures of the genes that are regulated by caspase-1 non-cooperatively with IL-1β (*p* < 0.05) in the liver

Similarly, based on the fact that active caspase-1 is responsible in generating mature IL-1β, we tested whether caspase-1 fully uses IL-1β in regulating the expression of the genes. For this analysis, we compared a microarray dataset conducted on caspase-1 KO vs. WT to a dataset derived from IL-1β treatment vs. non-treatment. The caspase-1 regulatory genes that cooperate well with IL-1β regulatory genes should be concentrated in the first (I) quadrant where the genes increased in caspase-1 KO (caspase-1-inhibited genes) and the genes decreased in IL-1β treatment (IL-1β-inhibited genes) are included. Also, the third (III) quadrant contain genes decreased in caspase-1 KO (caspase-1-promoted genes) and IL-1β treatment-induced genes indicate cooperation between caspase-1 and IL-1β (Fig. 4a).

Our analysis showed that the expression of all the genes regulated by caspase-1 deficiency and IL-1β treatment were evenly distributed in the four quadrants in Fig. 4b (*R* ^2^ = 2^E−05^). Among 27 significantly changed genes with fold changes higher than two in all four quadrants, caspase-1 and IL-1β significantly cooperated in regulating only seven genes in the quadrants I and III in mouse liver (Fig. 4c). Caspase-1 cooperated with IL-1β and inhibited the expressions of four genes Acnat2 (acyl-coenzyme A amino acid N-acyltransferase 2), Slc8a1 (Na^+^/Ca^2+^-exchange protein 1), Ifi44l (interferon-induced protein 44 like), and Slc41a3 (solute carrier family 41 member 3) in the liver (quadrant I). Also, caspase-1 cooperated with IL-1β in the liver (quadrant III) to promote the expression of three genes including Gm13154 (Zinc-finger double domain), Gpx6 (glutathione peroxidase 6), and Slc15a5 (solute carrier family 15 member 5). In addition, caspase-1 inhibited the expression of 8 genes independently from IL-1β including Meg3 (maternally expressed 3 (non-protein coding)), Hddc3 (HD domain containing 3), Fkbp5 (FK506 binding protein 5), Cacna1a (calcium channel, voltage-dependent, P/Q type, alpha 1A subunit), Marveld3 (MARVEL domain containing 3), Trhde (thyrotropin-releasing hormone degrading enzyme), Stap1 (signal-transducing adaptor family member 1provided), and Sdr16c5 (short-chain dehydrogenase/reductase family 16C) (quadrant II), and promoted 12 genes independently from IL-1β including Gas5 (growth arrest-specific 5 (non-protein coding)), Pttg1 (pituitary tumor-transforming 1), Cyr61 (cysteine-rich angiogenic inducer 61), Abcd2 (ATP-binding cassette, subfamily D (ALD), member 2), 4932438A13Rik (RIKEN cDNA 4932438A13 gene), Adh6-ps1 (alcohol dehydrogenase 6 (class V), pseudogene 1), Sucnr1 (succinate receptor 1), 4930523C07Rik (RIKEN cDNA 4930523C07), Hsd3b3 (hydroxy-delta-5-steroid dehydrogenase, 3 beta- and steroid delta-isomerase 3), Fam47e (family with sequence similarity 47 member E), Acly (ATP citrate lyase), and Reg3b (regenerating islet-derived 3 betaprovided) (quadrant IV) in the liver.

Further studies using the Ingenuity Pathway Analysis revealed that for all the genes changed significantly (*p* < 0.05), caspase-1 and IL-1β are cooperated in the top five signatures in regulating molecular and cellular functions (166 genes, 42.5%) such as protein synthesis, cell morphology, nucleic acid metabolism, small molecule biochemistry, and cellular assembly and organization (Fig. 4d). Figures 3e and 4e clearly suggest that the effects of caspase-1 on lipid metabolism, cellular growth, and cellular proliferation do not rely on either Sirt-1 or IL-1β pathways. Since the expression of all the genes regulated by caspase-1 deficiency and IL-1β treatment were evenly distributed in the four quadrants, our results suggest that the majority of gene regulatory effects of caspase-1 are independent from that of IL-1β regulatory pathways in mouse liver.

### The meta-analysis identifies 40 caspase-1 globally regulated genes, suggesting that caspase-1 regulates many novel pathways across tissues

We demonstrated above that the transcriptional effects of caspase-1 are tissue-specific. In addition, we found for the first time that the significant transcriptional effects of caspase-1 on genes are independent from that of IL-1β or Sirt-1 pathways in the liver, but are cooperated with that of Sirt-1 pathway in adipose tissue. Also as shown in Venn diagrams (Fig. 1a, c), the transcriptional effects of caspase-1 on one tissue can be shared with other tissue(s). Therefore, we hypothesized that more shared/global transcriptional effects of caspase-1 can be revealed by conducting a meta-analysis on the datasets.

**Fig. 5:**
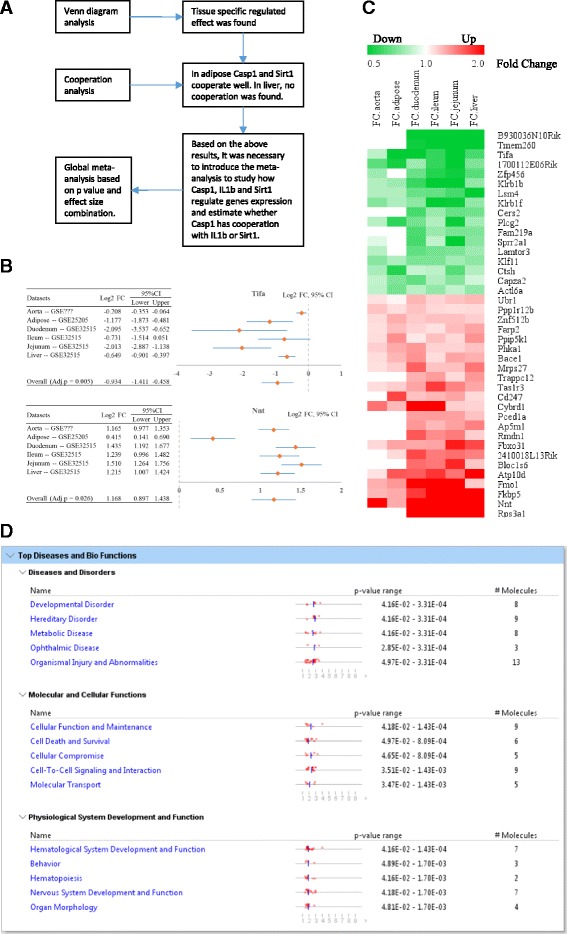
The meta-analysis identified 40 Caspase-1 globally regulated genes. **a** Logic flow of introducing meta-analysis. **b** Forest plot of two selected genes as examples. **c** Heat map for fold changes of significantly changed genes among six different tissues comparing caspase-1 deletion to WT. * Number form Gene database. **d** The top 5 signatures of 40 caspase-1 globally regulated genes (adj. *p* < 0.05)

To examine this hypothesis, we performed the meta-analysis on those data based on *p* value and effect size combination with the strategy shown in Fig. 5a. Meta-analysis is a tool that can effectively use to integrate independent but related datasets and enables to derive robust estimation than is possible from the measure attained from an individual analysis. Further, meta-analysis provides higher statistical power by increasing the sample size that allows to detect an overall effect and also may highlight subtle variations [52]. To explain how meta-analysis work, we used forest plots to illustrate how the expression of two selected genes changed across six microarrays conducted on different tissues (Fig. 5b).

The meta-analysis results showed that caspase-1 deficiency globally inhibits the expression of 17 genes (caspase-1-promoted genes) and promotes the expression of 23 genes (caspase-1-inhibited genes) (Fig. 5c). Of note, three out of four caspase-1 KO-inhibited genes we identified by Venn analysis was also confirmed by our meta-analysis. These genes are Tifa, 1700112E06Rik, and Lsm4. Similarly, the two caspase-1 KO-promoted genes, Phka1 and Nnt identified by Venn analysis were also found by the meta-analysis. The comparison between the results we obtained by meta-analysis and Venn analysis suggest that our meta-analysis was really more inclusive and reveal much broader global transcriptional effects of caspase-1 than the Venn analysis. Specifically, our meta-analysis enabled us to identify 40 global caspase-1-regulated genes while Venn analysis revealed only 6 genes with the statistical significance (adjusted *p* < 0.05), indicating that meta-analysis is more sensitive than the Venn analysis.

The pathway analysis (Fig. 5d) we conducted suggests that caspase-1 regulated 40 genes play significant roles in a variety of functions including cellular function and maintenance, cell death and survival, cellular compromise, cell-to-cell signaling and interaction, and molecular transport. Further, via these molecular pathways, caspase-1 may induce developmental disorder, hereditary disorder, metabolic disease, ophthalmic disease, and organismal injury and abnormalities.

The literature search conducted until February 2016 showed that the biological functions of three well-characterized caspase-1 substrates resulted in a large number of publications in PubMed, with 26,451 for IL-1β, 7563 for IL-18, and 3206 for Sirt-1. In contrast, 35 out of 40 caspase-1 globally regulated genes are not extensively studied and have less than 100 publications in the PubMed. In addition, 9 out of the 35 caspase-1 regulatory genes identified in meta-analysis are novel genes and no reports specifying their role had been published so far. There have been only few “remotely related” publications available on these genes listed in the NCBI-Gene database. To the best of our knowledge, we report for the first time the results obtained by meta-analysis conducted on caspase-1-related microarray data sets and have identified novel caspase-1 targeted genes and pathways.

### The meta-analysis identified new cooperatively and non-cooperatively regulated gene caspase-1, IL-1β, IL-18, and Sirt-1 pathways

We hypothesized that the global transcriptional effects of IL-1β in various tissues can be revealed by a meta-analysis. To examine this hypothesis, we performed a meta-analysis on those datasets based on *p* value and effect size combination with the strategy similar to that shown in Fig. 5a. The three IL-1β-related microarray datasets were collected from three different mouse tissues, two of them were IL-1β transgenic (Tg) samples (from the lung and stomach) compared to WT (GSE24931 [53] and GSE38075 [54]); and the other one had IL-1β treated samples (from liver) compared to control (GSE19272 [55]). In comparison to that in WT mice and untreated samples, the percentages of genes with significant expression changes were different in the range from 9.0 to 38.9% (IL-1β stimulation for 1 h (h) in the liver 9.0%, IL-1β stimulation for 4 h in the liver 10.8%, Tg-IL-1β in the lung 20.7%, and Tg-IL-1β in the stomach 38.9%). After the normalization of all the data, we found 14,650 significantly changed genes (40.0%) among 36,600 mapped genes. Total 12,285 overlapped genes were found among these datasets when intersected (Additional file 1: Table S2).

**Fig. 6:**
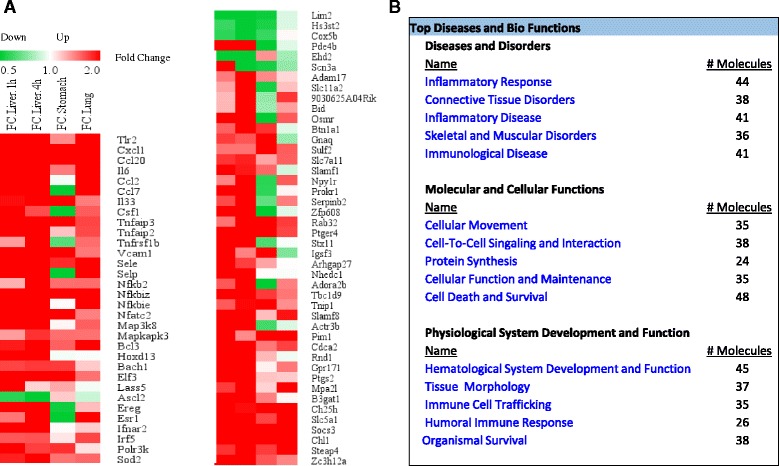
Meta-analysis of IL-1β microarray datasets identified 76 genes that are globally regulated by IL-1β (FC > 1.5). **a** Heat map for fold changes of significantly changed genes with adj. *p* < 0.05 and FC > 1.5 comparing treatment or transgenic of IL-1β to control. **b** The top five signatures of IL-1β significantly regulated genes (adj. *p* < 0.05, FC > 1.5)

We also analyzed the gene regulatory function of IL-1β among tissues such as the liver, stomach, and lung. In Fig. 6a and Additional file 1: Table S10, IL-1β inhibits the expression of seven genes including Lim2 (lens intrinsic membrane protein 2), Hs3st2 (heparan sulfate-glucosamine 3-sulfotransferase 2), Cox5b (cytochrome *c* oxidase subunit 5B), Pde4b (phosphodiesterase 4B), Ascl2 (achaete-scute family bHLH transcription factor 2), Ehd2 (EH domain-containing 2), and Scn3a (sodium voltage-gated channel alpha subunit 3), and promotes the expression of 69 genes (fold change >1.5, adjust *p* < 0.05). The pathway analysis (Fig. 6b) suggest that IL-1β globally regulated 76 genes play significant roles in various molecular/cellular functions including cellular movement, cell-to-cell signaling and interaction, protein synthesis, cellular function and maintenance, and cell death and survival, which may also contribute to the development of inflammatory response, connective tissue disorders, inflammatory disease, skeletal and muscular disorders, and immunological disease. To the best of our knowledge, a comprehensive meta-analysis had not been done on IL-1β-related microarray datasets deposited in the NCBI GeoProfile database before; therefore, the IL-1β-mediated signaling pathways we report here are novel.

**Fig. 7:**
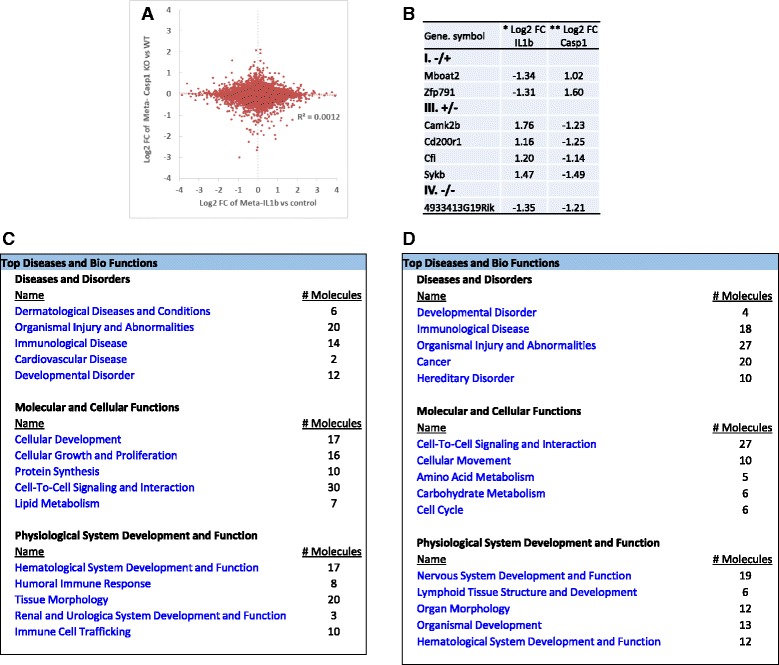
Cooperation analysis shows that among the 7 caspase-1 and IL-1β globally regulated genes with >2 fold change, caspase-1 and IL-1β cooperatively regulate 6 genes (85.7%). **a** FC/FC plot comparing log2 transformed fold changes (Log2 FC) of genes for meta-Caspase-1 (deletion of Caspase-1 among six different tissues) vs. wild type (*x-axis*) and parallel meta-IL-1β (meta-analysis of treatment and transgenic IL-1β) vs. control (*y-axis*). **b** Significantly changed genes with fold changes higher than two among the four quadrants. **c** Further analysis by Ingenuity Pathway revealed the top five signatures of the genes regulated by caspase-1 with cooperation of IL-1β. **d** The top five signatures of the genes that are regulated by caspase-1 non-cooperatively with IL-1β (*p* < 0.05)

Previously, we conducted cooperation analysis to determine the relationship between caspase-1 and IL-1β regulatory genes in mouse liver (Fig. 4). Following the same logic, we then applied the cooperation analysis to determine the global regulatory effects of caspase-1 with the effects of IL-1β on the same set of genes we identified through meta-analysis (Fig. 7a, b). The fold-change differences in gene expression between caspase-1 KO induced and IL-1β transgene/treatment induced were shown by the Log2 (fold-change, FC)/Log2 (fold-change, FC) plots using the method reported [50].

Similar to that in Fig. 4, the expression of all the genes regulated by caspase-1 KO and IL-1β treatment were evenly distributed in the four quadrants (Fig. 7a) (*R* ^2^ = 0.0012). In all the four quadrants, the expression of seven genes were significantly changed (fold change >2) by caspase-1 deficiency; and these genes behaved either cooperatively or uncooperatively with IL-1β treatment (Fig. 7b). Caspase-1 cooperated with IL-1β and inhibited the expression of two following genes: (1) Mboat2 (membrane bound O-acyltransferase domain containing 2, that converts lysophosphatidylethanolamine (lysoPE) to PE and lysophosphatidic acid (lysoPA) to PA) and (2) Zfp791 (zinc finger protein 791) (quadrant I). Further, caspase-1 cooperated with IL-1β (quadrant III) and promoted the expression of four genes including Camk2b (calcium/calmodulin-dependent protein kinase II beta), Cd200r1 (mediated signaling by GPCR and MHC class I-mediated antigen processing and presentation), Cfi (complement factor I, serine-type endopeptidase activity *and* scavenger receptor activity), and Syk (spleen tyrosine kinase). In addition, we also identified one IL-1β inhibited but caspase-1-promoted gene 4933413G19Rik (an uncharacterized protein-coding gene, NCBI database and GeneCards database). After detailed analysis for the integrated pathways with all the significantly changed genes (*p* < 0.05), we found that caspase-1 and IL-1β are globally cooperated in regulating cell development, cell growth and proliferation which may also explain the involvement of the two genes in protein synthesis and lipid metabolism pathways (Fig. 7c). Furthermore, both caspase-1 and IL-1β regulates cell to cell signaling and interactions via dependent and independent pathways (Fig. 7c and d). Our pathway analysis further indicates that caspase-1 and IL-1β, either cooperatively or uncooperatively exerts a profound effect on metabolism across tissues by regulating lipid metabolism, amino acid metabolism, and carbohydrate metabolism.

**Fig. 8:**
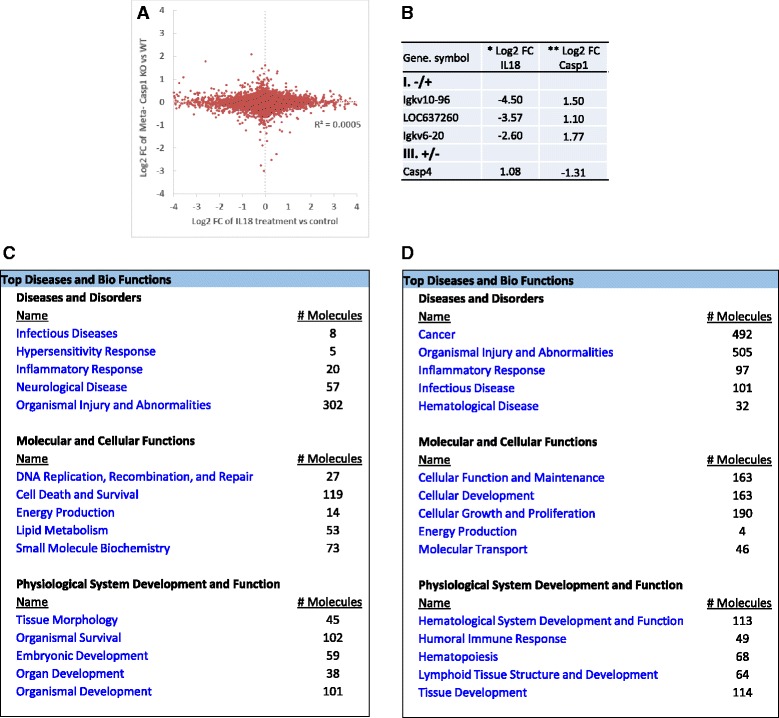
Cooperation analysis shows that caspase-1 and IL-18 cooperatively regulated all of the four genes that had significant expression changes. **a** FC/FC plot comparing Log2 FC of genes for meta-caspase-1 (deletion of caspase-1 among six different tissues) vs. wild type (*x-axis*) and parallel IL-18 stimulation vs. control (*y-axis*). **b** Significantly changed genes with fold changes higher than two among the four quadrants. **c**/**d** The top five signatures of genes that are regulated by caspase-1 cooperatively with IL-18 and non-cooperatively (*p* < 0.05)

**Fig. 9:**
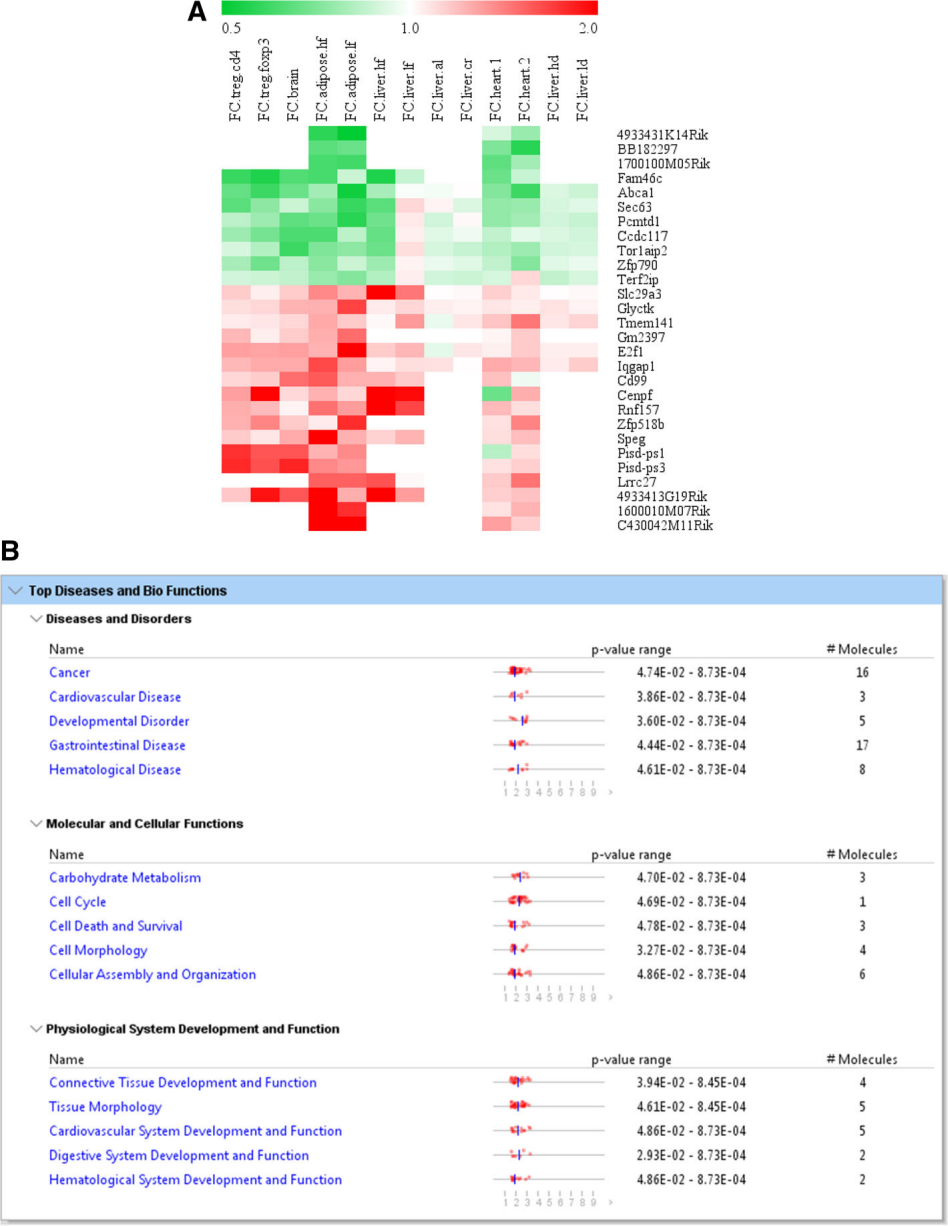
The meta-analysis of Sirt-1 microarray data identified 28 Sirt-1 globally regulated genes. **a** Heat map for fold changes of significantly changed genes with adj. *p* < 0.05 comparing Sirt1 KO to WT or comparing control to treatment or transgenic of Sirt-1. **b** The top five signatures of Sirt-1 globally regulated genes as revealed by Ingenuity Pathway Analysis (adj. *p* < 0.05)

Similarly, we studied the cooperation between caspase-1 and IL-18 regulatory genes. For this analysis, we utilized the only available dataset on IL-18 (GSE33627), which was conducted on murine natural killer cells. Among 20,456 mapped genes, 9519 of them (46.5%) were found to be significantly changed in this microarray dataset (Additional file 1: Table S2) [56]. Since there are no caspase-1 datasets done on murine natural killer cells, we then applied the cooperation analysis to determine the meta-analysis-generated global caspase-1 regulatory genes with the IL-18 regulatory genes in murine natural killer cells (Fig. 8a and b). The fold-change differences in gene expression between caspase-1 KO induced and IL-18 treatment induced were shown by the Log2 (fold-change, FC)/Log2 (fold-change, FC) plots using the method reported [50]. Similar to that in Fig. 4, the expression of all the genes regulated by caspase-1 KO and IL-18 treatment were evenly distributed in the four quadrants (Fig. 9a) (*R* ^2^ = 0.0005). In all four quadrants, expression of 4 genes were significantly modulated (fold changes >2) by caspase-1 deficiency. The changes of these genes were either cooperative or uncooperative with IL-18 treatment (Fig. 8b). Caspase-1 cooperated with IL-18 and inhibited the expression of three immunoglobulin variable genes Igkv10-96 (immunoglobulin kappa variable 10–96), LOC637260 (Ig kappa chain V-IV region B17 precursor-like), and Igkv6-20 (immunoglobulin kappa variable 6–20) (quadrant I). Furthermore, caspase-1 cooperated with IL-18 (quadrant III) and promoted the expression of one gene, which is caspase-4 (murine homolog caspase-11). Of note, a recent report showed that non-canonical inflammasome activation of caspase-4/caspase-11 also reciprocally governs the activation of IL-18 [57].

Interestingly, after detailed analysis for the integrated pathways with all the significantly changed genes (*p* < 0.05), we found that caspase-1 and IL-18 globally cooperate to regulate lipid metabolism similar to our finding with caspase-1 and IL-1β (Fig. 8c). This indicates that caspase-1 may have a profound effect on lipid metabolism through IL-1β and IL-18 pathways, suggesting that it might play a significant role in adipose tissue as well as in metabolic diseases. This is further confirmed by a previous publication where the role of caspase-1 in adipocyte differentiation and insulin sensitivity had been demonstrated [49].

Similarly, we conducted cooperation analysis on globally regulated caspase-1 genes that we identified through meta-analysis to the same set of genes across eight Sirt-1-associated datasets. These datasets were conducted on five different mouse tissue/cells. Five of them had Sirt-1 heterozygous/homozygous KO samples compared to WT (GSE46895 [58], GSE30247 [3], GSE39778 [59], GSE28790 [60], and GSE26425 [61]), two of them had Tg-Sirt-1 samples compared to WT (GSE33101 [62] and GSE7407 [63]), and the last dataset was on Sirt-1-activated sample compared to control (GSE19102; unpublished). The percentages of significantly changed genes were different from 0.1 to 33.1%. After normalization of all the analyzed data, we found 16,196 significantly changed genes (52.0%) among 31,128 mapped genes. Totally, 9332 overlapped genes were found among these datasets when intersected (Additional file 1: Table S2).

We demonstrated above that the transcriptional effects of caspase-1 cooperated well with that of Sirt-1 in mouse adipose tissue but not in mouse liver, suggesting a possibility that Sirt-1 gene regulatory effects are tissue-specific. To examine this possibility, we performed a meta-analysis for identifying global regulatory effects of Sirt-1. We identified 28 Sirt-1 globally regulated genes, including 11 Sirt-1-promoted genes and 17 Sirt-1 globally inhibited genes (Fig. 9a). Interestingly, most of the genes that are globally regulated by Sirt-1 regulate carbohydrate metabolism. Furthermore, these genes are involved in regulating cell cycle, cell death and survival, morphology and cellular assembly. This data indicates that the tissue specificity seen in caspase-1 may be also due to the tissue-specific functions associated with its substrates as well.

**Fig. 10:**
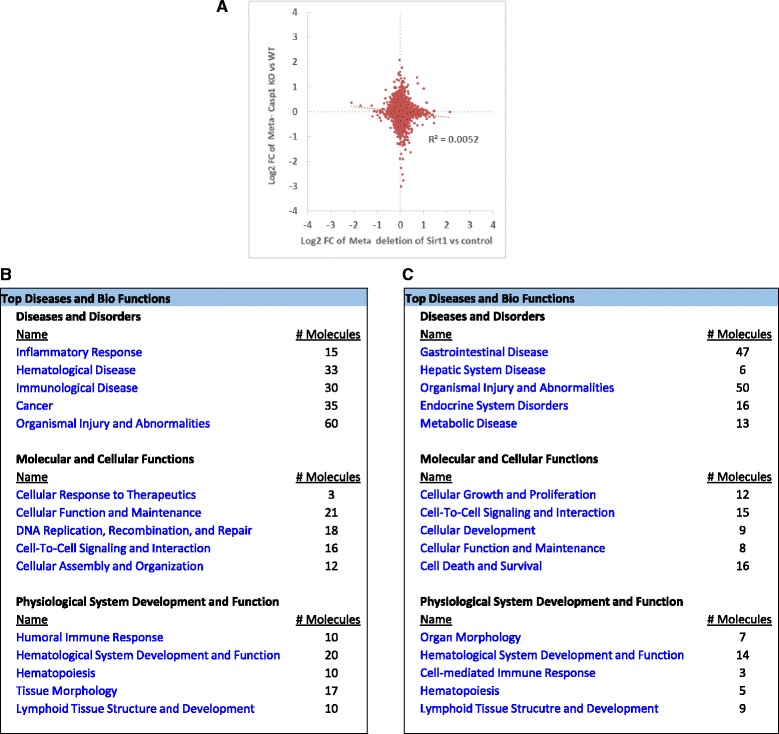
Cooperation analysis shows that Sirt-1 and caspase-1 do not cooperate in regulating genes with high fold expression changes. **a** FC/FC plot comparing Log2 FC of genes for meta-Sirt-1 (deletion of Sirt-1 vs. WT or control vs. transgenic Sirt-1 or Sirt-1 treatment) (*x-axis*) and parallel meta-Caspase-1 KO vs. WT (*y-axis*). **b** and **c**. Further analysis by Ingenuity pathway revealed the top 5 signatures regulated by genes that are cooperated with caspase-1 and Sirt-1 (**b**) and the pathways regulated by non-cooperation genes (**c**) (*p* < 0.05)

We then applied the cooperation analysis to determine the meta-analysis-generated global regulatory effects of caspase-1 with the global regulatory effects of Sirt-1. The results in Fig. 10 showed by the Log2 (fold-change, FC)/Log2 (fold-change, FC) plots using the method reported [50], that the expression of all the meta-analyzed genes regulated by Casp1 KO and Sirt-1 treatment were evenly distributed in the four quadrants (*R* ^2^ = 0.0052). In all four quadrants, there were no significant expression changes of genes (fold change >2) and these genes were found to be regulated either with or without cooperation between caspase-1 and Sirt-1.

## Discussion

Recent progress has clearly demonstrated that caspase-1/inflammasome pathway plays a critical role in regulating innate immune system by sensing PAMPs and DAMPs, induce pro-inflammatory cytokine maturation, promote inflammatory cell death (pyroptosis), induce histone modification and unconventional secretion, inhibits glycolysis, and regulate cell survival [7, 64]. In addition, myocardial-specific overexpression of caspase-1 induces a significant increase in cardiomyocyte death in young mice without any increase in tissue or plasma levels of IL-1β or other inflammatory mediators [65]. How caspase-1 regulates such broad biological functions is yet unknown.

Traditionally, signal transduction is realized by kinase-mediated protein phosphorylation and phosphatase-mediated de-phosphorylation. Recent progress has shown that proteases can also function as important regulators in signal transduction [66] with several important features including: (i) substrate specificity, (ii) nonreversible signal transduction, (iii) non-transcription- and non-translation-dependent, and (iv) non-stable protein complexes for protease-substrate interactions, etc. We [9] and others have previously shown that caspase-1 is functional as a proteinase and can cleave as many as 59 [67] to 121 [68] experimentally identified protein substrates, potentially cleaves 509 protein substrates encoded by human genome [69]. Therefore, caspase-1-regulated biological functions are broader than that carried out by three well-characterized substrates including two pro-inflammatory cytokines IL-1β, IL-18, and histone deacetylase Sirt-1. For example, it has been found that caspase-1 can also regulate nuclear translocation of novel anti-inflammatory cytokine IL-37 [70] (previously termed IL-1F7) [9], activation of sphingosine kinase 2 [71], maturation of Toll-like receptor adaptor Mal [72], and secretion of retinoic acid-inducible gene-I [73]. All these attributed functions of caspase-1 was mediated independently form IL-1β, IL-18, and Sirt-1.

In order to determine new tissue-specific and global gene regulatory functions of caspase-1, we took a novel microarray data analysis approach using Venn analysis, cooperation analysis, and meta-analysis methods. Based on the results from our analysis, we have made the following important findings: (1) Caspase-1 regulatory effects on majority of genes are tissue-specific. Partly, the tissue specificity we observed with caspase-1 may be due to tissue specificity seen with its substrate-like Sirt-1 (Fig. 10). Also, the tissue specificity of caspase-1 regulatory effects on genes that we observed may be due to differential expression of inflammasome components in various tissues as we previously reported [8]; (2) Our Venn analysis revealed that caspase-1 promotes the expression of four inflammatory genes and inhibits the expression of two genes that regulate mitochondrial energy metabolism globally; (3) Caspase-1 partially cooperates with Sirt-1 in gene regulation of organ injury and inflammation in the adipose tissue but not in the liver; (4) Caspase-1 cooperates with IL-1β in regulating less than half of the gene expression in mouse liver; (5) The meta-analysis identifies 40 caspase-1 globally regulated genes, suggesting that caspase-1 regulates many novel pathways; and (6) The meta-analysis identified new cooperatively and non-cooperatively regulated genes in caspase-1, IL-1β, IL-18, and Sirt-1 pathways.

As we previously mentioned in the Results section, meta-analysis of microarray data allows us to perform an integrative data analysis to synthesize and review the results from datasets that are independent but related [52]. In contrast to traditional analysis of a single set of microarray data, meta-analysis of microarray datasets has many benefits including increased sample size and statistical power, leading to integration-driven discovery [74]. Previously, Arasappan et al. had successfully used meta-analysis to group disparate microarray datasets to identify gene signature in mononuclear cells withdrawn from patients with systemic lupus erythematosus [24]. To the best of our knowledge, this is the first time that meta-analysis has ever been used to group caspase-1-, IL-1β-, IL-18-, and Sirt-1-related microarray datasets. Therefore, our methods used and the conclusions that are drawn in this study are innovative in determining new caspase-1-regulated signal pathways.

Our data indicates that caspase-1 is involved in a myriad of signaling pathways that regulate various cellular functions and pathologies, further emphasizing that caspase-1 exerts its effects independently of its well-known substrates. Previous publications have shown that caspase-1 deficiency can alter the gene expression in various tissues. Moreover, nuclear localization of caspase-1 and inflammasome components, and also formation of active nuclear inflammasomes in response to viral infections and TNF-α was previously demonstrated [21, 75]. These active inflammasome complexes may generate active caspase-1 in the nucleus, which may degrade nuclear transcription factors and histone modifiers that may subsequently alter the gene expression. In line with these observations, we also recently reported that substrates and interaction proteins of caspase-1 are localized in subcellular compartments other than the cytosol [76]. In this study, we analyzed 114 caspase-1 substrates and 38 caspase-1 interaction proteins which were all experimentally verified in recent publications [67, 68]. We reported that out of the 114 identified caspase-1 substrates, 28 were predominantly localized in the nucleus. Most interestingly, 14 out of these 28 nuclear caspase-1 substrates were shown to act as transcription factors. Similarly, we identified that 7 out of 38 identified caspase-1 interaction proteins are highly localized in the nucleus. Together, all these publications provide ample evidence that caspase-1 has the potential to make a significant impact on gene regulation.

Other than caspase-1, inflammasome component NLRP3 can function as a transcription factor in inflammasome-independent manner for type 2T helper cell (Th2) polarization [20]. Furthermore, Kerur et al. demonstrated that an active inflammasome comprised of ASC, IPAF and caspase-1 was formed in the nucleus in dermal endothelial cells in response to Kaposi sarcoma-associated viral infection [75]. Additionally, our most recent publication also suggested that out of 20 NLRs, 6 NLRs including NLRP3 and IPAF can localize in the nucleus suggesting the possibility of assembling variety of inflammasome complexes in response to different stresses [76]. Therefore, assembly of different inflammasome complexes that depend on the cell type and the nature of the cellular stress might contribute to the tissue-specific gene regulatory effects of caspase-1 indicated in this study. These variety of inflammasome complexes may differ in potency and rate to cleave caspase-1 and also may have different downstream targets resulting in distinct outcomes that may contribute to tissue-specific effects. Of note, the differential expression of caspase-1 substrates and interaction proteins also may play a role in tissue-specific gene regulatory effects of caspase-1.

Also, previously, it was shown that caspase-1 can exert biological effects independent of its protease activity. Furthermore, caspase-1 non-catalytic function in regulating 77 leaderless secretory proteins including IL-1β was reported [77, 78]. As we mentioned in the introduction, we categorized the tissues in to three tiers depending on the rate of inflammasome assembly in response to stress. We identified tissues in tier 1 that can assemble inflammasomes rapidly as the constituents that are required for inflammasome formation are readily available. Our most recent publication also reported that caspase-1 interaction proteins and substrates are also found in extracellular vesicles [76]. Therefore, it is possible that tissues in tier 1 may propagate inflammation to “inflammation privileged” (tissues that show a delayed inflammasome assembly) tissues via extracellular vesicles by transporting caspase-1 substrates, interaction proteins, and active inflammasomes to regulate gene expression and also to develop a rapid inflammatory response. Previous work had also experimentally verified the presence of active inflammasomes in extracellular vesicles [78–81].

These evidence suggest that caspase-1 may regulate gene expression via unidentified non-canonical pathways as well. Therefore, we emphasize the need for experimental verification of novel caspase-1 cytosolic and nuclear substrates. Also, our findings highlight the complexity of caspase-1 signaling in biological systems and requirement of elucidating exact mechanisms of how caspase-1 regulates gene expression in the cells.

**Fig. 11:**
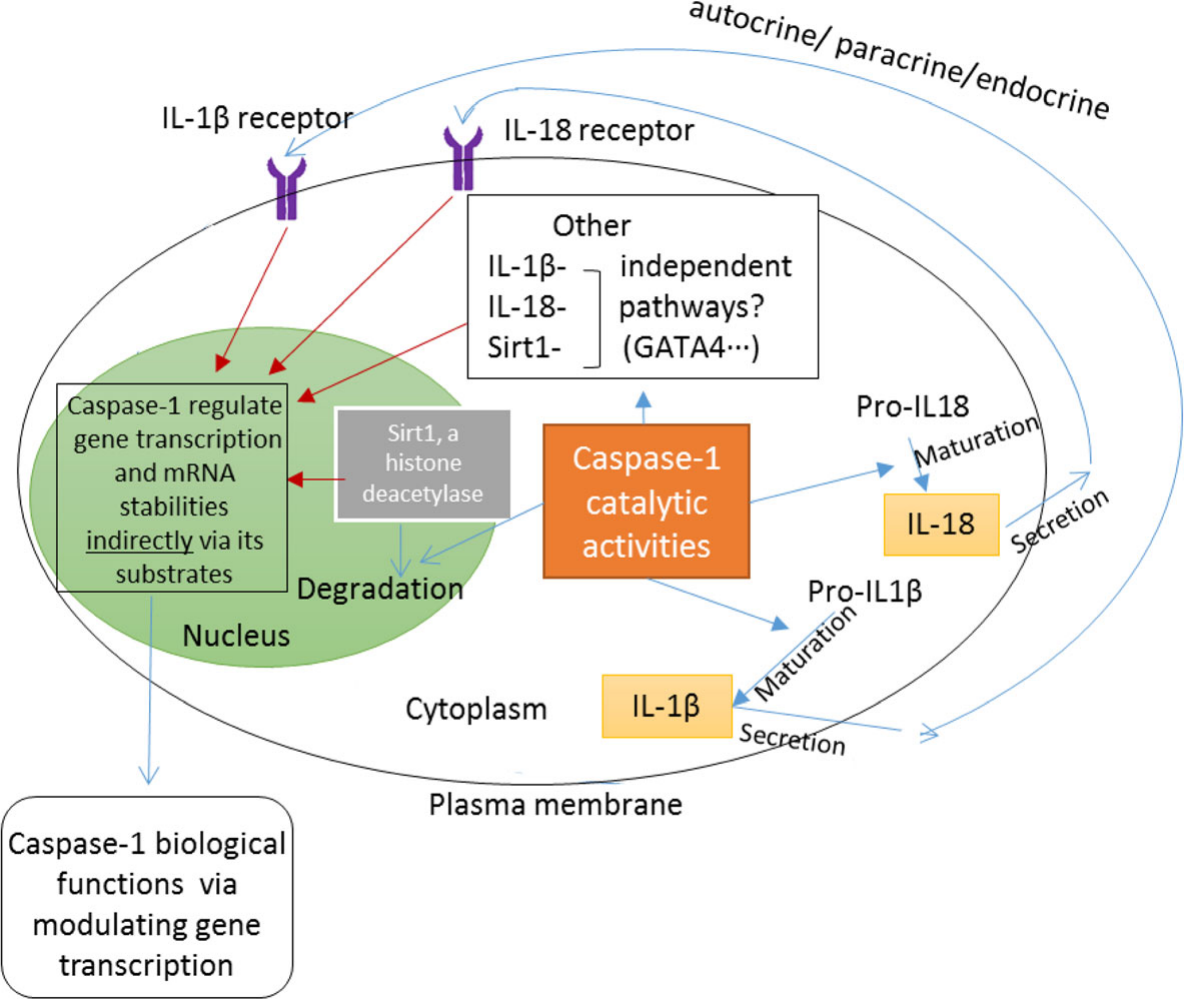
A novel working model for caspase-1-mediated gene expression. (I) In response to DAMPs and PAMPs, caspase-1 activates IL-1β and IL-18 which in turn will magnify the inflammation by enhancing secondary pro-inflammatory genes. (II) Caspase-1 may get localized to the nucleus in response to stress leading to degradation of histone modifier Sirt-1. This may lead to activation of genes that are suppressed by Sirt-1. (III) Caspase-1 can regulate gene expression by mechanisms that are independent to the mechanisms mediated by its downstream targets IL-1β, IL-18, and Sirt-1

As illustrated in Fig. 11, the results we obtained from our analysis point out a possible new functional model for caspase-1 in regulating gene expression. *First*, in response to the stimulation of DAMPs and PAMPs, caspase-1 becomes activated in the inflammasome complex. Activated caspase-1 converts pro-IL-1β and pro-IL-18 to mature pro-inflammatory IL-1β and IL-18, respectively. These two cytokines will magnify the inflammation via IL-1β and IL-18 receptor signaling to upregulate secondary pro-inflammatory genes. *Second*, caspase-1 translocate to the nucleus via its pro-domain [21], cleaves histone deacetylase Sirt-1 that leads to histone acetylation, downregulates Sirt-1-mediated activated genes, and increases the expression of genes that are inhibited by Sirt-1. *Third*, in addition, caspase-1 can fulfill its additional IL-1β, IL-18-, and Sirt-1-independent biological functions via cleaving other transcriptional factors and master protein in the nucleus that are yet to be identified. Recently, GATA4, which is a transcription factor, was identified as a caspase-1 substrate [18].

Traditionally, the enzymatic functions of proteases are tested by using their substrates as an indirect probe. Due to restrains such as time, lack of special instruments, and expertise, and also due to various tissue-specific expression patterns of substrates and physical accessibility of substrates under pathophysiological conditions, identification of all the substrates for caspase-1 is time consuming, laborious, and an expensive task. The approach utilized in this study used new angles to determine the biological functions of caspase-1 by examining the gene regulatory effects across tissues and comparing to that being regulated by three well-characterized substrates of caspase-1. Therefore, herein, we have demonstrated a new approach to determine the biological outcomes of protease-based signal transduction. Our findings may eventually lead to development of novel therapeutics for many inflammatory and non-inflammatory diseases.

This work was hypothetically driven and conducted using experimentally generated data available in public databases. It is our belief that this type of study significantly contribute to scientific progress, especially during the initial stages of identification of downstream targets and also inducing a paradigm shift in already established scientific models. We acknowledge that our analysis were done solely on microarray datasets and that it may not necessarily reflect the protein expression changes of the targets identified. Therefore, we emphasize the need for future experimental verification of the caspase-1 downstream targets identified in this study.

## Conclusions

We have used meta-analysis to successfully integrate the data derived from caspase-1-, IL-1β-, and Sirt-1-related microarray datasets. Based on our results, we conclude that caspase-1 can exert a profound effect on the transcriptome in a tissue-specific manner and caspase-1-regulated genes partially cooperate with genes regulated by its well-known downstream targets. Furthermore, our meta-analysis revealed as many as 40 genes that are potentially regulated by caspase-1. Based on the current and our most recent publications, we conclude that caspase-1 can exert a major impact on gene regulation via its nuclear substrates where many seem to play a role as transcription factors. Also, tissue specificity of caspase-1 gene regulatory effects may be due to the assembly of a variety of inflammasome complexes depending on the cellular context and the nature of the stress. Also, differential expression of caspase-1 substrates and interaction proteins also may contribute to tissue specificity of caspase-1 function.

## Additional file

Additional file 1:
**Figure S1.** Analysis flow chart of this study. **Table S1.** The details of the analyzed GEO datasets and our dataset—tissue and sample collections. **Table S2.** The characteristics of the GEO datasets and our dataset—expression-changed genes. **Table S3.** Functional annotation of caspase-1 globally regulated genes from Venn diagram analysis. **Table S4.** Profile of datasets used in the specific tissue analysis. **Table S5.** 142 significantly changed (FC > 2) genes down/upregulated by caspase-1 deficiency but are inversely up/downregulated by Sirt-1 deficiency in adipose tissue (two box-gated genes in the Fig. 2b). **Table S6.** 142 caspase-1 and Sirt-1 significantly cooperating genes and 21 non-cooperating genes with fold changes higher than two among the four quadrants in Fig. 2b. * Sirt-1 KO vs. WT, ** Caspase-1 KO vs. WT. **Table S7.** The meta-analysis identified 17 caspase-1 globally promoted genes and 23 caspase-1 globally inhibited genes. **Table S8.** The 40 caspase-1 globally regulated genes are pyroptotic genes, which are not significantly regulated by master apoptosis regulators caspase-8 and caspase-9. **Table S9.** The genes that are globally regulated by caspase-1. **Table S10.** Significantly changed genes with adj. *p* < 0.05 and FC > 1.5 comparing treatment or transgenic of IL1b to control in global from meta-analysis. IL-1β globally promotes expression of 69 genes and inhibits expression of 7 genes. **Table S11.** Sirt-1 globally promoted expression of 11 genes and inhibited the expression of 17 genes. (PPTX 116 kb)

## Abbreviations

ChIP: Chromatin immunoprecipitation
DAMP: Danger associated molecular patterns
EBI: European Bioinformatics Institute
EMBL: European Molecular Biology Laboratory
FC: Fold change
GEO: Gene Expression Omnibus
ICV: Intra-cerebroventricular
IFN-β: Interferon-beta
IL-1β: Interleukin-1 beta
NAD: Nicotinamide adenine dinucleotide
NLR: Nod-like receptors
Nnt: Nicotinamide nucleotide transhydrogenase
PAMP: Pathogen-associated molecular patterns
PRIMA: Preferred Reporting Items for Systematic Reviews and Meta-Analyses
Tg: Transgenic
TLR: Toll-like receptors
TNF: Tumor necrosis factor
TRAF: Tumor necrosis factor receptor-associated factor
