# Supplementary material for: Analyses of caspase-1-regulated transcriptomes in various tissues lead to identification of novel IL-1β-, IL-18- and sirtuin-1-independent pathways
Source: J Hematol Oncol. 2017 Feb 2;10:40. doi: 10.1186/s13045-017-0406-2 (PMC5290602; doi:10.1186/s13045-017-0406-2)
Supplement: Additional file 1: — Figure S1. Analysis flow chart of this study. Table S1. The details of the analyzed GEO datasets and our dataset—tissue and sample collections. Table S2. The characteristics of the GEO datasets and our dataset—expression-changed genes. Table S3. Functional annotation of caspase-1 globally regulated genes from Venn diagram analysis. Table S4. Profile of datasets used in the specific tissue analysis. Table S5. 142 significantly changed (FC > 2) genes down/upregulated by caspase-1 deficiency but are inversely up/downregulated by Sirt-1 deficiency in adipose tissue (two box-gated genes in the Fig. 2b). Table S6. 142 caspase-1 and Sirt-1 significantly cooperating genes and 21 non-cooperating genes with fold changes higher than two among the four quadrants in Fig. 2b. * Sirt-1 KO vs. WT, ** Caspase-1 KO vs. WT. Table S7. The meta-analysis identified 17 caspase-1 globally promoted genes and 23 caspase-1 globally inhibited genes. Table S8. The 40 caspase-1 globally regulated genes are pyroptotic genes, which are not significantly regulated by master apoptosis regulators caspase-8 and caspase-9. Table S9. The genes that are globally regulated by caspase-1. Table S10. Significantly changed genes with adj. p < 0.05 and FC > 1.5 comparing treatment or transgenic of IL1b to control in global from meta-analysis. IL-1β globally promotes expression of 69 genes and inhibits expression of 7 genes. Table S11. Sirt-1 globally promoted expression of 11 genes and inhibited the expression of 17 genes. (PPTX 116 kb) [file 13045_2017_406_MOESM1_ESM.pptx]

## Slide 1
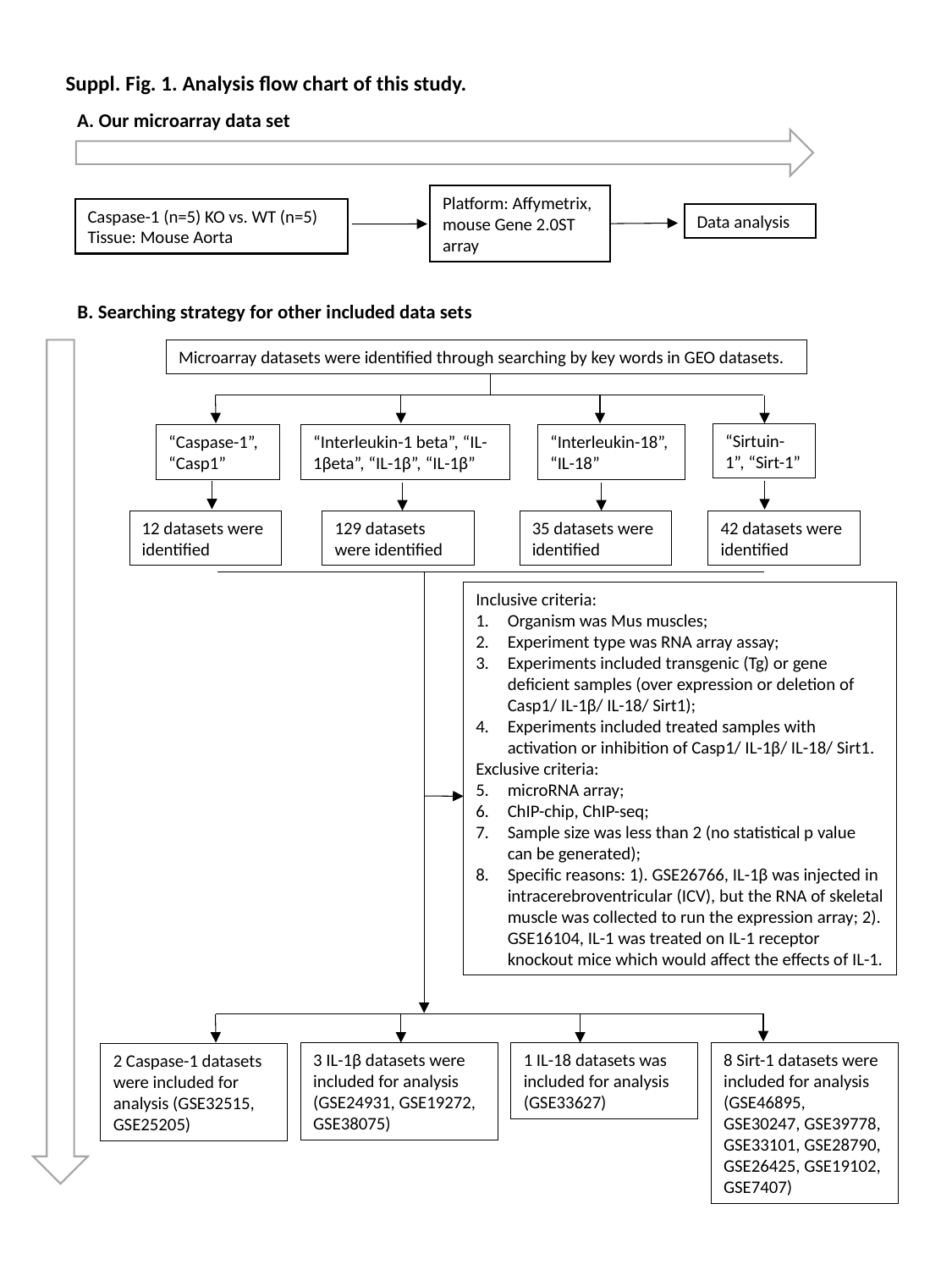

Suppl. Fig. 1. Analysis flow chart of this study.
A. Our microarray data set
Platform: Affymetrix, mouse Gene 2.0ST array
Caspase-1 (n=5) KO vs. WT (n=5)
Tissue: Mouse Aorta
Data analysis
B. Searching strategy for other included data sets
Microarray datasets were identified through searching by key words in GEO datasets.
“Sirtuin-1”, “Sirt-1”
“Caspase-1”, “Casp1”
“Interleukin-1 beta”, “IL-1βeta”, “IL-1β”, “IL-1β”
“Interleukin-18”, “IL-18”
35 datasets were identified
42 datasets were identified
129 datasets were identified
12 datasets were identified
Inclusive criteria:
Organism was Mus muscles;
Experiment type was RNA array assay;
Experiments included transgenic (Tg) or gene deficient samples (over expression or deletion of Casp1/ IL-1β/ IL-18/ Sirt1);
Experiments included treated samples with activation or inhibition of Casp1/ IL-1β/ IL-18/ Sirt1.
Exclusive criteria:
microRNA array;
ChIP-chip, ChIP-seq;
Sample size was less than 2 (no statistical p value can be generated);
Specific reasons: 1). GSE26766, IL-1β was injected in intracerebroventricular (ICV), but the RNA of skeletal muscle was collected to run the expression array; 2). GSE16104, IL-1 was treated on IL-1 receptor knockout mice which would affect the effects of IL-1.
1 IL-18 datasets was included for analysis (GSE33627)
8 Sirt-1 datasets were included for analysis (GSE46895, GSE30247, GSE39778, GSE33101, GSE28790, GSE26425, GSE19102, GSE7407)
3 IL-1β datasets were included for analysis (GSE24931, GSE19272, GSE38075)
2 Caspase-1 datasets were included for analysis (GSE32515, GSE25205)

## Slide 2
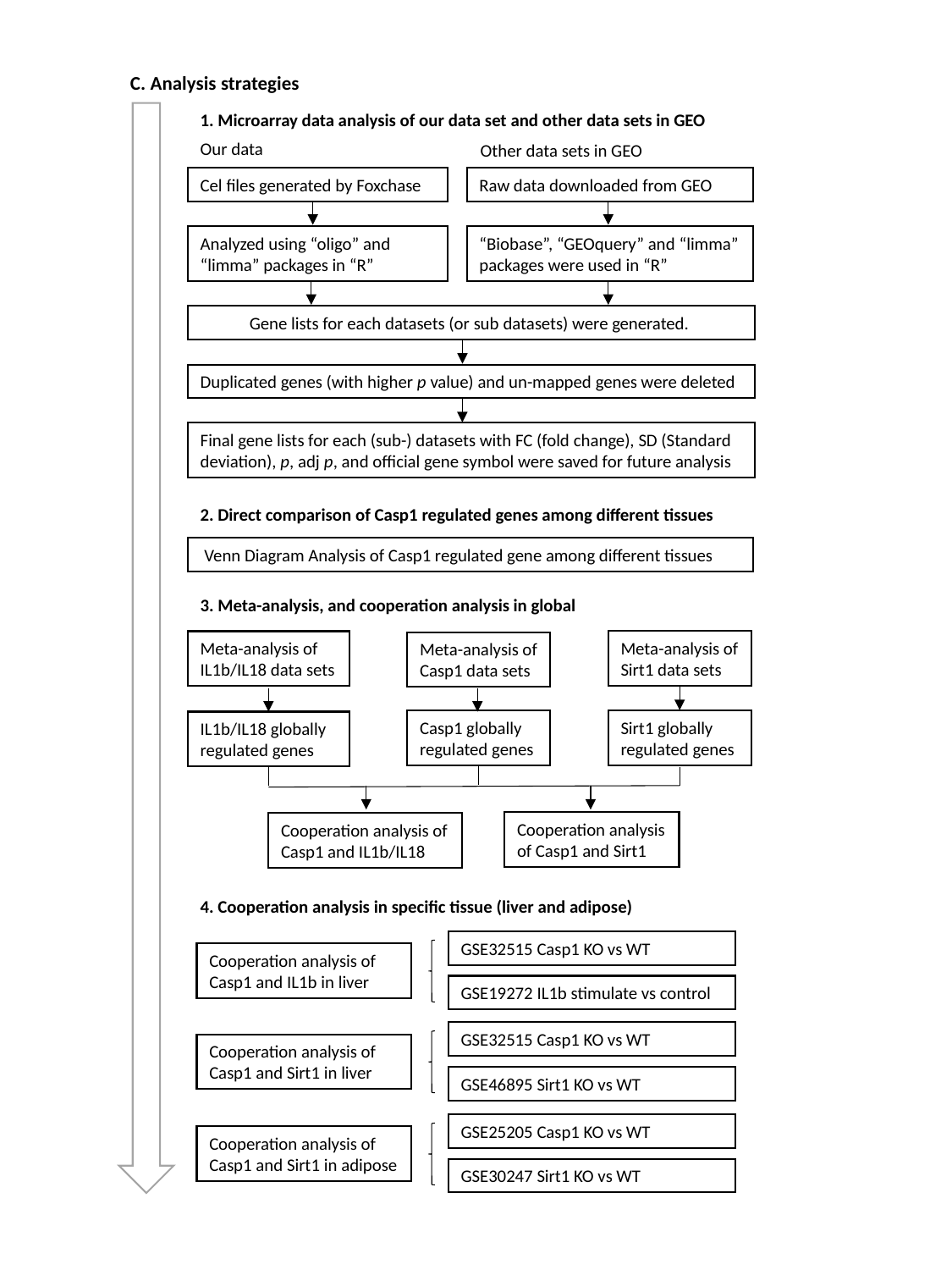

C. Analysis strategies
1. Microarray data analysis of our data set and other data sets in GEO
Our data
Other data sets in GEO
Cel files generated by Foxchase
Raw data downloaded from GEO
Analyzed using “oligo” and “limma” packages in “R”
“Biobase”, “GEOquery” and “limma” packages were used in “R”
Gene lists for each datasets (or sub datasets) were generated.
Duplicated genes (with higher p value) and un-mapped genes were deleted
Final gene lists for each (sub-) datasets with FC (fold change), SD (Standard deviation), p, adj p, and official gene symbol were saved for future analysis
2. Direct comparison of Casp1 regulated genes among different tissues
 Venn Diagram Analysis of Casp1 regulated gene among different tissues
3. Meta-analysis, and cooperation analysis in global
Meta-analysis of Sirt1 data sets
Meta-analysis of IL1b/IL18 data sets
Meta-analysis of Casp1 data sets
Casp1 globally regulated genes
Sirt1 globally regulated genes
IL1b/IL18 globally regulated genes
Cooperation analysis of Casp1 and Sirt1
Cooperation analysis of Casp1 and IL1b/IL18
4. Cooperation analysis in specific tissue (liver and adipose)
GSE32515 Casp1 KO vs WT
Cooperation analysis of Casp1 and IL1b in liver
GSE19272 IL1b stimulate vs control
GSE32515 Casp1 KO vs WT
Cooperation analysis of Casp1 and Sirt1 in liver
GSE46895 Sirt1 KO vs WT
GSE25205 Casp1 KO vs WT
Cooperation analysis of Casp1 and Sirt1 in adipose
GSE30247 Sirt1 KO vs WT

## Slide 3
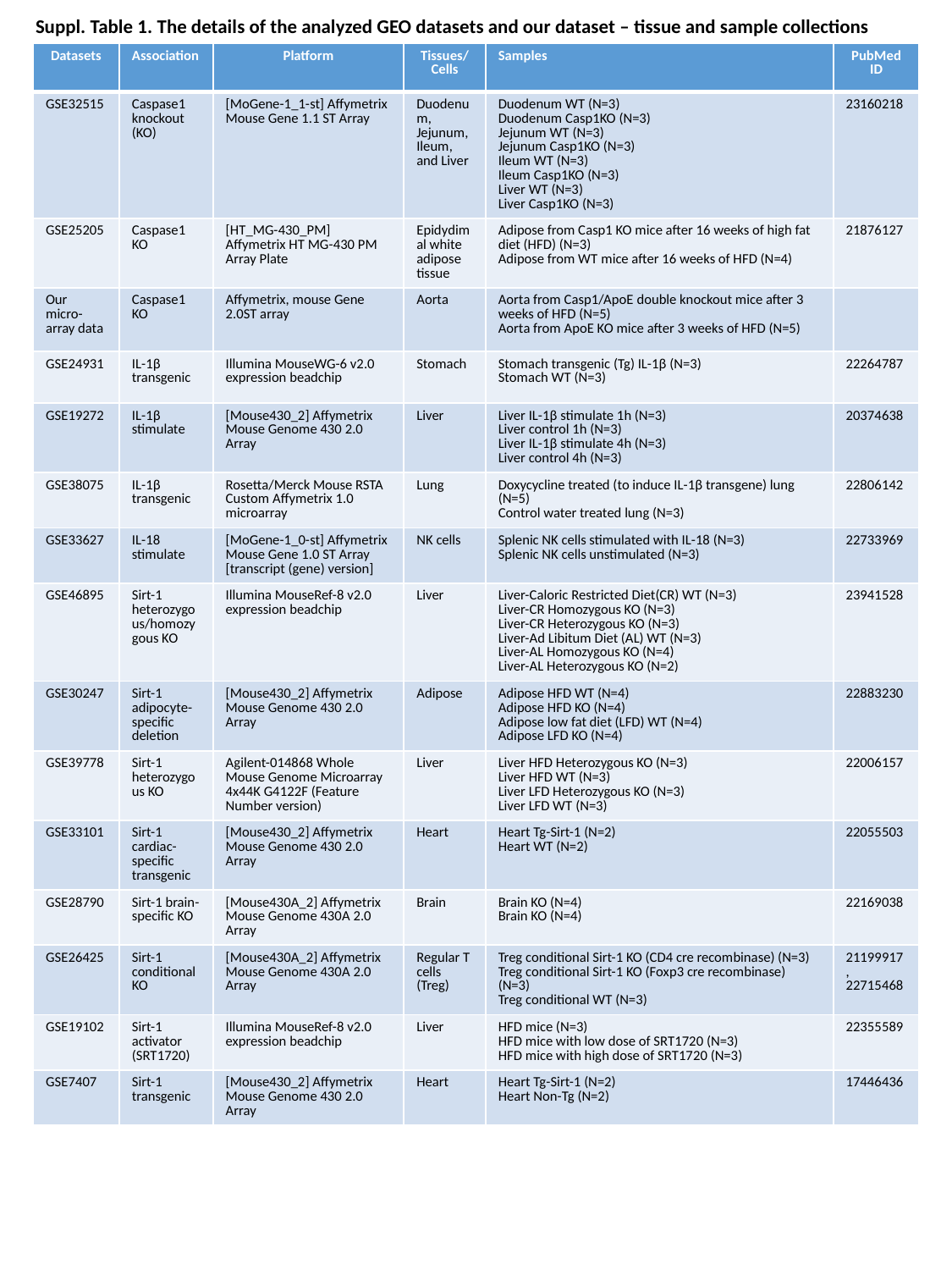

Suppl. Table 1. The details of the analyzed GEO datasets and our dataset – tissue and sample collections
| Datasets | Association | Platform | Tissues/ Cells | Samples | PubMed ID |
| --- | --- | --- | --- | --- | --- |
| GSE32515 | Caspase1 knockout (KO) | [MoGene-1\_1-st] Affymetrix Mouse Gene 1.1 ST Array | Duodenum, Jejunum, Ileum, and Liver | Duodenum WT (N=3) Duodenum Casp1KO (N=3) Jejunum WT (N=3) Jejunum Casp1KO (N=3) Ileum WT (N=3) Ileum Casp1KO (N=3) Liver WT (N=3) Liver Casp1KO (N=3) | 23160218 |
| GSE25205 | Caspase1 KO | [HT\_MG-430\_PM] Affymetrix HT MG-430 PM Array Plate | Epidydimal white adipose tissue | Adipose from Casp1 KO mice after 16 weeks of high fat diet (HFD) (N=3) Adipose from WT mice after 16 weeks of HFD (N=4) | 21876127 |
| Our micro-array data | Caspase1 KO | Affymetrix, mouse Gene 2.0ST array | Aorta | Aorta from Casp1/ApoE double knockout mice after 3 weeks of HFD (N=5) Aorta from ApoE KO mice after 3 weeks of HFD (N=5) | |
| GSE24931 | IL-1β transgenic | Illumina MouseWG-6 v2.0 expression beadchip | Stomach | Stomach transgenic (Tg) IL-1β (N=3) Stomach WT (N=3) | 22264787 |
| GSE19272 | IL-1β stimulate | [Mouse430\_2] Affymetrix Mouse Genome 430 2.0 Array | Liver | Liver IL-1β stimulate 1h (N=3) Liver control 1h (N=3) Liver IL-1β stimulate 4h (N=3) Liver control 4h (N=3) | 20374638 |
| GSE38075 | IL-1β transgenic | Rosetta/Merck Mouse RSTA Custom Affymetrix 1.0 microarray | Lung | Doxycycline treated (to induce IL-1β transgene) lung (N=5) Control water treated lung (N=3) | 22806142 |
| GSE33627 | IL-18 stimulate | [MoGene-1\_0-st] Affymetrix Mouse Gene 1.0 ST Array [transcript (gene) version] | NK cells | Splenic NK cells stimulated with IL-18 (N=3) Splenic NK cells unstimulated (N=3) | 22733969 |
| GSE46895 | Sirt-1 heterozygous/homozygous KO | Illumina MouseRef-8 v2.0 expression beadchip | Liver | Liver-Caloric Restricted Diet(CR) WT (N=3) Liver-CR Homozygous KO (N=3) Liver-CR Heterozygous KO (N=3) Liver-Ad Libitum Diet (AL) WT (N=3) Liver-AL Homozygous KO (N=4) Liver-AL Heterozygous KO (N=2) | 23941528 |
| GSE30247 | Sirt-1 adipocyte-specific deletion | [Mouse430\_2] Affymetrix Mouse Genome 430 2.0 Array | Adipose | Adipose HFD WT (N=4) Adipose HFD KO (N=4) Adipose low fat diet (LFD) WT (N=4) Adipose LFD KO (N=4) | 22883230 |
| GSE39778 | Sirt-1 heterozygous KO | Agilent-014868 Whole Mouse Genome Microarray 4x44K G4122F (Feature Number version) | Liver | Liver HFD Heterozygous KO (N=3) Liver HFD WT (N=3) Liver LFD Heterozygous KO (N=3) Liver LFD WT (N=3) | 22006157 |
| GSE33101 | Sirt-1 cardiac-specific transgenic | [Mouse430\_2] Affymetrix Mouse Genome 430 2.0 Array | Heart | Heart Tg-Sirt-1 (N=2) Heart WT (N=2) | 22055503 |
| GSE28790 | Sirt-1 brain-specific KO | [Mouse430A\_2] Affymetrix Mouse Genome 430A 2.0 Array | Brain | Brain KO (N=4) Brain KO (N=4) | 22169038 |
| GSE26425 | Sirt-1 conditional KO | [Mouse430A\_2] Affymetrix Mouse Genome 430A 2.0 Array | Regular T cells (Treg) | Treg conditional Sirt-1 KO (CD4 cre recombinase) (N=3) Treg conditional Sirt-1 KO (Foxp3 cre recombinase) (N=3) Treg conditional WT (N=3) | 21199917, 22715468 |
| GSE19102 | Sirt-1 activator (SRT1720) | Illumina MouseRef-8 v2.0 expression beadchip | Liver | HFD mice (N=3) HFD mice with low dose of SRT1720 (N=3) HFD mice with high dose of SRT1720 (N=3) | 22355589 |
| GSE7407 | Sirt-1 transgenic | [Mouse430\_2] Affymetrix Mouse Genome 430 2.0 Array | Heart | Heart Tg-Sirt-1 (N=2) Heart Non-Tg (N=2) | 17446436 |

## Slide 4
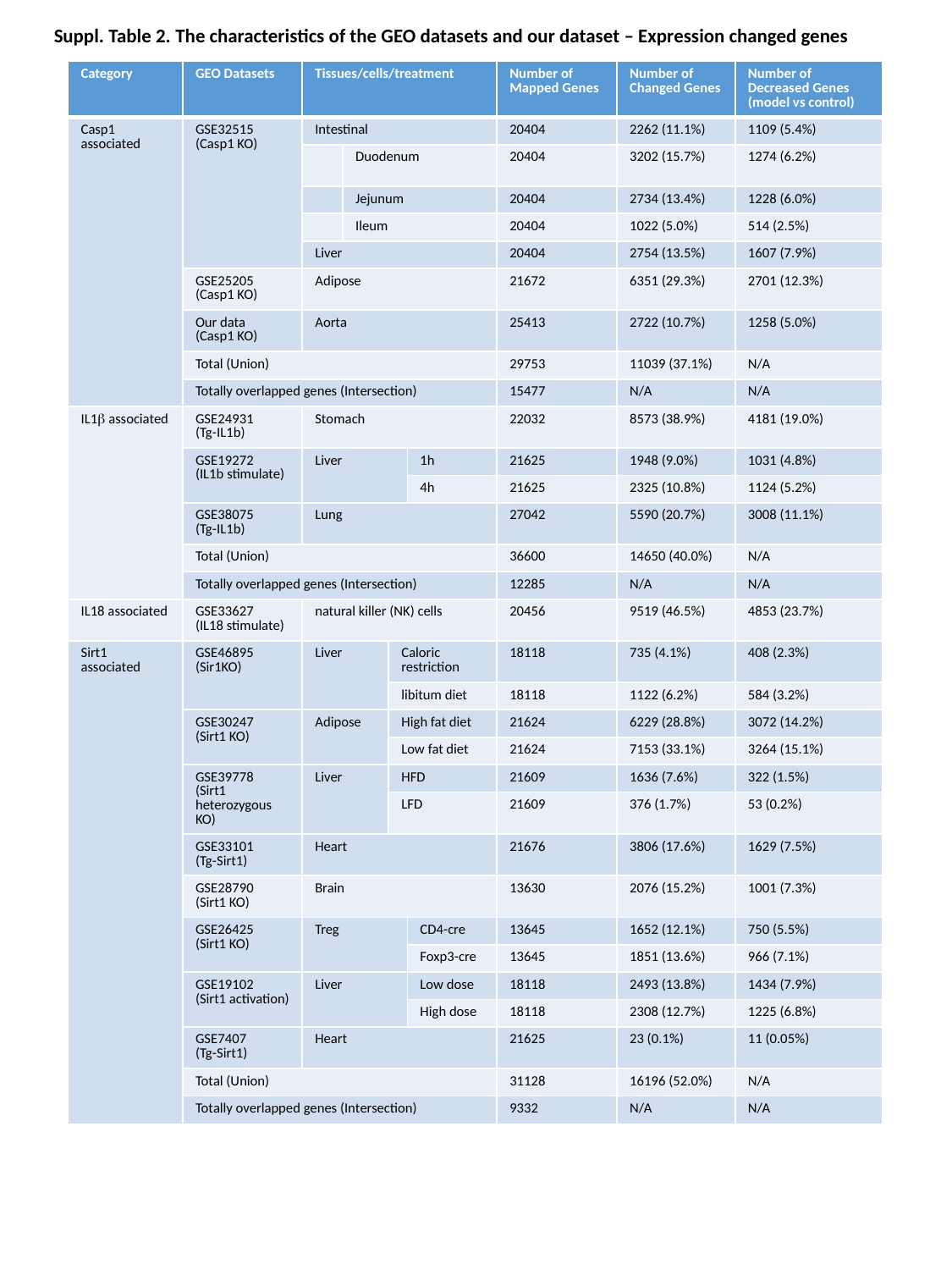

Suppl. Table 2. The characteristics of the GEO datasets and our dataset – Expression changed genes
| Category | GEO Datasets | Tissues/cells/treatment | | | | Number of Mapped Genes | Number of Changed Genes | Number of Decreased Genes (model vs control) |
| --- | --- | --- | --- | --- | --- | --- | --- | --- |
| Casp1 associated | GSE32515 (Casp1 KO) | Intestinal | | | | 20404 | 2262 (11.1%) | 1109 (5.4%) |
| | | | Duodenum | | | 20404 | 3202 (15.7%) | 1274 (6.2%) |
| | | | Jejunum | | | 20404 | 2734 (13.4%) | 1228 (6.0%) |
| | | | Ileum | | | 20404 | 1022 (5.0%) | 514 (2.5%) |
| | | Liver | | | | 20404 | 2754 (13.5%) | 1607 (7.9%) |
| | GSE25205 (Casp1 KO) | Adipose | | | | 21672 | 6351 (29.3%) | 2701 (12.3%) |
| | Our data (Casp1 KO) | Aorta | | | | 25413 | 2722 (10.7%) | 1258 (5.0%) |
| | Total (Union) | | | | | 29753 | 11039 (37.1%) | N/A |
| | Totally overlapped genes (Intersection) | | | | | 15477 | N/A | N/A |
| IL1b associated | GSE24931 (Tg-IL1b) | Stomach | | | | 22032 | 8573 (38.9%) | 4181 (19.0%) |
| | GSE19272 (IL1b stimulate) | Liver | | | 1h | 21625 | 1948 (9.0%) | 1031 (4.8%) |
| | | | | | 4h | 21625 | 2325 (10.8%) | 1124 (5.2%) |
| | GSE38075 (Tg-IL1b) | Lung | | | | 27042 | 5590 (20.7%) | 3008 (11.1%) |
| | Total (Union) | | | | | 36600 | 14650 (40.0%) | N/A |
| | Totally overlapped genes (Intersection) | | | | | 12285 | N/A | N/A |
| IL18 associated | GSE33627 (IL18 stimulate) | natural killer (NK) cells | | | | 20456 | 9519 (46.5%) | 4853 (23.7%) |
| Sirt1 associated | GSE46895 (Sir1KO) | Liver | | Caloric restriction | | 18118 | 735 (4.1%) | 408 (2.3%) |
| | | | | libitum diet | | 18118 | 1122 (6.2%) | 584 (3.2%) |
| | GSE30247 (Sirt1 KO) | Adipose | | High fat diet | | 21624 | 6229 (28.8%) | 3072 (14.2%) |
| | | | | Low fat diet | | 21624 | 7153 (33.1%) | 3264 (15.1%) |
| | GSE39778 (Sirt1 heterozygous KO) | Liver | | HFD | | 21609 | 1636 (7.6%) | 322 (1.5%) |
| | | | | LFD | | 21609 | 376 (1.7%) | 53 (0.2%) |
| | GSE33101 (Tg-Sirt1) | Heart | | | | 21676 | 3806 (17.6%) | 1629 (7.5%) |
| | GSE28790 (Sirt1 KO) | Brain | | | | 13630 | 2076 (15.2%) | 1001 (7.3%) |
| | GSE26425 (Sirt1 KO) | Treg | | | CD4-cre | 13645 | 1652 (12.1%) | 750 (5.5%) |
| | | | | | Foxp3-cre | 13645 | 1851 (13.6%) | 966 (7.1%) |
| | GSE19102 (Sirt1 activation) | Liver | | | Low dose | 18118 | 2493 (13.8%) | 1434 (7.9%) |
| | | | | | High dose | 18118 | 2308 (12.7%) | 1225 (6.8%) |
| | GSE7407 (Tg-Sirt1) | Heart | | | | 21625 | 23 (0.1%) | 11 (0.05%) |
| | Total (Union) | | | | | 31128 | 16196 (52.0%) | N/A |
| | Totally overlapped genes (Intersection) | | | | | 9332 | N/A | N/A |

## Slide 5
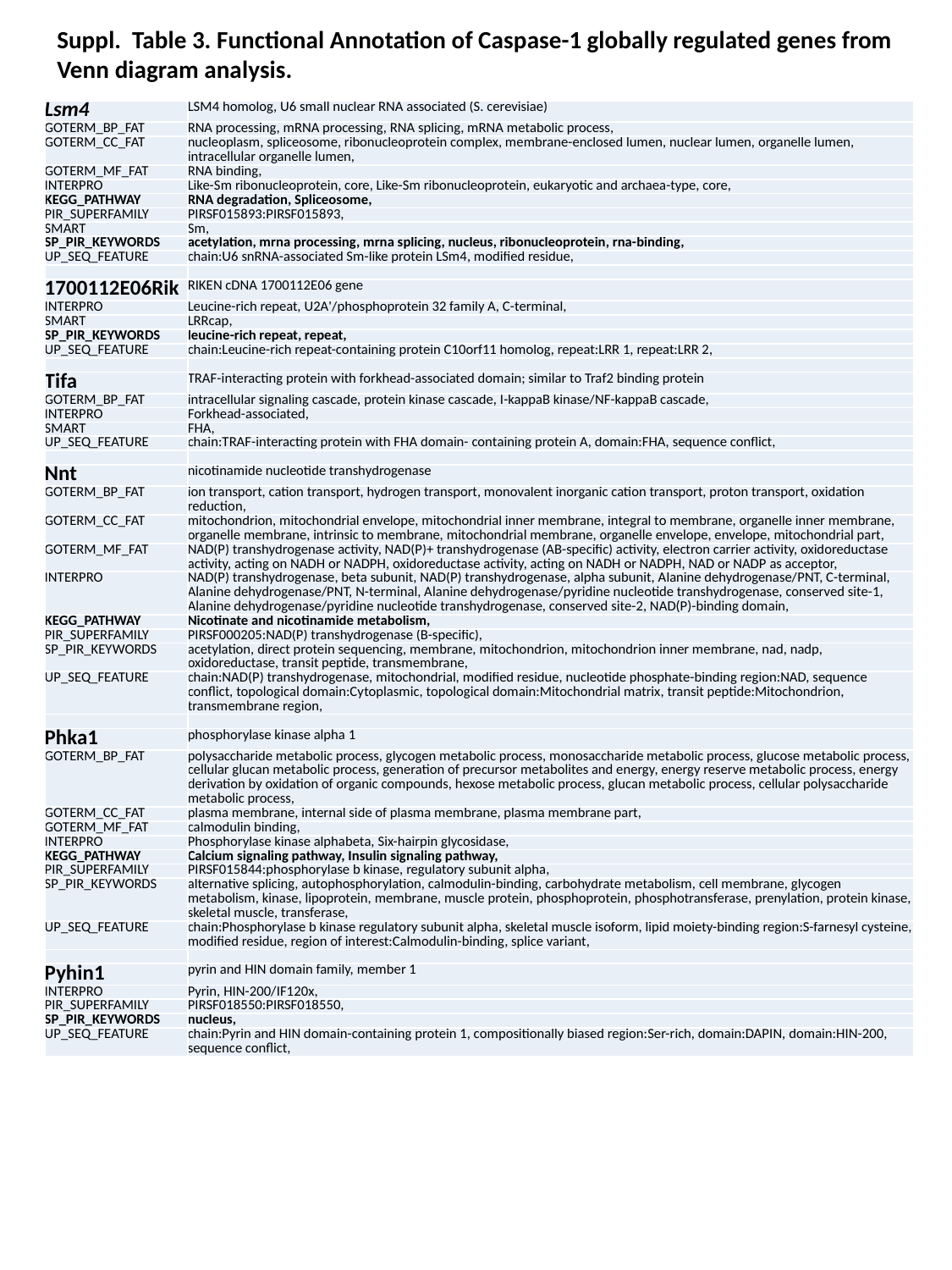

Suppl. Table 3. Functional Annotation of Caspase-1 globally regulated genes from Venn diagram analysis.
| Lsm4 | LSM4 homolog, U6 small nuclear RNA associated (S. cerevisiae) |
| --- | --- |
| GOTERM\_BP\_FAT | RNA processing, mRNA processing, RNA splicing, mRNA metabolic process, |
| GOTERM\_CC\_FAT | nucleoplasm, spliceosome, ribonucleoprotein complex, membrane-enclosed lumen, nuclear lumen, organelle lumen, intracellular organelle lumen, |
| GOTERM\_MF\_FAT | RNA binding, |
| INTERPRO | Like-Sm ribonucleoprotein, core, Like-Sm ribonucleoprotein, eukaryotic and archaea-type, core, |
| KEGG\_PATHWAY | RNA degradation, Spliceosome, |
| PIR\_SUPERFAMILY | PIRSF015893:PIRSF015893, |
| SMART | Sm, |
| SP\_PIR\_KEYWORDS | acetylation, mrna processing, mrna splicing, nucleus, ribonucleoprotein, rna-binding, |
| UP\_SEQ\_FEATURE | chain:U6 snRNA-associated Sm-like protein LSm4, modified residue, |
| | |
| 1700112E06Rik | RIKEN cDNA 1700112E06 gene |
| INTERPRO | Leucine-rich repeat, U2A'/phosphoprotein 32 family A, C-terminal, |
| SMART | LRRcap, |
| SP\_PIR\_KEYWORDS | leucine-rich repeat, repeat, |
| UP\_SEQ\_FEATURE | chain:Leucine-rich repeat-containing protein C10orf11 homolog, repeat:LRR 1, repeat:LRR 2, |
| | |
| Tifa | TRAF-interacting protein with forkhead-associated domain; similar to Traf2 binding protein |
| GOTERM\_BP\_FAT | intracellular signaling cascade, protein kinase cascade, I-kappaB kinase/NF-kappaB cascade, |
| INTERPRO | Forkhead-associated, |
| SMART | FHA, |
| UP\_SEQ\_FEATURE | chain:TRAF-interacting protein with FHA domain- containing protein A, domain:FHA, sequence conflict, |
| | |
| Nnt | nicotinamide nucleotide transhydrogenase |
| GOTERM\_BP\_FAT | ion transport, cation transport, hydrogen transport, monovalent inorganic cation transport, proton transport, oxidation reduction, |
| GOTERM\_CC\_FAT | mitochondrion, mitochondrial envelope, mitochondrial inner membrane, integral to membrane, organelle inner membrane, organelle membrane, intrinsic to membrane, mitochondrial membrane, organelle envelope, envelope, mitochondrial part, |
| GOTERM\_MF\_FAT | NAD(P) transhydrogenase activity, NAD(P)+ transhydrogenase (AB-specific) activity, electron carrier activity, oxidoreductase activity, acting on NADH or NADPH, oxidoreductase activity, acting on NADH or NADPH, NAD or NADP as acceptor, |
| INTERPRO | NAD(P) transhydrogenase, beta subunit, NAD(P) transhydrogenase, alpha subunit, Alanine dehydrogenase/PNT, C-terminal, Alanine dehydrogenase/PNT, N-terminal, Alanine dehydrogenase/pyridine nucleotide transhydrogenase, conserved site-1, Alanine dehydrogenase/pyridine nucleotide transhydrogenase, conserved site-2, NAD(P)-binding domain, |
| KEGG\_PATHWAY | Nicotinate and nicotinamide metabolism, |
| PIR\_SUPERFAMILY | PIRSF000205:NAD(P) transhydrogenase (B-specific), |
| SP\_PIR\_KEYWORDS | acetylation, direct protein sequencing, membrane, mitochondrion, mitochondrion inner membrane, nad, nadp, oxidoreductase, transit peptide, transmembrane, |
| UP\_SEQ\_FEATURE | chain:NAD(P) transhydrogenase, mitochondrial, modified residue, nucleotide phosphate-binding region:NAD, sequence conflict, topological domain:Cytoplasmic, topological domain:Mitochondrial matrix, transit peptide:Mitochondrion, transmembrane region, |
| | |
| Phka1 | phosphorylase kinase alpha 1 |
| GOTERM\_BP\_FAT | polysaccharide metabolic process, glycogen metabolic process, monosaccharide metabolic process, glucose metabolic process, cellular glucan metabolic process, generation of precursor metabolites and energy, energy reserve metabolic process, energy derivation by oxidation of organic compounds, hexose metabolic process, glucan metabolic process, cellular polysaccharide metabolic process, |
| GOTERM\_CC\_FAT | plasma membrane, internal side of plasma membrane, plasma membrane part, |
| GOTERM\_MF\_FAT | calmodulin binding, |
| INTERPRO | Phosphorylase kinase alphabeta, Six-hairpin glycosidase, |
| KEGG\_PATHWAY | Calcium signaling pathway, Insulin signaling pathway, |
| PIR\_SUPERFAMILY | PIRSF015844:phosphorylase b kinase, regulatory subunit alpha, |
| SP\_PIR\_KEYWORDS | alternative splicing, autophosphorylation, calmodulin-binding, carbohydrate metabolism, cell membrane, glycogen metabolism, kinase, lipoprotein, membrane, muscle protein, phosphoprotein, phosphotransferase, prenylation, protein kinase, skeletal muscle, transferase, |
| UP\_SEQ\_FEATURE | chain:Phosphorylase b kinase regulatory subunit alpha, skeletal muscle isoform, lipid moiety-binding region:S-farnesyl cysteine, modified residue, region of interest:Calmodulin-binding, splice variant, |
| | |
| Pyhin1 | pyrin and HIN domain family, member 1 |
| INTERPRO | Pyrin, HIN-200/IF120x, |
| PIR\_SUPERFAMILY | PIRSF018550:PIRSF018550, |
| SP\_PIR\_KEYWORDS | nucleus, |
| UP\_SEQ\_FEATURE | chain:Pyrin and HIN domain-containing protein 1, compositionally biased region:Ser-rich, domain:DAPIN, domain:HIN-200, sequence conflict, |

## Slide 6
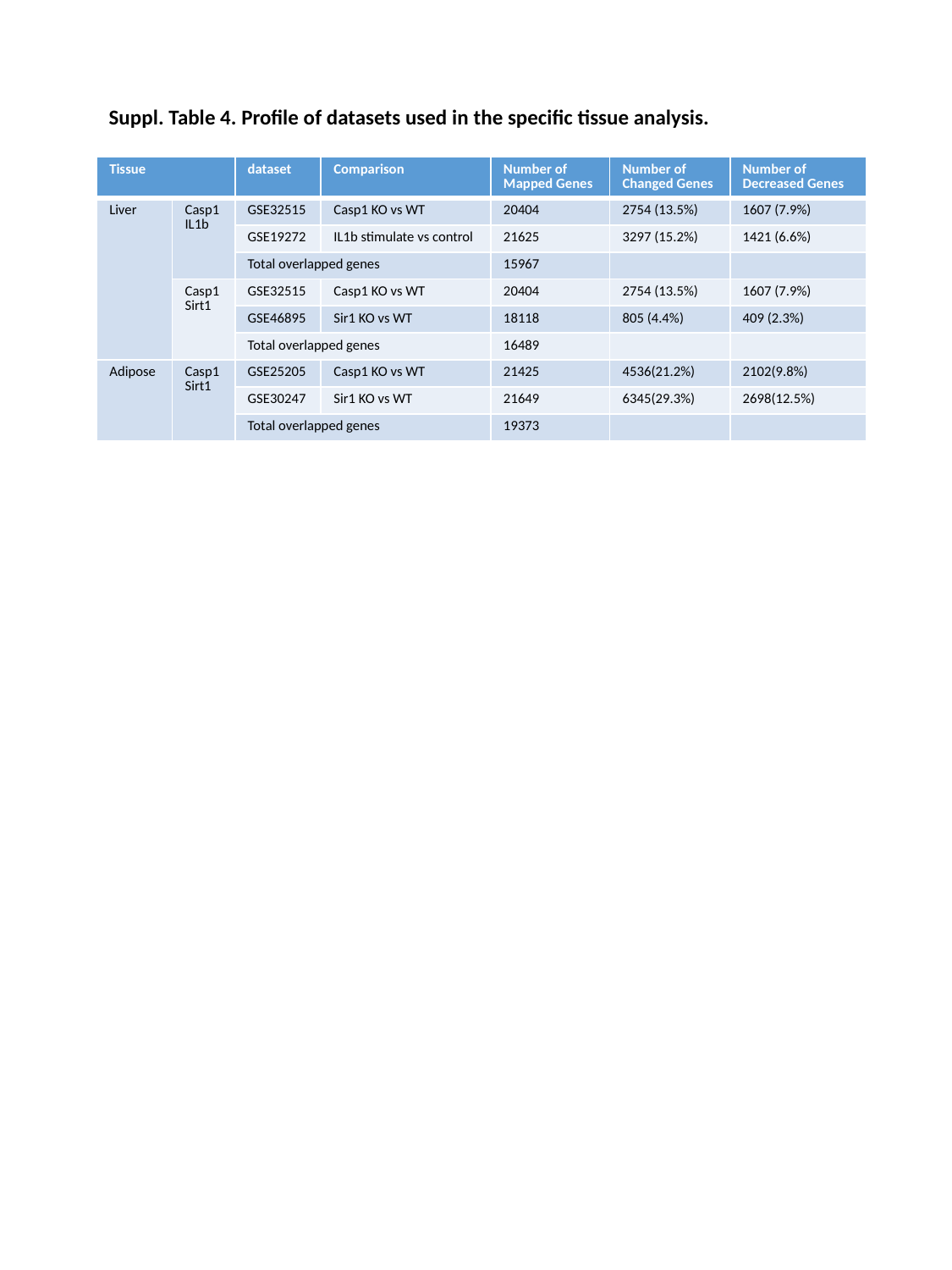

Suppl. Table 4. Profile of datasets used in the specific tissue analysis.
| Tissue | | dataset | Comparison | Number of Mapped Genes | Number of Changed Genes | Number of Decreased Genes |
| --- | --- | --- | --- | --- | --- | --- |
| Liver | Casp1 IL1b | GSE32515 | Casp1 KO vs WT | 20404 | 2754 (13.5%) | 1607 (7.9%) |
| | | GSE19272 | IL1b stimulate vs control | 21625 | 3297 (15.2%) | 1421 (6.6%) |
| | | Total overlapped genes | | 15967 | | |
| | Casp1 Sirt1 | GSE32515 | Casp1 KO vs WT | 20404 | 2754 (13.5%) | 1607 (7.9%) |
| | | GSE46895 | Sir1 KO vs WT | 18118 | 805 (4.4%) | 409 (2.3%) |
| | | Total overlapped genes | | 16489 | | |
| Adipose | Casp1 Sirt1 | GSE25205 | Casp1 KO vs WT | 21425 | 4536(21.2%) | 2102(9.8%) |
| | | GSE30247 | Sir1 KO vs WT | 21649 | 6345(29.3%) | 2698(12.5%) |
| | | Total overlapped genes | | 19373 | | |

## Slide 7
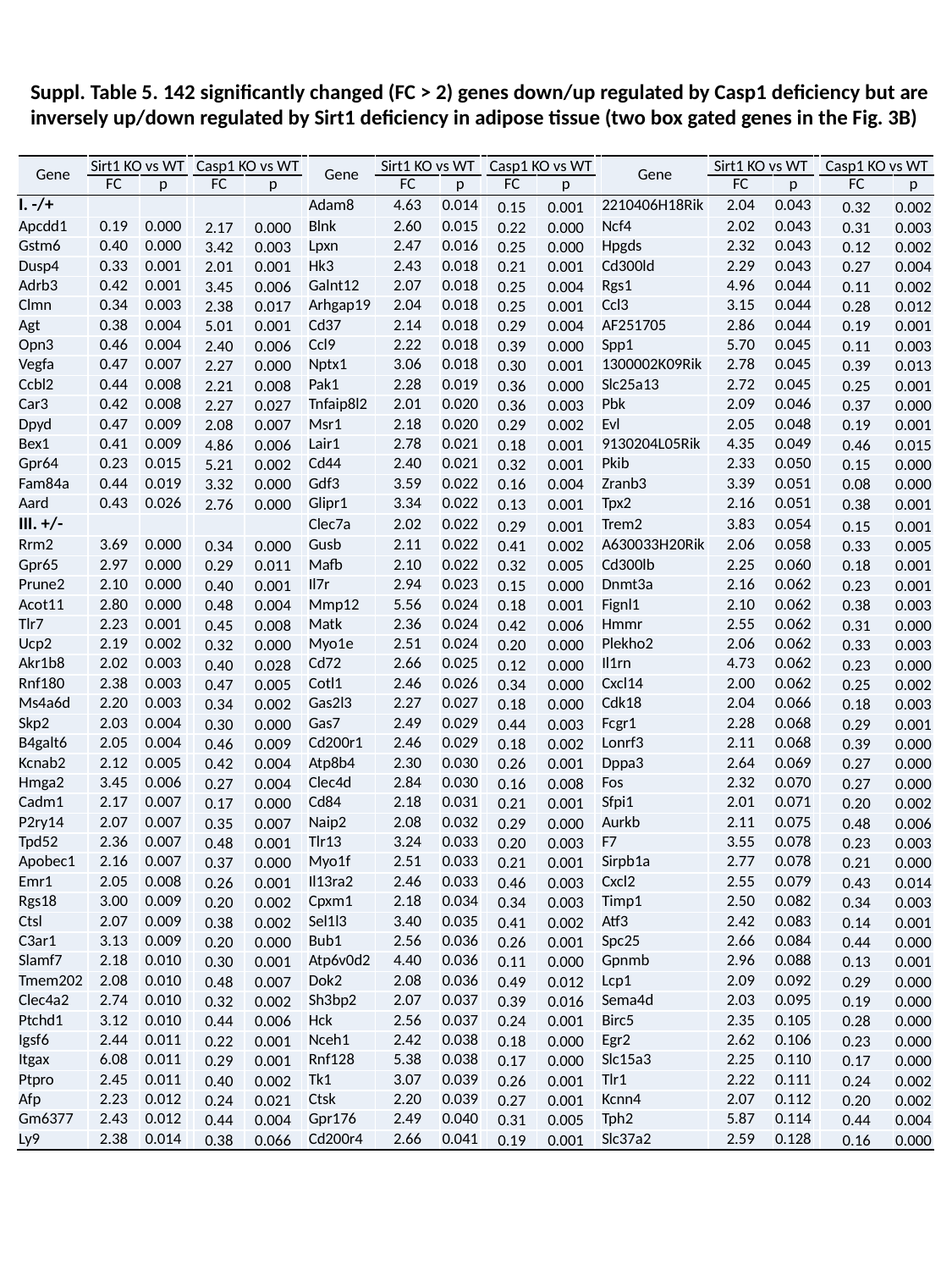

Suppl. Table 5. 142 significantly changed (FC > 2) genes down/up regulated by Casp1 deficiency but are inversely up/down regulated by Sirt1 deficiency in adipose tissue (two box gated genes in the Fig. 3B)
| Gene | Sirt1 KO vs WT | | | Casp1 KO vs WT | | | Gene | Sirt1 KO vs WT | | | Casp1 KO vs WT | | | Gene | Sirt1 KO vs WT | | | Casp1 KO vs WT | |
| --- | --- | --- | --- | --- | --- | --- | --- | --- | --- | --- | --- | --- | --- | --- | --- | --- | --- | --- | --- |
| | FC | p | | FC | p | | | FC | p | | FC | p | | | FC | p | | FC | p |
| I. -/+ | | | | | | | Adam8 | 4.63 | 0.014 | | 0.15 | 0.001 | | 2210406H18Rik | 2.04 | 0.043 | | 0.32 | 0.002 |
| Apcdd1 | 0.19 | 0.000 | | 2.17 | 0.000 | | Blnk | 2.60 | 0.015 | | 0.22 | 0.000 | | Ncf4 | 2.02 | 0.043 | | 0.31 | 0.003 |
| Gstm6 | 0.40 | 0.000 | | 3.42 | 0.003 | | Lpxn | 2.47 | 0.016 | | 0.25 | 0.000 | | Hpgds | 2.32 | 0.043 | | 0.12 | 0.002 |
| Dusp4 | 0.33 | 0.001 | | 2.01 | 0.001 | | Hk3 | 2.43 | 0.018 | | 0.21 | 0.001 | | Cd300ld | 2.29 | 0.043 | | 0.27 | 0.004 |
| Adrb3 | 0.42 | 0.001 | | 3.45 | 0.006 | | Galnt12 | 2.07 | 0.018 | | 0.25 | 0.004 | | Rgs1 | 4.96 | 0.044 | | 0.11 | 0.002 |
| Clmn | 0.34 | 0.003 | | 2.38 | 0.017 | | Arhgap19 | 2.04 | 0.018 | | 0.25 | 0.001 | | Ccl3 | 3.15 | 0.044 | | 0.28 | 0.012 |
| Agt | 0.38 | 0.004 | | 5.01 | 0.001 | | Cd37 | 2.14 | 0.018 | | 0.29 | 0.004 | | AF251705 | 2.86 | 0.044 | | 0.19 | 0.001 |
| Opn3 | 0.46 | 0.004 | | 2.40 | 0.006 | | Ccl9 | 2.22 | 0.018 | | 0.39 | 0.000 | | Spp1 | 5.70 | 0.045 | | 0.11 | 0.003 |
| Vegfa | 0.47 | 0.007 | | 2.27 | 0.000 | | Nptx1 | 3.06 | 0.018 | | 0.30 | 0.001 | | 1300002K09Rik | 2.78 | 0.045 | | 0.39 | 0.013 |
| Ccbl2 | 0.44 | 0.008 | | 2.21 | 0.008 | | Pak1 | 2.28 | 0.019 | | 0.36 | 0.000 | | Slc25a13 | 2.72 | 0.045 | | 0.25 | 0.001 |
| Car3 | 0.42 | 0.008 | | 2.27 | 0.027 | | Tnfaip8l2 | 2.01 | 0.020 | | 0.36 | 0.003 | | Pbk | 2.09 | 0.046 | | 0.37 | 0.000 |
| Dpyd | 0.47 | 0.009 | | 2.08 | 0.007 | | Msr1 | 2.18 | 0.020 | | 0.29 | 0.002 | | Evl | 2.05 | 0.048 | | 0.19 | 0.001 |
| Bex1 | 0.41 | 0.009 | | 4.86 | 0.006 | | Lair1 | 2.78 | 0.021 | | 0.18 | 0.001 | | 9130204L05Rik | 4.35 | 0.049 | | 0.46 | 0.015 |
| Gpr64 | 0.23 | 0.015 | | 5.21 | 0.002 | | Cd44 | 2.40 | 0.021 | | 0.32 | 0.001 | | Pkib | 2.33 | 0.050 | | 0.15 | 0.000 |
| Fam84a | 0.44 | 0.019 | | 3.32 | 0.000 | | Gdf3 | 3.59 | 0.022 | | 0.16 | 0.004 | | Zranb3 | 3.39 | 0.051 | | 0.08 | 0.000 |
| Aard | 0.43 | 0.026 | | 2.76 | 0.000 | | Glipr1 | 3.34 | 0.022 | | 0.13 | 0.001 | | Tpx2 | 2.16 | 0.051 | | 0.38 | 0.001 |
| III. +/- | | | | | | | Clec7a | 2.02 | 0.022 | | 0.29 | 0.001 | | Trem2 | 3.83 | 0.054 | | 0.15 | 0.001 |
| Rrm2 | 3.69 | 0.000 | | 0.34 | 0.000 | | Gusb | 2.11 | 0.022 | | 0.41 | 0.002 | | A630033H20Rik | 2.06 | 0.058 | | 0.33 | 0.005 |
| Gpr65 | 2.97 | 0.000 | | 0.29 | 0.011 | | Mafb | 2.10 | 0.022 | | 0.32 | 0.005 | | Cd300lb | 2.25 | 0.060 | | 0.18 | 0.001 |
| Prune2 | 2.10 | 0.000 | | 0.40 | 0.001 | | Il7r | 2.94 | 0.023 | | 0.15 | 0.000 | | Dnmt3a | 2.16 | 0.062 | | 0.23 | 0.001 |
| Acot11 | 2.80 | 0.000 | | 0.48 | 0.004 | | Mmp12 | 5.56 | 0.024 | | 0.18 | 0.001 | | Fignl1 | 2.10 | 0.062 | | 0.38 | 0.003 |
| Tlr7 | 2.23 | 0.001 | | 0.45 | 0.008 | | Matk | 2.36 | 0.024 | | 0.42 | 0.006 | | Hmmr | 2.55 | 0.062 | | 0.31 | 0.000 |
| Ucp2 | 2.19 | 0.002 | | 0.32 | 0.000 | | Myo1e | 2.51 | 0.024 | | 0.20 | 0.000 | | Plekho2 | 2.06 | 0.062 | | 0.33 | 0.003 |
| Akr1b8 | 2.02 | 0.003 | | 0.40 | 0.028 | | Cd72 | 2.66 | 0.025 | | 0.12 | 0.000 | | Il1rn | 4.73 | 0.062 | | 0.23 | 0.000 |
| Rnf180 | 2.38 | 0.003 | | 0.47 | 0.005 | | Cotl1 | 2.46 | 0.026 | | 0.34 | 0.000 | | Cxcl14 | 2.00 | 0.062 | | 0.25 | 0.002 |
| Ms4a6d | 2.20 | 0.003 | | 0.34 | 0.002 | | Gas2l3 | 2.27 | 0.027 | | 0.18 | 0.000 | | Cdk18 | 2.04 | 0.066 | | 0.18 | 0.003 |
| Skp2 | 2.03 | 0.004 | | 0.30 | 0.000 | | Gas7 | 2.49 | 0.029 | | 0.44 | 0.003 | | Fcgr1 | 2.28 | 0.068 | | 0.29 | 0.001 |
| B4galt6 | 2.05 | 0.004 | | 0.46 | 0.009 | | Cd200r1 | 2.46 | 0.029 | | 0.18 | 0.002 | | Lonrf3 | 2.11 | 0.068 | | 0.39 | 0.000 |
| Kcnab2 | 2.12 | 0.005 | | 0.42 | 0.004 | | Atp8b4 | 2.30 | 0.030 | | 0.26 | 0.001 | | Dppa3 | 2.64 | 0.069 | | 0.27 | 0.000 |
| Hmga2 | 3.45 | 0.006 | | 0.27 | 0.004 | | Clec4d | 2.84 | 0.030 | | 0.16 | 0.008 | | Fos | 2.32 | 0.070 | | 0.27 | 0.000 |
| Cadm1 | 2.17 | 0.007 | | 0.17 | 0.000 | | Cd84 | 2.18 | 0.031 | | 0.21 | 0.001 | | Sfpi1 | 2.01 | 0.071 | | 0.20 | 0.002 |
| P2ry14 | 2.07 | 0.007 | | 0.35 | 0.007 | | Naip2 | 2.08 | 0.032 | | 0.29 | 0.000 | | Aurkb | 2.11 | 0.075 | | 0.48 | 0.006 |
| Tpd52 | 2.36 | 0.007 | | 0.48 | 0.001 | | Tlr13 | 3.24 | 0.033 | | 0.20 | 0.003 | | F7 | 3.55 | 0.078 | | 0.23 | 0.003 |
| Apobec1 | 2.16 | 0.007 | | 0.37 | 0.000 | | Myo1f | 2.51 | 0.033 | | 0.21 | 0.001 | | Sirpb1a | 2.77 | 0.078 | | 0.21 | 0.000 |
| Emr1 | 2.05 | 0.008 | | 0.26 | 0.001 | | Il13ra2 | 2.46 | 0.033 | | 0.46 | 0.003 | | Cxcl2 | 2.55 | 0.079 | | 0.43 | 0.014 |
| Rgs18 | 3.00 | 0.009 | | 0.20 | 0.002 | | Cpxm1 | 2.18 | 0.034 | | 0.34 | 0.003 | | Timp1 | 2.50 | 0.082 | | 0.34 | 0.003 |
| Ctsl | 2.07 | 0.009 | | 0.38 | 0.002 | | Sel1l3 | 3.40 | 0.035 | | 0.41 | 0.002 | | Atf3 | 2.42 | 0.083 | | 0.14 | 0.001 |
| C3ar1 | 3.13 | 0.009 | | 0.20 | 0.000 | | Bub1 | 2.56 | 0.036 | | 0.26 | 0.001 | | Spc25 | 2.66 | 0.084 | | 0.44 | 0.000 |
| Slamf7 | 2.18 | 0.010 | | 0.30 | 0.001 | | Atp6v0d2 | 4.40 | 0.036 | | 0.11 | 0.000 | | Gpnmb | 2.96 | 0.088 | | 0.13 | 0.001 |
| Tmem202 | 2.08 | 0.010 | | 0.48 | 0.007 | | Dok2 | 2.08 | 0.036 | | 0.49 | 0.012 | | Lcp1 | 2.09 | 0.092 | | 0.29 | 0.000 |
| Clec4a2 | 2.74 | 0.010 | | 0.32 | 0.002 | | Sh3bp2 | 2.07 | 0.037 | | 0.39 | 0.016 | | Sema4d | 2.03 | 0.095 | | 0.19 | 0.000 |
| Ptchd1 | 3.12 | 0.010 | | 0.44 | 0.006 | | Hck | 2.56 | 0.037 | | 0.24 | 0.001 | | Birc5 | 2.35 | 0.105 | | 0.28 | 0.000 |
| Igsf6 | 2.44 | 0.011 | | 0.22 | 0.001 | | Nceh1 | 2.42 | 0.038 | | 0.18 | 0.000 | | Egr2 | 2.62 | 0.106 | | 0.23 | 0.000 |
| Itgax | 6.08 | 0.011 | | 0.29 | 0.001 | | Rnf128 | 5.38 | 0.038 | | 0.17 | 0.000 | | Slc15a3 | 2.25 | 0.110 | | 0.17 | 0.000 |
| Ptpro | 2.45 | 0.011 | | 0.40 | 0.002 | | Tk1 | 3.07 | 0.039 | | 0.26 | 0.001 | | Tlr1 | 2.22 | 0.111 | | 0.24 | 0.002 |
| Afp | 2.23 | 0.012 | | 0.24 | 0.021 | | Ctsk | 2.20 | 0.039 | | 0.27 | 0.001 | | Kcnn4 | 2.07 | 0.112 | | 0.20 | 0.002 |
| Gm6377 | 2.43 | 0.012 | | 0.44 | 0.004 | | Gpr176 | 2.49 | 0.040 | | 0.31 | 0.005 | | Tph2 | 5.87 | 0.114 | | 0.44 | 0.004 |
| Ly9 | 2.38 | 0.014 | | 0.38 | 0.066 | | Cd200r4 | 2.66 | 0.041 | | 0.19 | 0.001 | | Slc37a2 | 2.59 | 0.128 | | 0.16 | 0.000 |

## Slide 8
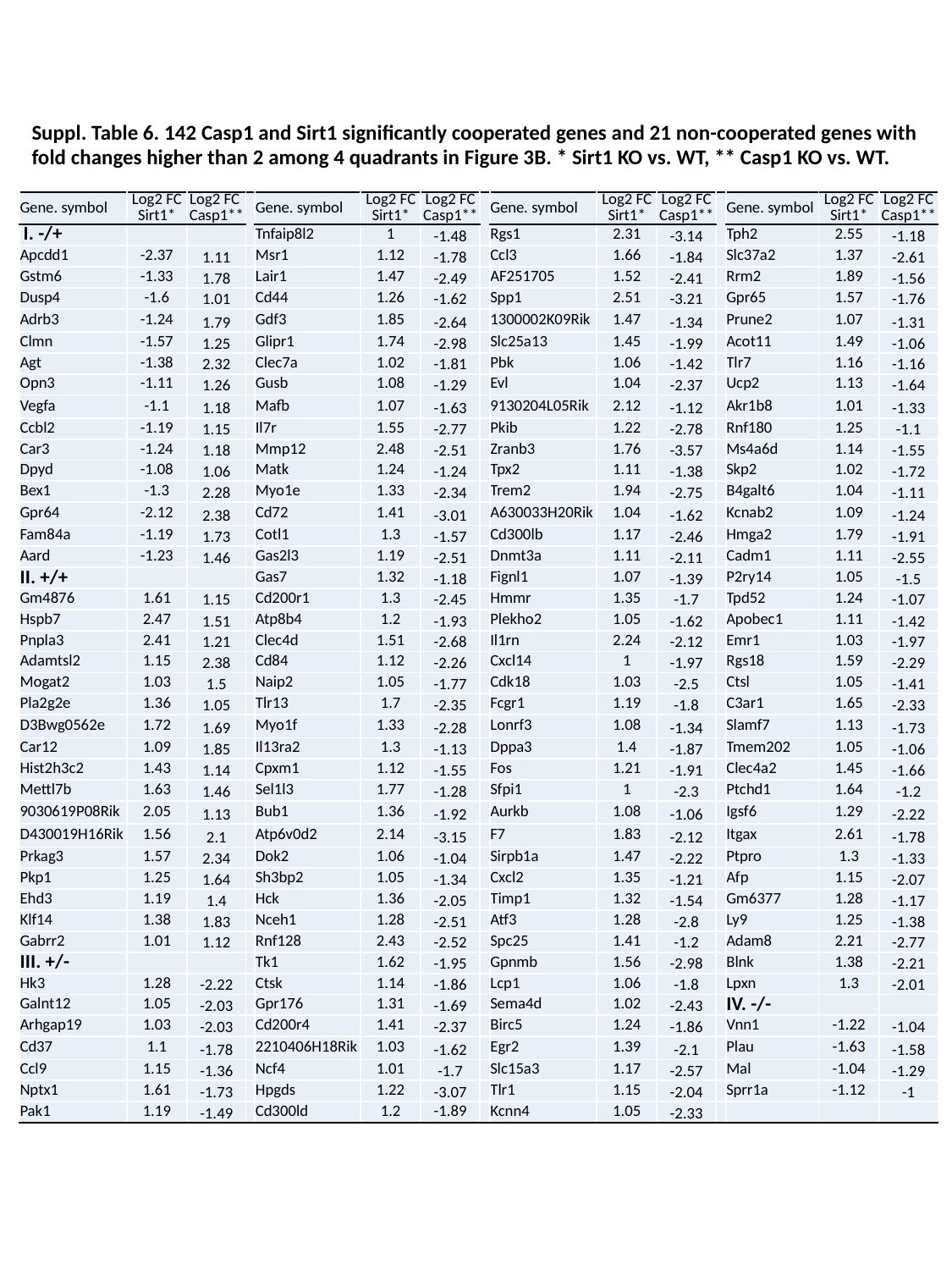

Suppl. Table 6. 142 Casp1 and Sirt1 significantly cooperated genes and 21 non-cooperated genes with fold changes higher than 2 among 4 quadrants in Figure 3B. * Sirt1 KO vs. WT, ** Casp1 KO vs. WT.
| Gene. symbol | Log2 FC Sirt1\* | Log2 FC Casp1\*\* | | Gene. symbol | Log2 FC Sirt1\* | Log2 FC Casp1\*\* | | Gene. symbol | Log2 FC Sirt1\* | Log2 FC Casp1\*\* | | Gene. symbol | Log2 FC Sirt1\* | Log2 FC Casp1\*\* |
| --- | --- | --- | --- | --- | --- | --- | --- | --- | --- | --- | --- | --- | --- | --- |
| I. -/+ | | | | Tnfaip8l2 | 1 | -1.48 | | Rgs1 | 2.31 | -3.14 | | Tph2 | 2.55 | -1.18 |
| Apcdd1 | -2.37 | 1.11 | | Msr1 | 1.12 | -1.78 | | Ccl3 | 1.66 | -1.84 | | Slc37a2 | 1.37 | -2.61 |
| Gstm6 | -1.33 | 1.78 | | Lair1 | 1.47 | -2.49 | | AF251705 | 1.52 | -2.41 | | Rrm2 | 1.89 | -1.56 |
| Dusp4 | -1.6 | 1.01 | | Cd44 | 1.26 | -1.62 | | Spp1 | 2.51 | -3.21 | | Gpr65 | 1.57 | -1.76 |
| Adrb3 | -1.24 | 1.79 | | Gdf3 | 1.85 | -2.64 | | 1300002K09Rik | 1.47 | -1.34 | | Prune2 | 1.07 | -1.31 |
| Clmn | -1.57 | 1.25 | | Glipr1 | 1.74 | -2.98 | | Slc25a13 | 1.45 | -1.99 | | Acot11 | 1.49 | -1.06 |
| Agt | -1.38 | 2.32 | | Clec7a | 1.02 | -1.81 | | Pbk | 1.06 | -1.42 | | Tlr7 | 1.16 | -1.16 |
| Opn3 | -1.11 | 1.26 | | Gusb | 1.08 | -1.29 | | Evl | 1.04 | -2.37 | | Ucp2 | 1.13 | -1.64 |
| Vegfa | -1.1 | 1.18 | | Mafb | 1.07 | -1.63 | | 9130204L05Rik | 2.12 | -1.12 | | Akr1b8 | 1.01 | -1.33 |
| Ccbl2 | -1.19 | 1.15 | | Il7r | 1.55 | -2.77 | | Pkib | 1.22 | -2.78 | | Rnf180 | 1.25 | -1.1 |
| Car3 | -1.24 | 1.18 | | Mmp12 | 2.48 | -2.51 | | Zranb3 | 1.76 | -3.57 | | Ms4a6d | 1.14 | -1.55 |
| Dpyd | -1.08 | 1.06 | | Matk | 1.24 | -1.24 | | Tpx2 | 1.11 | -1.38 | | Skp2 | 1.02 | -1.72 |
| Bex1 | -1.3 | 2.28 | | Myo1e | 1.33 | -2.34 | | Trem2 | 1.94 | -2.75 | | B4galt6 | 1.04 | -1.11 |
| Gpr64 | -2.12 | 2.38 | | Cd72 | 1.41 | -3.01 | | A630033H20Rik | 1.04 | -1.62 | | Kcnab2 | 1.09 | -1.24 |
| Fam84a | -1.19 | 1.73 | | Cotl1 | 1.3 | -1.57 | | Cd300lb | 1.17 | -2.46 | | Hmga2 | 1.79 | -1.91 |
| Aard | -1.23 | 1.46 | | Gas2l3 | 1.19 | -2.51 | | Dnmt3a | 1.11 | -2.11 | | Cadm1 | 1.11 | -2.55 |
| II. +/+ | | | | Gas7 | 1.32 | -1.18 | | Fignl1 | 1.07 | -1.39 | | P2ry14 | 1.05 | -1.5 |
| Gm4876 | 1.61 | 1.15 | | Cd200r1 | 1.3 | -2.45 | | Hmmr | 1.35 | -1.7 | | Tpd52 | 1.24 | -1.07 |
| Hspb7 | 2.47 | 1.51 | | Atp8b4 | 1.2 | -1.93 | | Plekho2 | 1.05 | -1.62 | | Apobec1 | 1.11 | -1.42 |
| Pnpla3 | 2.41 | 1.21 | | Clec4d | 1.51 | -2.68 | | Il1rn | 2.24 | -2.12 | | Emr1 | 1.03 | -1.97 |
| Adamtsl2 | 1.15 | 2.38 | | Cd84 | 1.12 | -2.26 | | Cxcl14 | 1 | -1.97 | | Rgs18 | 1.59 | -2.29 |
| Mogat2 | 1.03 | 1.5 | | Naip2 | 1.05 | -1.77 | | Cdk18 | 1.03 | -2.5 | | Ctsl | 1.05 | -1.41 |
| Pla2g2e | 1.36 | 1.05 | | Tlr13 | 1.7 | -2.35 | | Fcgr1 | 1.19 | -1.8 | | C3ar1 | 1.65 | -2.33 |
| D3Bwg0562e | 1.72 | 1.69 | | Myo1f | 1.33 | -2.28 | | Lonrf3 | 1.08 | -1.34 | | Slamf7 | 1.13 | -1.73 |
| Car12 | 1.09 | 1.85 | | Il13ra2 | 1.3 | -1.13 | | Dppa3 | 1.4 | -1.87 | | Tmem202 | 1.05 | -1.06 |
| Hist2h3c2 | 1.43 | 1.14 | | Cpxm1 | 1.12 | -1.55 | | Fos | 1.21 | -1.91 | | Clec4a2 | 1.45 | -1.66 |
| Mettl7b | 1.63 | 1.46 | | Sel1l3 | 1.77 | -1.28 | | Sfpi1 | 1 | -2.3 | | Ptchd1 | 1.64 | -1.2 |
| 9030619P08Rik | 2.05 | 1.13 | | Bub1 | 1.36 | -1.92 | | Aurkb | 1.08 | -1.06 | | Igsf6 | 1.29 | -2.22 |
| D430019H16Rik | 1.56 | 2.1 | | Atp6v0d2 | 2.14 | -3.15 | | F7 | 1.83 | -2.12 | | Itgax | 2.61 | -1.78 |
| Prkag3 | 1.57 | 2.34 | | Dok2 | 1.06 | -1.04 | | Sirpb1a | 1.47 | -2.22 | | Ptpro | 1.3 | -1.33 |
| Pkp1 | 1.25 | 1.64 | | Sh3bp2 | 1.05 | -1.34 | | Cxcl2 | 1.35 | -1.21 | | Afp | 1.15 | -2.07 |
| Ehd3 | 1.19 | 1.4 | | Hck | 1.36 | -2.05 | | Timp1 | 1.32 | -1.54 | | Gm6377 | 1.28 | -1.17 |
| Klf14 | 1.38 | 1.83 | | Nceh1 | 1.28 | -2.51 | | Atf3 | 1.28 | -2.8 | | Ly9 | 1.25 | -1.38 |
| Gabrr2 | 1.01 | 1.12 | | Rnf128 | 2.43 | -2.52 | | Spc25 | 1.41 | -1.2 | | Adam8 | 2.21 | -2.77 |
| III. +/- | | | | Tk1 | 1.62 | -1.95 | | Gpnmb | 1.56 | -2.98 | | Blnk | 1.38 | -2.21 |
| Hk3 | 1.28 | -2.22 | | Ctsk | 1.14 | -1.86 | | Lcp1 | 1.06 | -1.8 | | Lpxn | 1.3 | -2.01 |
| Galnt12 | 1.05 | -2.03 | | Gpr176 | 1.31 | -1.69 | | Sema4d | 1.02 | -2.43 | | IV. -/- | | |
| Arhgap19 | 1.03 | -2.03 | | Cd200r4 | 1.41 | -2.37 | | Birc5 | 1.24 | -1.86 | | Vnn1 | -1.22 | -1.04 |
| Cd37 | 1.1 | -1.78 | | 2210406H18Rik | 1.03 | -1.62 | | Egr2 | 1.39 | -2.1 | | Plau | -1.63 | -1.58 |
| Ccl9 | 1.15 | -1.36 | | Ncf4 | 1.01 | -1.7 | | Slc15a3 | 1.17 | -2.57 | | Mal | -1.04 | -1.29 |
| Nptx1 | 1.61 | -1.73 | | Hpgds | 1.22 | -3.07 | | Tlr1 | 1.15 | -2.04 | | Sprr1a | -1.12 | -1 |
| Pak1 | 1.19 | -1.49 | | Cd300ld | 1.2 | -1.89 | | Kcnn4 | 1.05 | -2.33 | | | | |

## Slide 9
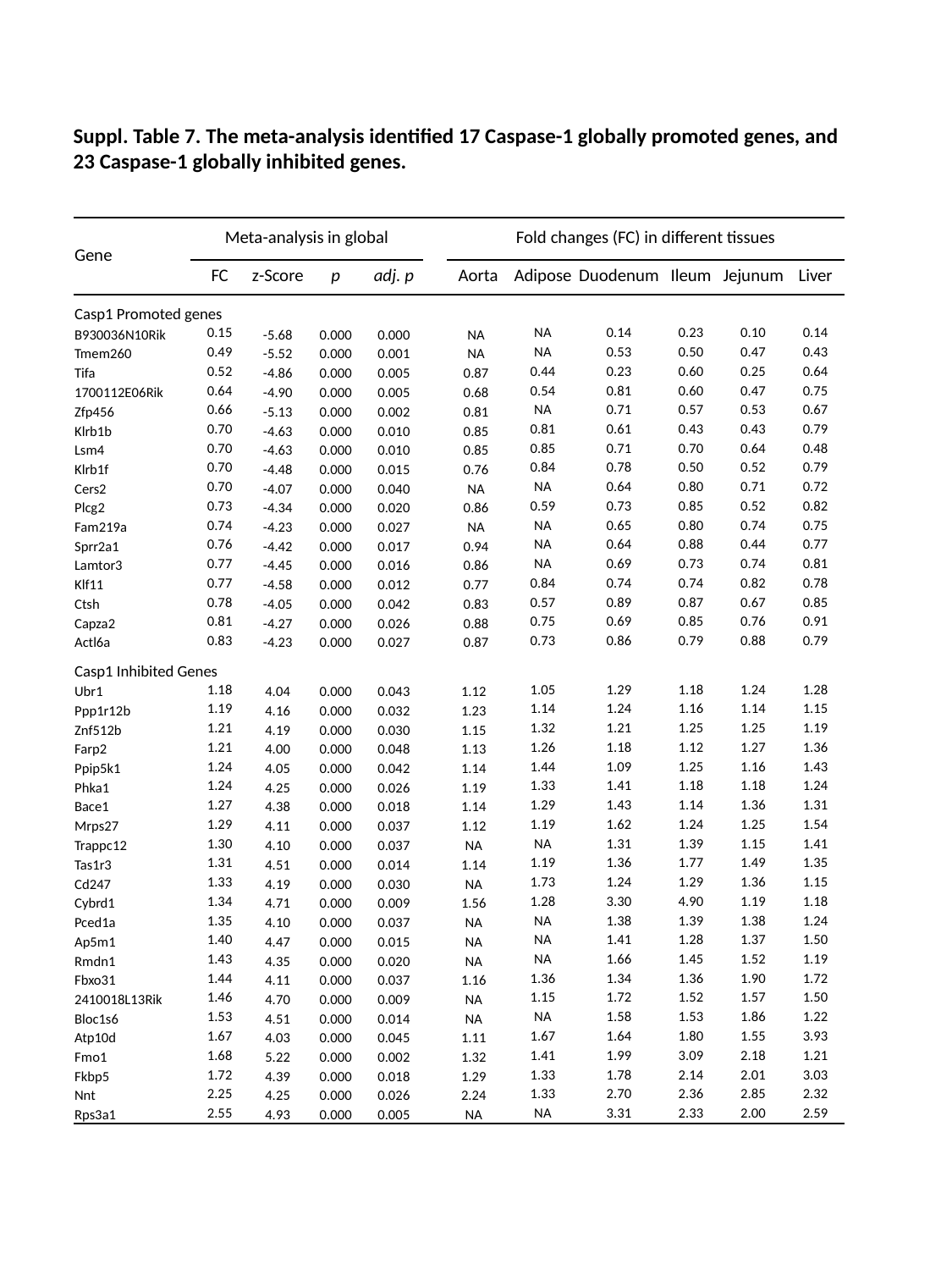

Suppl. Table 7. The meta-analysis identified 17 Caspase-1 globally promoted genes, and 23 Caspase-1 globally inhibited genes.
| Gene | Meta-analysis in global | | | | | | | Fold changes (FC) in different tissues | | | | | |
| --- | --- | --- | --- | --- | --- | --- | --- | --- | --- | --- | --- | --- | --- |
| | FC | z-Score | p | adj. p | | | | Aorta | Adipose | Duodenum | Ileum | Jejunum | Liver |
| Casp1 Promoted genes | | | | | | | | | | | | | |
| B930036N10Rik | 0.15 | -5.68 | 0.000 | 0.000 | | | NA | | NA | 0.14 | 0.23 | 0.10 | 0.14 |
| Tmem260 | 0.49 | -5.52 | 0.000 | 0.001 | | | NA | | NA | 0.53 | 0.50 | 0.47 | 0.43 |
| Tifa | 0.52 | -4.86 | 0.000 | 0.005 | | | 0.87 | | 0.44 | 0.23 | 0.60 | 0.25 | 0.64 |
| 1700112E06Rik | 0.64 | -4.90 | 0.000 | 0.005 | | | 0.68 | | 0.54 | 0.81 | 0.60 | 0.47 | 0.75 |
| Zfp456 | 0.66 | -5.13 | 0.000 | 0.002 | | | 0.81 | | NA | 0.71 | 0.57 | 0.53 | 0.67 |
| Klrb1b | 0.70 | -4.63 | 0.000 | 0.010 | | | 0.85 | | 0.81 | 0.61 | 0.43 | 0.43 | 0.79 |
| Lsm4 | 0.70 | -4.63 | 0.000 | 0.010 | | | 0.85 | | 0.85 | 0.71 | 0.70 | 0.64 | 0.48 |
| Klrb1f | 0.70 | -4.48 | 0.000 | 0.015 | | | 0.76 | | 0.84 | 0.78 | 0.50 | 0.52 | 0.79 |
| Cers2 | 0.70 | -4.07 | 0.000 | 0.040 | | | NA | | NA | 0.64 | 0.80 | 0.71 | 0.72 |
| Plcg2 | 0.73 | -4.34 | 0.000 | 0.020 | | | 0.86 | | 0.59 | 0.73 | 0.85 | 0.52 | 0.82 |
| Fam219a | 0.74 | -4.23 | 0.000 | 0.027 | | | NA | | NA | 0.65 | 0.80 | 0.74 | 0.75 |
| Sprr2a1 | 0.76 | -4.42 | 0.000 | 0.017 | | | 0.94 | | NA | 0.64 | 0.88 | 0.44 | 0.77 |
| Lamtor3 | 0.77 | -4.45 | 0.000 | 0.016 | | | 0.86 | | NA | 0.69 | 0.73 | 0.74 | 0.81 |
| Klf11 | 0.77 | -4.58 | 0.000 | 0.012 | | | 0.77 | | 0.84 | 0.74 | 0.74 | 0.82 | 0.78 |
| Ctsh | 0.78 | -4.05 | 0.000 | 0.042 | | | 0.83 | | 0.57 | 0.89 | 0.87 | 0.67 | 0.85 |
| Capza2 | 0.81 | -4.27 | 0.000 | 0.026 | | | 0.88 | | 0.75 | 0.69 | 0.85 | 0.76 | 0.91 |
| Actl6a | 0.83 | -4.23 | 0.000 | 0.027 | | | 0.87 | | 0.73 | 0.86 | 0.79 | 0.88 | 0.79 |
| Casp1 Inhibited Genes | | | | | | | | | | | | | |
| Ubr1 | 1.18 | 4.04 | 0.000 | 0.043 | | 1.12 | | | 1.05 | 1.29 | 1.18 | 1.24 | 1.28 |
| Ppp1r12b | 1.19 | 4.16 | 0.000 | 0.032 | | 1.23 | | | 1.14 | 1.24 | 1.16 | 1.14 | 1.15 |
| Znf512b | 1.21 | 4.19 | 0.000 | 0.030 | | 1.15 | | | 1.32 | 1.21 | 1.25 | 1.25 | 1.19 |
| Farp2 | 1.21 | 4.00 | 0.000 | 0.048 | | 1.13 | | | 1.26 | 1.18 | 1.12 | 1.27 | 1.36 |
| Ppip5k1 | 1.24 | 4.05 | 0.000 | 0.042 | | 1.14 | | | 1.44 | 1.09 | 1.25 | 1.16 | 1.43 |
| Phka1 | 1.24 | 4.25 | 0.000 | 0.026 | | 1.19 | | | 1.33 | 1.41 | 1.18 | 1.18 | 1.24 |
| Bace1 | 1.27 | 4.38 | 0.000 | 0.018 | | 1.14 | | | 1.29 | 1.43 | 1.14 | 1.36 | 1.31 |
| Mrps27 | 1.29 | 4.11 | 0.000 | 0.037 | | 1.12 | | | 1.19 | 1.62 | 1.24 | 1.25 | 1.54 |
| Trappc12 | 1.30 | 4.10 | 0.000 | 0.037 | | NA | | | NA | 1.31 | 1.39 | 1.15 | 1.41 |
| Tas1r3 | 1.31 | 4.51 | 0.000 | 0.014 | | 1.14 | | | 1.19 | 1.36 | 1.77 | 1.49 | 1.35 |
| Cd247 | 1.33 | 4.19 | 0.000 | 0.030 | | NA | | | 1.73 | 1.24 | 1.29 | 1.36 | 1.15 |
| Cybrd1 | 1.34 | 4.71 | 0.000 | 0.009 | | 1.56 | | | 1.28 | 3.30 | 4.90 | 1.19 | 1.18 |
| Pced1a | 1.35 | 4.10 | 0.000 | 0.037 | | NA | | | NA | 1.38 | 1.39 | 1.38 | 1.24 |
| Ap5m1 | 1.40 | 4.47 | 0.000 | 0.015 | | NA | | | NA | 1.41 | 1.28 | 1.37 | 1.50 |
| Rmdn1 | 1.43 | 4.35 | 0.000 | 0.020 | | NA | | | NA | 1.66 | 1.45 | 1.52 | 1.19 |
| Fbxo31 | 1.44 | 4.11 | 0.000 | 0.037 | | 1.16 | | | 1.36 | 1.34 | 1.36 | 1.90 | 1.72 |
| 2410018L13Rik | 1.46 | 4.70 | 0.000 | 0.009 | | NA | | | 1.15 | 1.72 | 1.52 | 1.57 | 1.50 |
| Bloc1s6 | 1.53 | 4.51 | 0.000 | 0.014 | | NA | | | NA | 1.58 | 1.53 | 1.86 | 1.22 |
| Atp10d | 1.67 | 4.03 | 0.000 | 0.045 | | 1.11 | | | 1.67 | 1.64 | 1.80 | 1.55 | 3.93 |
| Fmo1 | 1.68 | 5.22 | 0.000 | 0.002 | | 1.32 | | | 1.41 | 1.99 | 3.09 | 2.18 | 1.21 |
| Fkbp5 | 1.72 | 4.39 | 0.000 | 0.018 | | 1.29 | | | 1.33 | 1.78 | 2.14 | 2.01 | 3.03 |
| Nnt | 2.25 | 4.25 | 0.000 | 0.026 | | 2.24 | | | 1.33 | 2.70 | 2.36 | 2.85 | 2.32 |
| Rps3a1 | 2.55 | 4.93 | 0.000 | 0.005 | | NA | | | NA | 3.31 | 2.33 | 2.00 | 2.59 |

## Slide 10
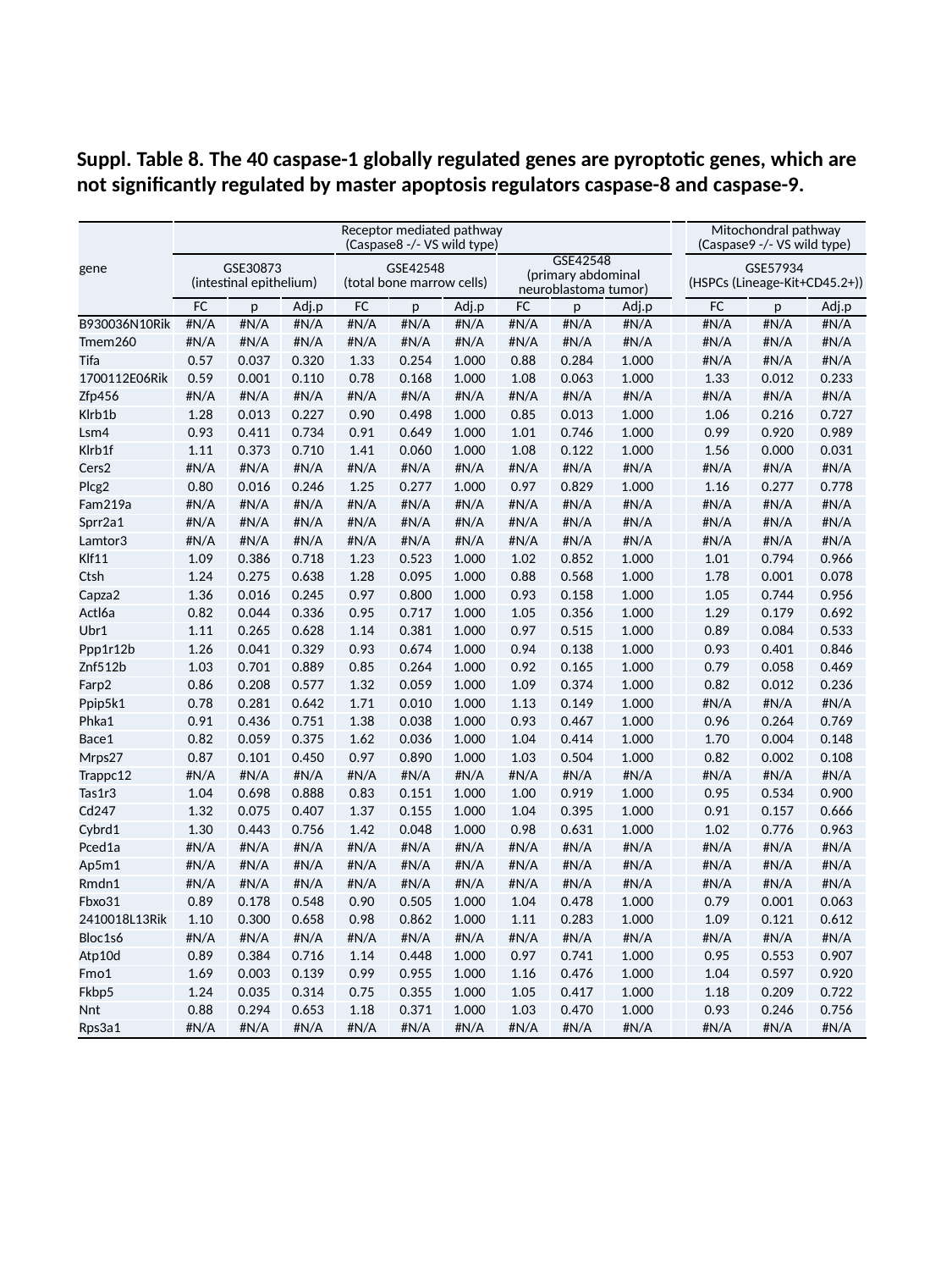

Suppl. Table 8. The 40 caspase-1 globally regulated genes are pyroptotic genes, which are not significantly regulated by master apoptosis regulators caspase-8 and caspase-9.
| gene | Receptor mediated pathway (Caspase8 -/- VS wild type) | | | | | | | | | | Mitochondral pathway (Caspase9 -/- VS wild type) | | |
| --- | --- | --- | --- | --- | --- | --- | --- | --- | --- | --- | --- | --- | --- |
| | GSE30873 (intestinal epithelium) | | | GSE42548 (total bone marrow cells) | | | GSE42548 (primary abdominal neuroblastoma tumor) | | | | GSE57934 (HSPCs (Lineage-Kit+CD45.2+)) | | |
| | FC | p | Adj.p | FC | p | Adj.p | FC | p | Adj.p | | FC | p | Adj.p |
| B930036N10Rik | #N/A | #N/A | #N/A | #N/A | #N/A | #N/A | #N/A | #N/A | #N/A | | #N/A | #N/A | #N/A |
| Tmem260 | #N/A | #N/A | #N/A | #N/A | #N/A | #N/A | #N/A | #N/A | #N/A | | #N/A | #N/A | #N/A |
| Tifa | 0.57 | 0.037 | 0.320 | 1.33 | 0.254 | 1.000 | 0.88 | 0.284 | 1.000 | | #N/A | #N/A | #N/A |
| 1700112E06Rik | 0.59 | 0.001 | 0.110 | 0.78 | 0.168 | 1.000 | 1.08 | 0.063 | 1.000 | | 1.33 | 0.012 | 0.233 |
| Zfp456 | #N/A | #N/A | #N/A | #N/A | #N/A | #N/A | #N/A | #N/A | #N/A | | #N/A | #N/A | #N/A |
| Klrb1b | 1.28 | 0.013 | 0.227 | 0.90 | 0.498 | 1.000 | 0.85 | 0.013 | 1.000 | | 1.06 | 0.216 | 0.727 |
| Lsm4 | 0.93 | 0.411 | 0.734 | 0.91 | 0.649 | 1.000 | 1.01 | 0.746 | 1.000 | | 0.99 | 0.920 | 0.989 |
| Klrb1f | 1.11 | 0.373 | 0.710 | 1.41 | 0.060 | 1.000 | 1.08 | 0.122 | 1.000 | | 1.56 | 0.000 | 0.031 |
| Cers2 | #N/A | #N/A | #N/A | #N/A | #N/A | #N/A | #N/A | #N/A | #N/A | | #N/A | #N/A | #N/A |
| Plcg2 | 0.80 | 0.016 | 0.246 | 1.25 | 0.277 | 1.000 | 0.97 | 0.829 | 1.000 | | 1.16 | 0.277 | 0.778 |
| Fam219a | #N/A | #N/A | #N/A | #N/A | #N/A | #N/A | #N/A | #N/A | #N/A | | #N/A | #N/A | #N/A |
| Sprr2a1 | #N/A | #N/A | #N/A | #N/A | #N/A | #N/A | #N/A | #N/A | #N/A | | #N/A | #N/A | #N/A |
| Lamtor3 | #N/A | #N/A | #N/A | #N/A | #N/A | #N/A | #N/A | #N/A | #N/A | | #N/A | #N/A | #N/A |
| Klf11 | 1.09 | 0.386 | 0.718 | 1.23 | 0.523 | 1.000 | 1.02 | 0.852 | 1.000 | | 1.01 | 0.794 | 0.966 |
| Ctsh | 1.24 | 0.275 | 0.638 | 1.28 | 0.095 | 1.000 | 0.88 | 0.568 | 1.000 | | 1.78 | 0.001 | 0.078 |
| Capza2 | 1.36 | 0.016 | 0.245 | 0.97 | 0.800 | 1.000 | 0.93 | 0.158 | 1.000 | | 1.05 | 0.744 | 0.956 |
| Actl6a | 0.82 | 0.044 | 0.336 | 0.95 | 0.717 | 1.000 | 1.05 | 0.356 | 1.000 | | 1.29 | 0.179 | 0.692 |
| Ubr1 | 1.11 | 0.265 | 0.628 | 1.14 | 0.381 | 1.000 | 0.97 | 0.515 | 1.000 | | 0.89 | 0.084 | 0.533 |
| Ppp1r12b | 1.26 | 0.041 | 0.329 | 0.93 | 0.674 | 1.000 | 0.94 | 0.138 | 1.000 | | 0.93 | 0.401 | 0.846 |
| Znf512b | 1.03 | 0.701 | 0.889 | 0.85 | 0.264 | 1.000 | 0.92 | 0.165 | 1.000 | | 0.79 | 0.058 | 0.469 |
| Farp2 | 0.86 | 0.208 | 0.577 | 1.32 | 0.059 | 1.000 | 1.09 | 0.374 | 1.000 | | 0.82 | 0.012 | 0.236 |
| Ppip5k1 | 0.78 | 0.281 | 0.642 | 1.71 | 0.010 | 1.000 | 1.13 | 0.149 | 1.000 | | #N/A | #N/A | #N/A |
| Phka1 | 0.91 | 0.436 | 0.751 | 1.38 | 0.038 | 1.000 | 0.93 | 0.467 | 1.000 | | 0.96 | 0.264 | 0.769 |
| Bace1 | 0.82 | 0.059 | 0.375 | 1.62 | 0.036 | 1.000 | 1.04 | 0.414 | 1.000 | | 1.70 | 0.004 | 0.148 |
| Mrps27 | 0.87 | 0.101 | 0.450 | 0.97 | 0.890 | 1.000 | 1.03 | 0.504 | 1.000 | | 0.82 | 0.002 | 0.108 |
| Trappc12 | #N/A | #N/A | #N/A | #N/A | #N/A | #N/A | #N/A | #N/A | #N/A | | #N/A | #N/A | #N/A |
| Tas1r3 | 1.04 | 0.698 | 0.888 | 0.83 | 0.151 | 1.000 | 1.00 | 0.919 | 1.000 | | 0.95 | 0.534 | 0.900 |
| Cd247 | 1.32 | 0.075 | 0.407 | 1.37 | 0.155 | 1.000 | 1.04 | 0.395 | 1.000 | | 0.91 | 0.157 | 0.666 |
| Cybrd1 | 1.30 | 0.443 | 0.756 | 1.42 | 0.048 | 1.000 | 0.98 | 0.631 | 1.000 | | 1.02 | 0.776 | 0.963 |
| Pced1a | #N/A | #N/A | #N/A | #N/A | #N/A | #N/A | #N/A | #N/A | #N/A | | #N/A | #N/A | #N/A |
| Ap5m1 | #N/A | #N/A | #N/A | #N/A | #N/A | #N/A | #N/A | #N/A | #N/A | | #N/A | #N/A | #N/A |
| Rmdn1 | #N/A | #N/A | #N/A | #N/A | #N/A | #N/A | #N/A | #N/A | #N/A | | #N/A | #N/A | #N/A |
| Fbxo31 | 0.89 | 0.178 | 0.548 | 0.90 | 0.505 | 1.000 | 1.04 | 0.478 | 1.000 | | 0.79 | 0.001 | 0.063 |
| 2410018L13Rik | 1.10 | 0.300 | 0.658 | 0.98 | 0.862 | 1.000 | 1.11 | 0.283 | 1.000 | | 1.09 | 0.121 | 0.612 |
| Bloc1s6 | #N/A | #N/A | #N/A | #N/A | #N/A | #N/A | #N/A | #N/A | #N/A | | #N/A | #N/A | #N/A |
| Atp10d | 0.89 | 0.384 | 0.716 | 1.14 | 0.448 | 1.000 | 0.97 | 0.741 | 1.000 | | 0.95 | 0.553 | 0.907 |
| Fmo1 | 1.69 | 0.003 | 0.139 | 0.99 | 0.955 | 1.000 | 1.16 | 0.476 | 1.000 | | 1.04 | 0.597 | 0.920 |
| Fkbp5 | 1.24 | 0.035 | 0.314 | 0.75 | 0.355 | 1.000 | 1.05 | 0.417 | 1.000 | | 1.18 | 0.209 | 0.722 |
| Nnt | 0.88 | 0.294 | 0.653 | 1.18 | 0.371 | 1.000 | 1.03 | 0.470 | 1.000 | | 0.93 | 0.246 | 0.756 |
| Rps3a1 | #N/A | #N/A | #N/A | #N/A | #N/A | #N/A | #N/A | #N/A | #N/A | | #N/A | #N/A | #N/A |

## Slide 11
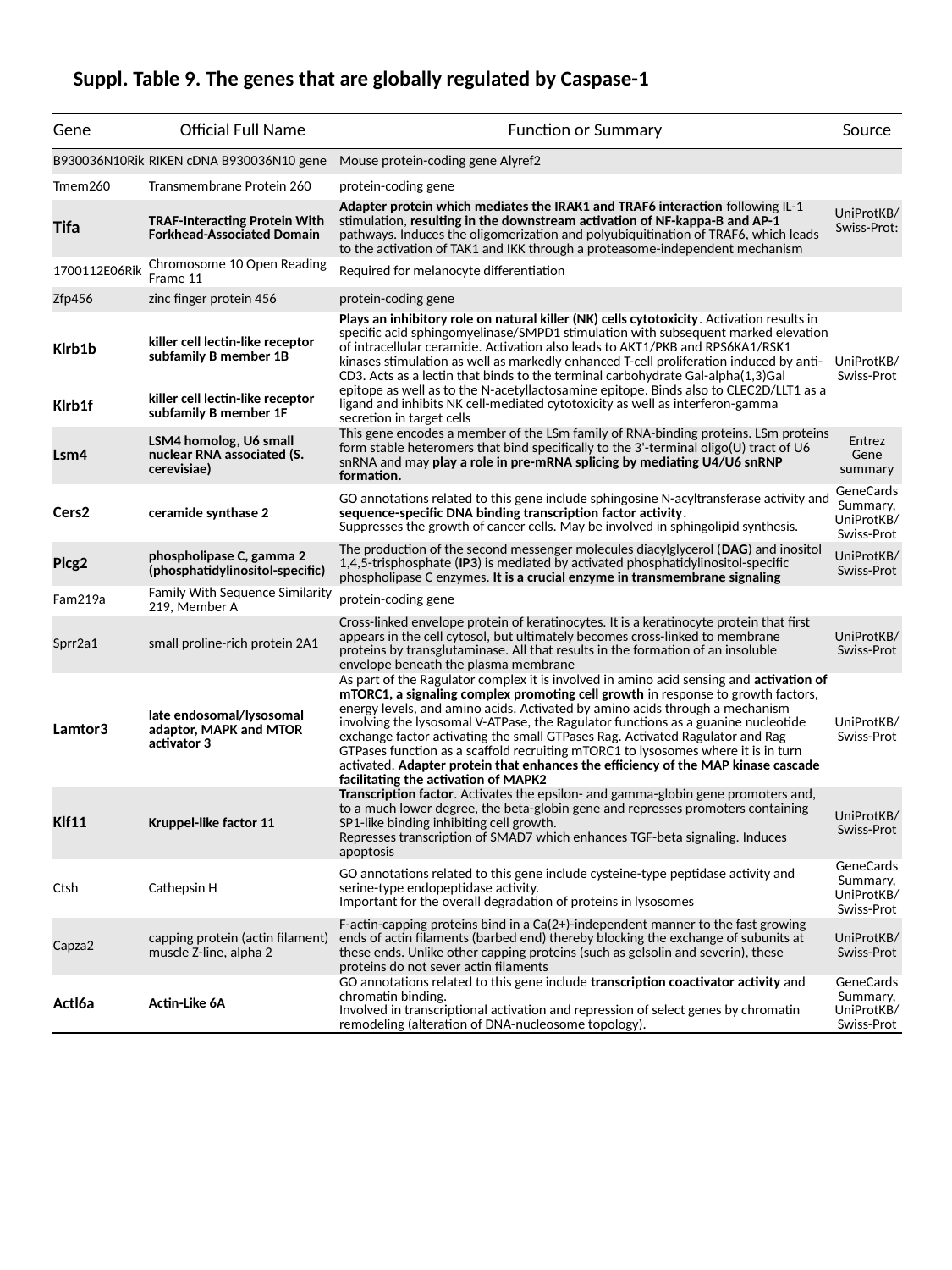

Suppl. Table 9. The genes that are globally regulated by Caspase-1
| Gene | Official Full Name | Function or Summary | Source |
| --- | --- | --- | --- |
| B930036N10Rik | RIKEN cDNA B930036N10 gene | Mouse protein-coding gene Alyref2 | |
| Tmem260 | Transmembrane Protein 260 | protein-coding gene | |
| Tifa | TRAF-Interacting Protein With Forkhead-Associated Domain | Adapter protein which mediates the IRAK1 and TRAF6 interaction following IL-1 stimulation, resulting in the downstream activation of NF-kappa-B and AP-1 pathways. Induces the oligomerization and polyubiquitination of TRAF6, which leads to the activation of TAK1 and IKK through a proteasome-independent mechanism | UniProtKB/Swiss-Prot: |
| 1700112E06Rik | Chromosome 10 Open Reading Frame 11 | Required for melanocyte differentiation | |
| Zfp456 | zinc finger protein 456 | protein-coding gene | |
| Klrb1b | killer cell lectin-like receptor subfamily B member 1B | Plays an inhibitory role on natural killer (NK) cells cytotoxicity. Activation results in specific acid sphingomyelinase/SMPD1 stimulation with subsequent marked elevation of intracellular ceramide. Activation also leads to AKT1/PKB and RPS6KA1/RSK1 kinases stimulation as well as markedly enhanced T-cell proliferation induced by anti-CD3. Acts as a lectin that binds to the terminal carbohydrate Gal-alpha(1,3)Gal epitope as well as to the N-acetyllactosamine epitope. Binds also to CLEC2D/LLT1 as a ligand and inhibits NK cell-mediated cytotoxicity as well as interferon-gamma secretion in target cells | UniProtKB/Swiss-Prot |
| Klrb1f | killer cell lectin-like receptor subfamily B member 1F | | |
| Lsm4 | LSM4 homolog, U6 small nuclear RNA associated (S. cerevisiae) | This gene encodes a member of the LSm family of RNA-binding proteins. LSm proteins form stable heteromers that bind specifically to the 3'-terminal oligo(U) tract of U6 snRNA and may play a role in pre-mRNA splicing by mediating U4/U6 snRNP formation. | Entrez Gene summary |
| Cers2 | ceramide synthase 2 | GO annotations related to this gene include sphingosine N-acyltransferase activity and sequence-specific DNA binding transcription factor activity. Suppresses the growth of cancer cells. May be involved in sphingolipid synthesis. | GeneCards Summary, UniProtKB/Swiss-Prot |
| Plcg2 | phospholipase C, gamma 2 (phosphatidylinositol-specific) | The production of the second messenger molecules diacylglycerol (DAG) and inositol 1,4,5-trisphosphate (IP3) is mediated by activated phosphatidylinositol-specific phospholipase C enzymes. It is a crucial enzyme in transmembrane signaling | UniProtKB/Swiss-Prot |
| Fam219a | Family With Sequence Similarity 219, Member A | protein-coding gene | |
| Sprr2a1 | small proline-rich protein 2A1 | Cross-linked envelope protein of keratinocytes. It is a keratinocyte protein that first appears in the cell cytosol, but ultimately becomes cross-linked to membrane proteins by transglutaminase. All that results in the formation of an insoluble envelope beneath the plasma membrane | UniProtKB/Swiss-Prot |
| Lamtor3 | late endosomal/lysosomal adaptor, MAPK and MTOR activator 3 | As part of the Ragulator complex it is involved in amino acid sensing and activation of mTORC1, a signaling complex promoting cell growth in response to growth factors, energy levels, and amino acids. Activated by amino acids through a mechanism involving the lysosomal V-ATPase, the Ragulator functions as a guanine nucleotide exchange factor activating the small GTPases Rag. Activated Ragulator and Rag GTPases function as a scaffold recruiting mTORC1 to lysosomes where it is in turn activated. Adapter protein that enhances the efficiency of the MAP kinase cascade facilitating the activation of MAPK2 | UniProtKB/Swiss-Prot |
| Klf11 | Kruppel-like factor 11 | Transcription factor. Activates the epsilon- and gamma-globin gene promoters and, to a much lower degree, the beta-globin gene and represses promoters containing SP1-like binding inhibiting cell growth. Represses transcription of SMAD7 which enhances TGF-beta signaling. Induces apoptosis | UniProtKB/Swiss-Prot |
| Ctsh | Cathepsin H | GO annotations related to this gene include cysteine-type peptidase activity and serine-type endopeptidase activity. Important for the overall degradation of proteins in lysosomes | GeneCards Summary, UniProtKB/Swiss-Prot |
| Capza2 | capping protein (actin filament) muscle Z-line, alpha 2 | F-actin-capping proteins bind in a Ca(2+)-independent manner to the fast growing ends of actin filaments (barbed end) thereby blocking the exchange of subunits at these ends. Unlike other capping proteins (such as gelsolin and severin), these proteins do not sever actin filaments | UniProtKB/Swiss-Prot |
| Actl6a | Actin-Like 6A | GO annotations related to this gene include transcription coactivator activity and chromatin binding. Involved in transcriptional activation and repression of select genes by chromatin remodeling (alteration of DNA-nucleosome topology). | GeneCards Summary, UniProtKB/Swiss-Prot |

## Slide 12
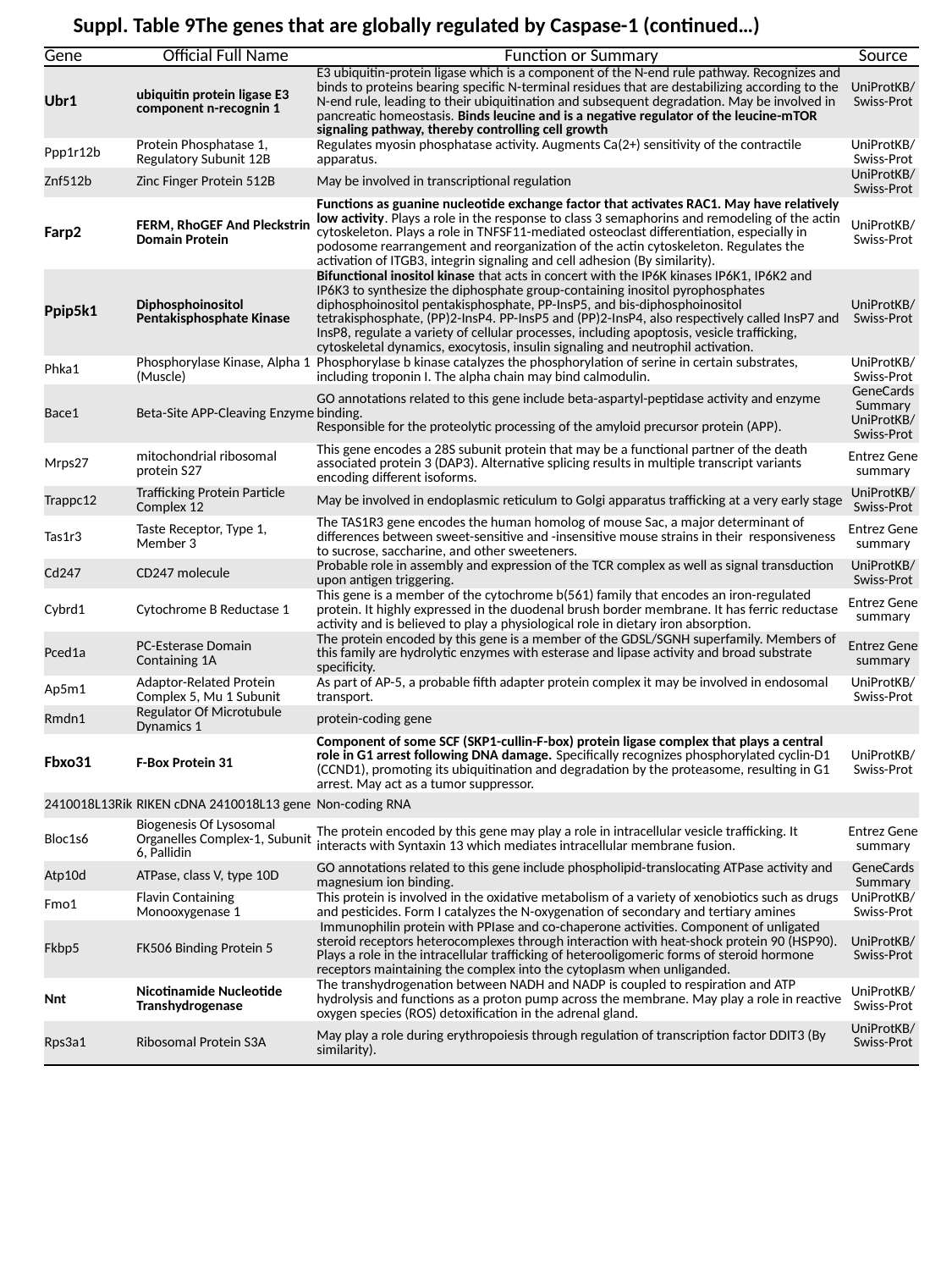

Suppl. Table 9The genes that are globally regulated by Caspase-1 (continued…)
| Gene | Official Full Name | Function or Summary | Source |
| --- | --- | --- | --- |
| Ubr1 | ubiquitin protein ligase E3 component n-recognin 1 | E3 ubiquitin-protein ligase which is a component of the N-end rule pathway. Recognizes and binds to proteins bearing specific N-terminal residues that are destabilizing according to the N-end rule, leading to their ubiquitination and subsequent degradation. May be involved in pancreatic homeostasis. Binds leucine and is a negative regulator of the leucine-mTOR signaling pathway, thereby controlling cell growth | UniProtKB/Swiss-Prot |
| Ppp1r12b | Protein Phosphatase 1, Regulatory Subunit 12B | Regulates myosin phosphatase activity. Augments Ca(2+) sensitivity of the contractile apparatus. | UniProtKB/Swiss-Prot |
| Znf512b | Zinc Finger Protein 512B | May be involved in transcriptional regulation | UniProtKB/Swiss-Prot |
| Farp2 | FERM, RhoGEF And Pleckstrin Domain Protein | Functions as guanine nucleotide exchange factor that activates RAC1. May have relatively low activity. Plays a role in the response to class 3 semaphorins and remodeling of the actin cytoskeleton. Plays a role in TNFSF11-mediated osteoclast differentiation, especially in podosome rearrangement and reorganization of the actin cytoskeleton. Regulates the activation of ITGB3, integrin signaling and cell adhesion (By similarity). | UniProtKB/Swiss-Prot |
| Ppip5k1 | Diphosphoinositol Pentakisphosphate Kinase | Bifunctional inositol kinase that acts in concert with the IP6K kinases IP6K1, IP6K2 and IP6K3 to synthesize the diphosphate group-containing inositol pyrophosphates diphosphoinositol pentakisphosphate, PP-InsP5, and bis-diphosphoinositol tetrakisphosphate, (PP)2-InsP4. PP-InsP5 and (PP)2-InsP4, also respectively called InsP7 and InsP8, regulate a variety of cellular processes, including apoptosis, vesicle trafficking, cytoskeletal dynamics, exocytosis, insulin signaling and neutrophil activation. | UniProtKB/Swiss-Prot |
| Phka1 | Phosphorylase Kinase, Alpha 1 (Muscle) | Phosphorylase b kinase catalyzes the phosphorylation of serine in certain substrates, including troponin I. The alpha chain may bind calmodulin. | UniProtKB/Swiss-Prot |
| Bace1 | Beta-Site APP-Cleaving Enzyme | GO annotations related to this gene include beta-aspartyl-peptidase activity and enzyme binding. Responsible for the proteolytic processing of the amyloid precursor protein (APP). | GeneCards Summary UniProtKB/Swiss-Prot |
| Mrps27 | mitochondrial ribosomal protein S27 | This gene encodes a 28S subunit protein that may be a functional partner of the death associated protein 3 (DAP3). Alternative splicing results in multiple transcript variants encoding different isoforms. | Entrez Gene summary |
| Trappc12 | Trafficking Protein Particle Complex 12 | May be involved in endoplasmic reticulum to Golgi apparatus trafficking at a very early stage | UniProtKB/Swiss-Prot |
| Tas1r3 | Taste Receptor, Type 1, Member 3 | The TAS1R3 gene encodes the human homolog of mouse Sac, a major determinant of differences between sweet-sensitive and -insensitive mouse strains in their responsiveness to sucrose, saccharine, and other sweeteners. | Entrez Gene summary |
| Cd247 | CD247 molecule | Probable role in assembly and expression of the TCR complex as well as signal transduction upon antigen triggering. | UniProtKB/Swiss-Prot |
| Cybrd1 | Cytochrome B Reductase 1 | This gene is a member of the cytochrome b(561) family that encodes an iron-regulated protein. It highly expressed in the duodenal brush border membrane. It has ferric reductase activity and is believed to play a physiological role in dietary iron absorption. | Entrez Gene summary |
| Pced1a | PC-Esterase Domain Containing 1A | The protein encoded by this gene is a member of the GDSL/SGNH superfamily. Members of this family are hydrolytic enzymes with esterase and lipase activity and broad substrate specificity. | Entrez Gene summary |
| Ap5m1 | Adaptor-Related Protein Complex 5, Mu 1 Subunit | As part of AP-5, a probable fifth adapter protein complex it may be involved in endosomal transport. | UniProtKB/Swiss-Prot |
| Rmdn1 | Regulator Of Microtubule Dynamics 1 | protein-coding gene | |
| Fbxo31 | F-Box Protein 31 | Component of some SCF (SKP1-cullin-F-box) protein ligase complex that plays a central role in G1 arrest following DNA damage. Specifically recognizes phosphorylated cyclin-D1 (CCND1), promoting its ubiquitination and degradation by the proteasome, resulting in G1 arrest. May act as a tumor suppressor. | UniProtKB/Swiss-Prot |
| 2410018L13Rik | RIKEN cDNA 2410018L13 gene | Non-coding RNA | |
| Bloc1s6 | Biogenesis Of Lysosomal Organelles Complex-1, Subunit 6, Pallidin | The protein encoded by this gene may play a role in intracellular vesicle trafficking. It interacts with Syntaxin 13 which mediates intracellular membrane fusion. | Entrez Gene summary |
| Atp10d | ATPase, class V, type 10D | GO annotations related to this gene include phospholipid-translocating ATPase activity and magnesium ion binding. | GeneCards Summary |
| Fmo1 | Flavin Containing Monooxygenase 1 | This protein is involved in the oxidative metabolism of a variety of xenobiotics such as drugs and pesticides. Form I catalyzes the N-oxygenation of secondary and tertiary amines | UniProtKB/Swiss-Prot |
| Fkbp5 | FK506 Binding Protein 5 | Immunophilin protein with PPIase and co-chaperone activities. Component of unligated steroid receptors heterocomplexes through interaction with heat-shock protein 90 (HSP90). Plays a role in the intracellular trafficking of heterooligomeric forms of steroid hormone receptors maintaining the complex into the cytoplasm when unliganded. | UniProtKB/Swiss-Prot |
| Nnt | Nicotinamide Nucleotide Transhydrogenase | The transhydrogenation between NADH and NADP is coupled to respiration and ATP hydrolysis and functions as a proton pump across the membrane. May play a role in reactive oxygen species (ROS) detoxification in the adrenal gland. | UniProtKB/Swiss-Prot |
| Rps3a1 | Ribosomal Protein S3A | May play a role during erythropoiesis through regulation of transcription factor DDIT3 (By similarity). | UniProtKB/Swiss-Prot |

## Slide 13
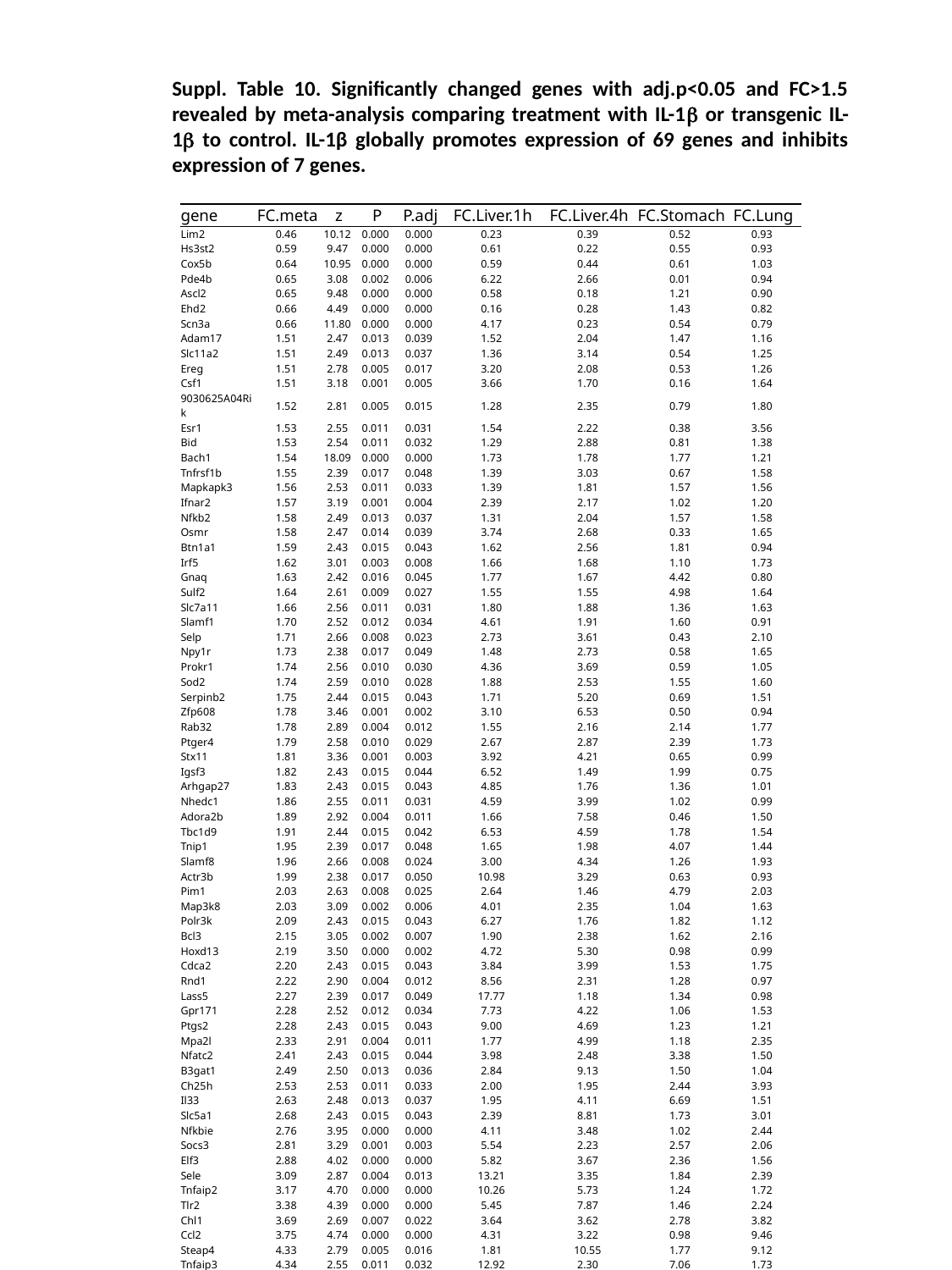

Suppl. Table 10. Significantly changed genes with adj.p<0.05 and FC>1.5 revealed by meta-analysis comparing treatment with IL-1b or transgenic IL-1b to control. IL-1β globally promotes expression of 69 genes and inhibits expression of 7 genes.
| gene | FC.meta | z | P | P.adj | FC.Liver.1h | FC.Liver.4h | FC.Stomach | FC.Lung |
| --- | --- | --- | --- | --- | --- | --- | --- | --- |
| Lim2 | 0.46 | 10.12 | 0.000 | 0.000 | 0.23 | 0.39 | 0.52 | 0.93 |
| Hs3st2 | 0.59 | 9.47 | 0.000 | 0.000 | 0.61 | 0.22 | 0.55 | 0.93 |
| Cox5b | 0.64 | 10.95 | 0.000 | 0.000 | 0.59 | 0.44 | 0.61 | 1.03 |
| Pde4b | 0.65 | 3.08 | 0.002 | 0.006 | 6.22 | 2.66 | 0.01 | 0.94 |
| Ascl2 | 0.65 | 9.48 | 0.000 | 0.000 | 0.58 | 0.18 | 1.21 | 0.90 |
| Ehd2 | 0.66 | 4.49 | 0.000 | 0.000 | 0.16 | 0.28 | 1.43 | 0.82 |
| Scn3a | 0.66 | 11.80 | 0.000 | 0.000 | 4.17 | 0.23 | 0.54 | 0.79 |
| Adam17 | 1.51 | 2.47 | 0.013 | 0.039 | 1.52 | 2.04 | 1.47 | 1.16 |
| Slc11a2 | 1.51 | 2.49 | 0.013 | 0.037 | 1.36 | 3.14 | 0.54 | 1.25 |
| Ereg | 1.51 | 2.78 | 0.005 | 0.017 | 3.20 | 2.08 | 0.53 | 1.26 |
| Csf1 | 1.51 | 3.18 | 0.001 | 0.005 | 3.66 | 1.70 | 0.16 | 1.64 |
| 9030625A04Rik | 1.52 | 2.81 | 0.005 | 0.015 | 1.28 | 2.35 | 0.79 | 1.80 |
| Esr1 | 1.53 | 2.55 | 0.011 | 0.031 | 1.54 | 2.22 | 0.38 | 3.56 |
| Bid | 1.53 | 2.54 | 0.011 | 0.032 | 1.29 | 2.88 | 0.81 | 1.38 |
| Bach1 | 1.54 | 18.09 | 0.000 | 0.000 | 1.73 | 1.78 | 1.77 | 1.21 |
| Tnfrsf1b | 1.55 | 2.39 | 0.017 | 0.048 | 1.39 | 3.03 | 0.67 | 1.58 |
| Mapkapk3 | 1.56 | 2.53 | 0.011 | 0.033 | 1.39 | 1.81 | 1.57 | 1.56 |
| Ifnar2 | 1.57 | 3.19 | 0.001 | 0.004 | 2.39 | 2.17 | 1.02 | 1.20 |
| Nfkb2 | 1.58 | 2.49 | 0.013 | 0.037 | 1.31 | 2.04 | 1.57 | 1.58 |
| Osmr | 1.58 | 2.47 | 0.014 | 0.039 | 3.74 | 2.68 | 0.33 | 1.65 |
| Btn1a1 | 1.59 | 2.43 | 0.015 | 0.043 | 1.62 | 2.56 | 1.81 | 0.94 |
| Irf5 | 1.62 | 3.01 | 0.003 | 0.008 | 1.66 | 1.68 | 1.10 | 1.73 |
| Gnaq | 1.63 | 2.42 | 0.016 | 0.045 | 1.77 | 1.67 | 4.42 | 0.80 |
| Sulf2 | 1.64 | 2.61 | 0.009 | 0.027 | 1.55 | 1.55 | 4.98 | 1.64 |
| Slc7a11 | 1.66 | 2.56 | 0.011 | 0.031 | 1.80 | 1.88 | 1.36 | 1.63 |
| Slamf1 | 1.70 | 2.52 | 0.012 | 0.034 | 4.61 | 1.91 | 1.60 | 0.91 |
| Selp | 1.71 | 2.66 | 0.008 | 0.023 | 2.73 | 3.61 | 0.43 | 2.10 |
| Npy1r | 1.73 | 2.38 | 0.017 | 0.049 | 1.48 | 2.73 | 0.58 | 1.65 |
| Prokr1 | 1.74 | 2.56 | 0.010 | 0.030 | 4.36 | 3.69 | 0.59 | 1.05 |
| Sod2 | 1.74 | 2.59 | 0.010 | 0.028 | 1.88 | 2.53 | 1.55 | 1.60 |
| Serpinb2 | 1.75 | 2.44 | 0.015 | 0.043 | 1.71 | 5.20 | 0.69 | 1.51 |
| Zfp608 | 1.78 | 3.46 | 0.001 | 0.002 | 3.10 | 6.53 | 0.50 | 0.94 |
| Rab32 | 1.78 | 2.89 | 0.004 | 0.012 | 1.55 | 2.16 | 2.14 | 1.77 |
| Ptger4 | 1.79 | 2.58 | 0.010 | 0.029 | 2.67 | 2.87 | 2.39 | 1.73 |
| Stx11 | 1.81 | 3.36 | 0.001 | 0.003 | 3.92 | 4.21 | 0.65 | 0.99 |
| Igsf3 | 1.82 | 2.43 | 0.015 | 0.044 | 6.52 | 1.49 | 1.99 | 0.75 |
| Arhgap27 | 1.83 | 2.43 | 0.015 | 0.043 | 4.85 | 1.76 | 1.36 | 1.01 |
| Nhedc1 | 1.86 | 2.55 | 0.011 | 0.031 | 4.59 | 3.99 | 1.02 | 0.99 |
| Adora2b | 1.89 | 2.92 | 0.004 | 0.011 | 1.66 | 7.58 | 0.46 | 1.50 |
| Tbc1d9 | 1.91 | 2.44 | 0.015 | 0.042 | 6.53 | 4.59 | 1.78 | 1.54 |
| Tnip1 | 1.95 | 2.39 | 0.017 | 0.048 | 1.65 | 1.98 | 4.07 | 1.44 |
| Slamf8 | 1.96 | 2.66 | 0.008 | 0.024 | 3.00 | 4.34 | 1.26 | 1.93 |
| Actr3b | 1.99 | 2.38 | 0.017 | 0.050 | 10.98 | 3.29 | 0.63 | 0.93 |
| Pim1 | 2.03 | 2.63 | 0.008 | 0.025 | 2.64 | 1.46 | 4.79 | 2.03 |
| Map3k8 | 2.03 | 3.09 | 0.002 | 0.006 | 4.01 | 2.35 | 1.04 | 1.63 |
| Polr3k | 2.09 | 2.43 | 0.015 | 0.043 | 6.27 | 1.76 | 1.82 | 1.12 |
| Bcl3 | 2.15 | 3.05 | 0.002 | 0.007 | 1.90 | 2.38 | 1.62 | 2.16 |
| Hoxd13 | 2.19 | 3.50 | 0.000 | 0.002 | 4.72 | 5.30 | 0.98 | 0.99 |
| Cdca2 | 2.20 | 2.43 | 0.015 | 0.043 | 3.84 | 3.99 | 1.53 | 1.75 |
| Rnd1 | 2.22 | 2.90 | 0.004 | 0.012 | 8.56 | 2.31 | 1.28 | 0.97 |
| Lass5 | 2.27 | 2.39 | 0.017 | 0.049 | 17.77 | 1.18 | 1.34 | 0.98 |
| Gpr171 | 2.28 | 2.52 | 0.012 | 0.034 | 7.73 | 4.22 | 1.06 | 1.53 |
| Ptgs2 | 2.28 | 2.43 | 0.015 | 0.043 | 9.00 | 4.69 | 1.23 | 1.21 |
| Mpa2l | 2.33 | 2.91 | 0.004 | 0.011 | 1.77 | 4.99 | 1.18 | 2.35 |
| Nfatc2 | 2.41 | 2.43 | 0.015 | 0.044 | 3.98 | 2.48 | 3.38 | 1.50 |
| B3gat1 | 2.49 | 2.50 | 0.013 | 0.036 | 2.84 | 9.13 | 1.50 | 1.04 |
| Ch25h | 2.53 | 2.53 | 0.011 | 0.033 | 2.00 | 1.95 | 2.44 | 3.93 |
| Il33 | 2.63 | 2.48 | 0.013 | 0.037 | 1.95 | 4.11 | 6.69 | 1.51 |
| Slc5a1 | 2.68 | 2.43 | 0.015 | 0.043 | 2.39 | 8.81 | 1.73 | 3.01 |
| Nfkbie | 2.76 | 3.95 | 0.000 | 0.000 | 4.11 | 3.48 | 1.02 | 2.44 |
| Socs3 | 2.81 | 3.29 | 0.001 | 0.003 | 5.54 | 2.23 | 2.57 | 2.06 |
| Elf3 | 2.88 | 4.02 | 0.000 | 0.000 | 5.82 | 3.67 | 2.36 | 1.56 |
| Sele | 3.09 | 2.87 | 0.004 | 0.013 | 13.21 | 3.35 | 1.84 | 2.39 |
| Tnfaip2 | 3.17 | 4.70 | 0.000 | 0.000 | 10.26 | 5.73 | 1.24 | 1.72 |
| Tlr2 | 3.38 | 4.39 | 0.000 | 0.000 | 5.45 | 7.87 | 1.46 | 2.24 |
| Chl1 | 3.69 | 2.69 | 0.007 | 0.022 | 3.64 | 3.62 | 2.78 | 3.82 |
| Ccl2 | 3.75 | 4.74 | 0.000 | 0.000 | 4.31 | 3.22 | 0.98 | 9.46 |
| Steap4 | 4.33 | 2.79 | 0.005 | 0.016 | 1.81 | 10.55 | 1.77 | 9.12 |
| Tnfaip3 | 4.34 | 2.55 | 0.011 | 0.032 | 12.92 | 2.30 | 7.06 | 1.73 |
| Ccl7 | 4.40 | 5.92 | 0.000 | 0.000 | 5.88 | 6.44 | 0.46 | 7.69 |
| Zc3h12a | 4.41 | 4.69 | 0.000 | 0.000 | 11.47 | 13.51 | 1.67 | 1.48 |
| Il6 | 4.66 | 2.84 | 0.004 | 0.014 | 11.46 | 12.43 | 1.52 | 3.41 |
| Vcam1 | 4.97 | 4.67 | 0.000 | 0.000 | 21.05 | 9.89 | 2.03 | 1.53 |
| Nfkbiz | 5.41 | 3.80 | 0.000 | 0.001 | 43.62 | 4.12 | 2.01 | 2.76 |
| Cxcl1 | 7.21 | 3.11 | 0.002 | 0.006 | 7.42 | 3.17 | 4.16 | 24.04 |
| Ccl20 | 30.64 | 3.30 | 0.001 | 0.003 | 80.69 | 94.66 | 27.58 | 5.28 |

## Slide 14
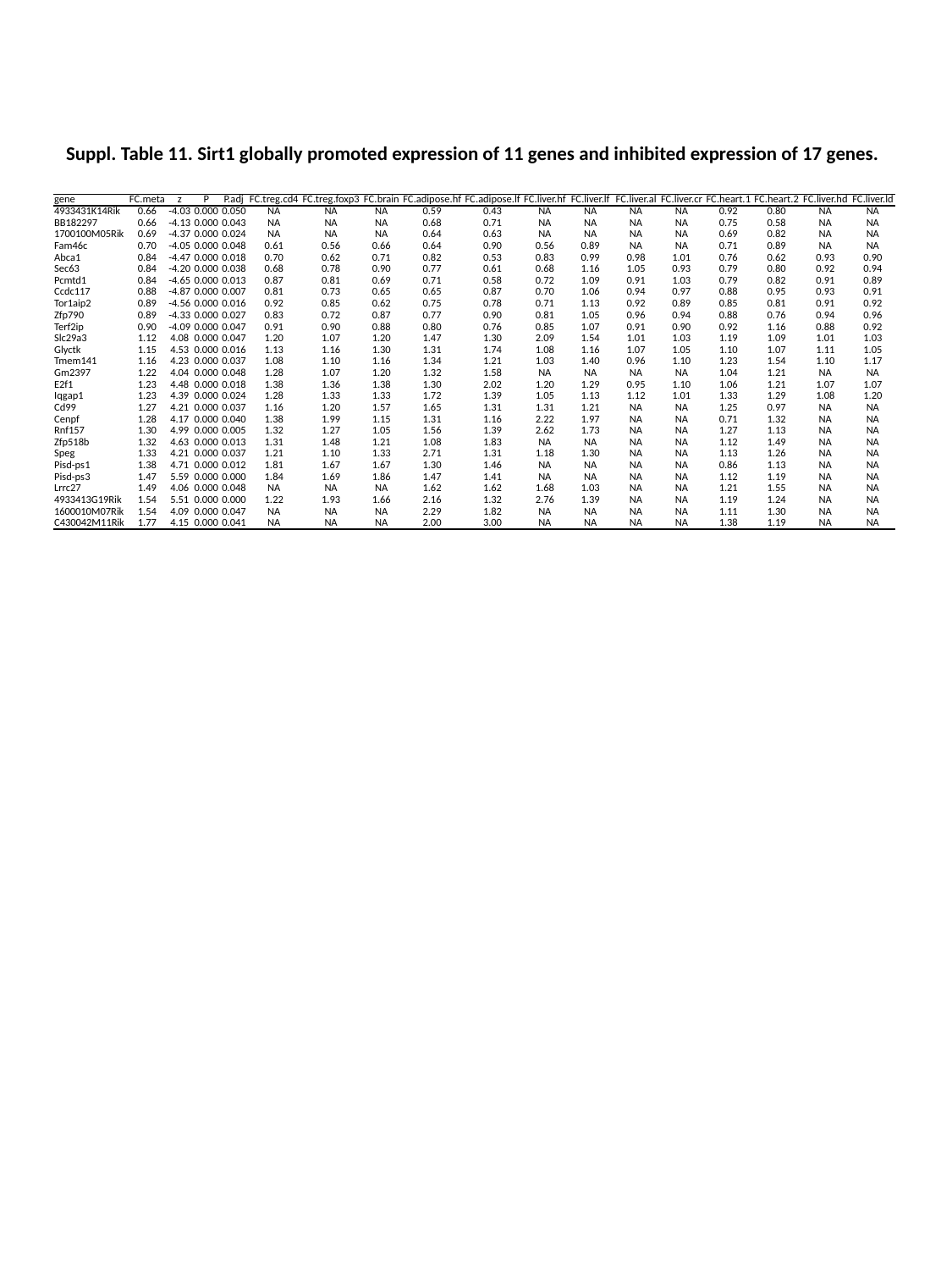

Suppl. Table 11. Sirt1 globally promoted expression of 11 genes and inhibited expression of 17 genes.
| gene | FC.meta | z | P | P.adj | FC.treg.cd4 | FC.treg.foxp3 | FC.brain | FC.adipose.hf | FC.adipose.lf | FC.liver.hf | FC.liver.lf | FC.liver.al | FC.liver.cr | FC.heart.1 | FC.heart.2 | FC.liver.hd | FC.liver.ld |
| --- | --- | --- | --- | --- | --- | --- | --- | --- | --- | --- | --- | --- | --- | --- | --- | --- | --- |
| 4933431K14Rik | 0.66 | -4.03 | 0.000 | 0.050 | NA | NA | NA | 0.59 | 0.43 | NA | NA | NA | NA | 0.92 | 0.80 | NA | NA |
| BB182297 | 0.66 | -4.13 | 0.000 | 0.043 | NA | NA | NA | 0.68 | 0.71 | NA | NA | NA | NA | 0.75 | 0.58 | NA | NA |
| 1700100M05Rik | 0.69 | -4.37 | 0.000 | 0.024 | NA | NA | NA | 0.64 | 0.63 | NA | NA | NA | NA | 0.69 | 0.82 | NA | NA |
| Fam46c | 0.70 | -4.05 | 0.000 | 0.048 | 0.61 | 0.56 | 0.66 | 0.64 | 0.90 | 0.56 | 0.89 | NA | NA | 0.71 | 0.89 | NA | NA |
| Abca1 | 0.84 | -4.47 | 0.000 | 0.018 | 0.70 | 0.62 | 0.71 | 0.82 | 0.53 | 0.83 | 0.99 | 0.98 | 1.01 | 0.76 | 0.62 | 0.93 | 0.90 |
| Sec63 | 0.84 | -4.20 | 0.000 | 0.038 | 0.68 | 0.78 | 0.90 | 0.77 | 0.61 | 0.68 | 1.16 | 1.05 | 0.93 | 0.79 | 0.80 | 0.92 | 0.94 |
| Pcmtd1 | 0.84 | -4.65 | 0.000 | 0.013 | 0.87 | 0.81 | 0.69 | 0.71 | 0.58 | 0.72 | 1.09 | 0.91 | 1.03 | 0.79 | 0.82 | 0.91 | 0.89 |
| Ccdc117 | 0.88 | -4.87 | 0.000 | 0.007 | 0.81 | 0.73 | 0.65 | 0.65 | 0.87 | 0.70 | 1.06 | 0.94 | 0.97 | 0.88 | 0.95 | 0.93 | 0.91 |
| Tor1aip2 | 0.89 | -4.56 | 0.000 | 0.016 | 0.92 | 0.85 | 0.62 | 0.75 | 0.78 | 0.71 | 1.13 | 0.92 | 0.89 | 0.85 | 0.81 | 0.91 | 0.92 |
| Zfp790 | 0.89 | -4.33 | 0.000 | 0.027 | 0.83 | 0.72 | 0.87 | 0.77 | 0.90 | 0.81 | 1.05 | 0.96 | 0.94 | 0.88 | 0.76 | 0.94 | 0.96 |
| Terf2ip | 0.90 | -4.09 | 0.000 | 0.047 | 0.91 | 0.90 | 0.88 | 0.80 | 0.76 | 0.85 | 1.07 | 0.91 | 0.90 | 0.92 | 1.16 | 0.88 | 0.92 |
| Slc29a3 | 1.12 | 4.08 | 0.000 | 0.047 | 1.20 | 1.07 | 1.20 | 1.47 | 1.30 | 2.09 | 1.54 | 1.01 | 1.03 | 1.19 | 1.09 | 1.01 | 1.03 |
| Glyctk | 1.15 | 4.53 | 0.000 | 0.016 | 1.13 | 1.16 | 1.30 | 1.31 | 1.74 | 1.08 | 1.16 | 1.07 | 1.05 | 1.10 | 1.07 | 1.11 | 1.05 |
| Tmem141 | 1.16 | 4.23 | 0.000 | 0.037 | 1.08 | 1.10 | 1.16 | 1.34 | 1.21 | 1.03 | 1.40 | 0.96 | 1.10 | 1.23 | 1.54 | 1.10 | 1.17 |
| Gm2397 | 1.22 | 4.04 | 0.000 | 0.048 | 1.28 | 1.07 | 1.20 | 1.32 | 1.58 | NA | NA | NA | NA | 1.04 | 1.21 | NA | NA |
| E2f1 | 1.23 | 4.48 | 0.000 | 0.018 | 1.38 | 1.36 | 1.38 | 1.30 | 2.02 | 1.20 | 1.29 | 0.95 | 1.10 | 1.06 | 1.21 | 1.07 | 1.07 |
| Iqgap1 | 1.23 | 4.39 | 0.000 | 0.024 | 1.28 | 1.33 | 1.33 | 1.72 | 1.39 | 1.05 | 1.13 | 1.12 | 1.01 | 1.33 | 1.29 | 1.08 | 1.20 |
| Cd99 | 1.27 | 4.21 | 0.000 | 0.037 | 1.16 | 1.20 | 1.57 | 1.65 | 1.31 | 1.31 | 1.21 | NA | NA | 1.25 | 0.97 | NA | NA |
| Cenpf | 1.28 | 4.17 | 0.000 | 0.040 | 1.38 | 1.99 | 1.15 | 1.31 | 1.16 | 2.22 | 1.97 | NA | NA | 0.71 | 1.32 | NA | NA |
| Rnf157 | 1.30 | 4.99 | 0.000 | 0.005 | 1.32 | 1.27 | 1.05 | 1.56 | 1.39 | 2.62 | 1.73 | NA | NA | 1.27 | 1.13 | NA | NA |
| Zfp518b | 1.32 | 4.63 | 0.000 | 0.013 | 1.31 | 1.48 | 1.21 | 1.08 | 1.83 | NA | NA | NA | NA | 1.12 | 1.49 | NA | NA |
| Speg | 1.33 | 4.21 | 0.000 | 0.037 | 1.21 | 1.10 | 1.33 | 2.71 | 1.31 | 1.18 | 1.30 | NA | NA | 1.13 | 1.26 | NA | NA |
| Pisd-ps1 | 1.38 | 4.71 | 0.000 | 0.012 | 1.81 | 1.67 | 1.67 | 1.30 | 1.46 | NA | NA | NA | NA | 0.86 | 1.13 | NA | NA |
| Pisd-ps3 | 1.47 | 5.59 | 0.000 | 0.000 | 1.84 | 1.69 | 1.86 | 1.47 | 1.41 | NA | NA | NA | NA | 1.12 | 1.19 | NA | NA |
| Lrrc27 | 1.49 | 4.06 | 0.000 | 0.048 | NA | NA | NA | 1.62 | 1.62 | 1.68 | 1.03 | NA | NA | 1.21 | 1.55 | NA | NA |
| 4933413G19Rik | 1.54 | 5.51 | 0.000 | 0.000 | 1.22 | 1.93 | 1.66 | 2.16 | 1.32 | 2.76 | 1.39 | NA | NA | 1.19 | 1.24 | NA | NA |
| 1600010M07Rik | 1.54 | 4.09 | 0.000 | 0.047 | NA | NA | NA | 2.29 | 1.82 | NA | NA | NA | NA | 1.11 | 1.30 | NA | NA |
| C430042M11Rik | 1.77 | 4.15 | 0.000 | 0.041 | NA | NA | NA | 2.00 | 3.00 | NA | NA | NA | NA | 1.38 | 1.19 | NA | NA |
